# Genome Size as a Key to Evolutionary Complex Aquatic Plants: Polyploidy and Hybridization in *Callitriche* (Plantaginaceae)

**DOI:** 10.1371/journal.pone.0105997

**Published:** 2014-09-11

**Authors:** Jan Prančl, Zdeněk Kaplan, Pavel Trávníček, Vlasta Jarolímová

**Affiliations:** 1 Institute of Botany, Academy of Sciences of the Czech Republic, Průhonice, Czech Republic; 2 Department of Botany, Charles University, Praha, Czech Republic; University of Delhi, India

## Abstract

Despite their complex evolutionary histories, aquatic plants are highly underrepresented in contemporary biosystematic studies. Of them, the genus *Callitriche* is particularly interesting because of such evolutionary features as wide variation in chromosome numbers and pollination systems. However, taxonomic difficulties have prevented broader investigation of this genus. In this study we applied flow cytometry to *Callitriche* for the first time in order to gain an insight into evolutionary processes and genome size differentiation in the genus. Flow cytometry complemented by confirmation of chromosome counts was applied to an extensive dataset of 1077 *Callitriche* individuals from 495 localities in 11 European countries and the USA. Genome size was determined for 12 taxa. The results suggest that many important processes have interacted in the evolution of the genus, including polyploidization and hybridization. Incongruence between genome size and ploidy level, intraspecific variation in genome size, formation of autotriploid and hybridization between species with different pollination systems were also detected. Hybridization takes place particularly in the diploid – tetraploid complex *C. cophocarpa* – *C. platycarpa*, for which the triploid hybrids were frequently recorded in the area of co-occurrence of its parents. A hitherto unknown hybrid (probably *C. hamulata* × *C. cophocarpa*) with a unique chromosome number was discovered in the Czech Republic. However, hybridization occurs very rarely among most of the studied species. The main ecological preferences were also compared among the taxa collected. Although *Callitriche* taxa often grow in mixed populations, the ecological preferences of individual species are distinctly different in some cases. Anyway, flow cytometry is a very efficient method for taxonomic delimitation, determination and investigation of *Callitriche* species, and is even able to distinguish homoploid taxa and identify introduced species.

## Introduction

Aquatic plants are characterized by several specific adaptations to the water environment, including considerable morphological reduction, prolific clonal propagation and extensive phenotypic plasticity (e.g. [1]–[10]). These characteristics can make classification of these plants particularly difficult, with this difficulty increased by the frequent parallel evolution of traits in unrelated taxa [11]–[16]. Indeed, aquatic plants are regarded as being among the most taxonomically challenging angiosperms.

Aquatic plants play a key ecological role in aquatic ecosystems and often exhibit complex evolutionary histories. Polyploidy and hybridization have been crucial to the evolution of many aquatic plant groups [7], namely in *Ranunculus* subg. *Batrachium* [11], [17]–[20], *Potamogeton* [15], [16], [21]–[26], *Lemnaceae* [27]–[28], *Nymphaea* [29]–[30], *Elodea* [31], *Myriophyllum* [32] and many others. Intrageneric chromosome number variation has been reported from 80% and hybridization from 20% of all aquatic plant genera [7]. Newly established polyploids and hybrids can be fixed by frequent clonal growth, with sterile primary hybrid genotypes persisting hundreds or even thousands of years without the presence of the parental species [16], [33]–[37]. However the new taxa that arise through these processes are often morphologically undetectable. Indeed, in general, a substantial part of the variation in aquatic plants is undoubtedly cryptic and detectable only using molecular techniques.

Despite the interesting evolutionary scenarios posed by aquatic plants, they are markedly underrepresented in contemporary biosystematic studies [5] – likely due in large part to the daunting challenges associated with these scenarios, as well as the difficulty in detecting taxonomic differences. Thus, our overall knowledge of the principal processes that have driven evolution of aquatic taxa is limited, especially in comparison with the information developed regarding terrestrial plants. This has limited our potential to understand the wider context of evolution and systematics of these plants.

In the current study, we examine the aquatic genus *Callitriche* (water-starwort) which comprises about 60 species throughout the world. In Europe, about 15 native and 4 rare, introduced species have been reported [38]–[40]. *Callitriche* is notoriously considered one of the most difficult aquatic plants to identify. Taxonomy of *Callitriche* is based mostly on the generative features, particularly fruits. Unfortunately, these characters are very small, difficult to observe and often not available due to the frequent occurrence (or even prevalence) of solely vegetative plants. Virtually all vegetative characters of water-starworts are extremely variable and mostly unusable without extensive experience. Therefore, although many detailed morphological studies on water-starworts have been published (e.g. [38], [40]–[50]), their reliable determination is still restricted to just a few specialists, with several taxa recognizable only in the case of well-developed, adult individuals.

Water-starworts, among the most common aquatic plants throughout Europe, inhabit almost all types of standing and running waters, even including small puddles on forest paths. Despite this, they are generally overlooked by field biologists, even though individual *Callitriche* species can differ substantially in their ecology and may serve as diagnostic taxa of various phytosociological units [49], [50]. Water-starworts may also represent suitable model organisms for the study of phenotypic plasticity [51]–[54], physiological processes associated with growth [55]–[57], metabolism in aquatic environments [58]–[61], plant patch formation in streams [62], [63], phytoremediation of polluted watercourses [64], and some taxa may even serve as a potential antioxidant-rich diet supplement [65], [66]. Pollination biology of water-starworts is strikingly diversified and among the most remarkable of all the angiosperms [45], [67]–[70]. However, *Callitriche* is still only rarely a subject of scientific research due to taxonomic and methodological difficulties (e.g., observation, scoring, cultivation, and experimentation). Finding a method that allows easy and reliable determination of water-starworts in various developmental stages would therefore promote exploration of many aspects of aquatic and wetland ecosystems.

Evolution of the genus has featured recurrent polyploidization and aneuploid reduction of chromosome numbers in various lineages [70]. Therefore, chromosome counting has often been used for the genus, especially in Europe. To date, chromosome numbers for 35 taxa are available (summarized in Table S1) and 11 different chromosome counts are known, ranging from 2n = 6 to 2n = 40. The most common diploid chromosome number is 2n = 10, but the diploids 2n = 6 and 2n = 8 have also been found in some species.

In Europe, all three diploid chromosome numbers are known, and at least four polyploid species occur (*C. palustris* 2n = 20, *C. platycarpa* 2n = 20, *C. brutia* 2n = 28, *C. hamulata* 2n = 38). *Callitriche platycarpa* is considered to be an allotetraploid that has arisen through the hybridization of the diploid species *C. cophocarpa* and *C. stagnalis* [71], [72], confirmed by allozyme analysis of plants from north-western Poland [73]. The origin of other European polyploids is unknown.

Recent hybridization has also been detected in the genus: the triploid (2n = 15) *Callitriche* ×*vigens* (*C. cophocarpa* × *C. platycarpa*), the sole primary hybrid currently known and validly described, has repeatedly been found in areas of co-occurrence of the parental species (e.g. [34], [47], [74], [75]). However, to-date this hybrid has never been confirmed by molecular studies. More generally, the lack of molecular investigation of this genus leaves open the possibility of undiscovered hybrids within it. In particular, the amount of hybridization between taxa with the same ploidy levels would have escaped detection by chromosome counting.

To date, however, chromosome counting has remained the exclusive cytogenetic method used. However, the potential utility of chromosome counting *per se* for taxa determination is limited, because only two European (*C. brutia*, *C. hamulata*) and a single African (*C. vulcanicola*) species possess unique chromosome numbers. The phylogenetic relationships between most of the taxa remain unclear because molecular techniques have been only sporadically applied to *Callitriche*. These include phylogenetic analysis of European and North American species ([70], unfortunately using rbcL as a marker, which is not sufficiently variable) as well as allozyme and RAPD analyses on a small geographic scale [73], [76]–[78]. The combination of rbcL and ITS applied to northern Italian *Callitriche* [79] was unfortunately not supported by adequate determination of plants, and the data interpretation in this study is largely questionable.

The variation in chromosome numbers in *Callitriche* encourages use of the genome size as a species-specific marker. Flow cytometry (FCM), which has undergone a boom in plant sciences over the last decade, represents an excellent tool for this purpose. FCM is a rapid, easy, statistically robust and relatively cheap method [80], [81], frequently and successfully applied to evolutionarily and taxonomically intricate plant groups such as polyploid complexes [82]–[86]. Due to the high accuracy of the measurements, FCM is often able to distinguish even closely related homoploid taxa (reviewed in [87]), and it is also frequently used for detection of hybrids [87]–[91]. An indisputable benefit of FCM is its facility to analyze a large number of individuals (e.g., at a population level) rapidly, even allowing detection of rarely occurring cytotypes, hybrids and aneuploids ([92]–[95], etc.). Finally, flow cytometry permits analyses using a very small amount of plant material. This is extremely useful in the aquatic environment, where researchers often find only small vegetative fragments.

Unfortunately, flow cytometry has so far been only sporadically applied to research on aquatic plants (Nymphaeales [96], [97]; *Nymphaea* [98], [99]; *Cabomba* [100], [101]; *Rorippa* [102]; *Nasturtium* [103]; Lemnaceae [104]; *Zannichellia* [105]). To date, no studies using FCM are available for *Callitriche*. In fact, the genome size has been estimated only once for Western European water-starwort species, using photometric cytometry with the Feulgen staining method [106]. However, this method cannot process large numbers of samples and is unable to reliably distinguish small differences in genome size among taxa with the same ploidy level.

In this study, we applied flow cytometry combined with chromosome counting to improve our understanding and identification of Central European water-starworts. The following aims were addressed: (1) testing flow cytometry as a method for reliable determination of the Central European *Callitriche* species; (2) determining the chromosome numbers based on cytometrically analysed samples; (3) comparing the new counts with previously published records; and (4) estimating the hybridization rate in the studied area.

## Materials and Methods

### Field sampling

Plant samples were collected in Belgium, Czech Republic (majority of samples), Denmark, Germany, Hungary, Italy, Netherlands, Norway, Poland, Slovakia and Sweden in 2007–2014. Our sampling included all seven Central European species, several specimens of hybrid origin (see below) and also the Mediterranean species *C. lenisulca*, which is apparently closely related to the Central European *C. cophocarpa* [107]. In addition, we included seven samples of European species that were collected in the western USA (*C. stagnalis* and *C. hamulata*, both introduced, and *C. palustris*, native to both Europe and North America). In total, 1076 plants from 494 localities were obtained (for locality details, see Table S2). Voucher specimens are preserved in the herbarium of the Charles University in Prague (acronym PRC).

We put an emphasis on visiting the widest possible range of aquatic habitats, including small and commonly neglected biotopes (e.g., puddles on forest paths, eutrophic ditches). At each locality, sampling covered observed morphological variation. The sampling was carried out even in stands comprising only sterile plants. The fresh plant material was placed in plastic bags and transported rapidly to the FCM laboratory. In the cases of longer transport, plants were wrapped in moist paper towels (not too wet, in order to avoid rotting) and then sealed in plastic bags. Aquatic plants preserved in this way stay fresh for 2–3 weeks, enabling analysis of samples even from distant regions.

### Flow cytometry

All 1077 plants were analysed using FCM. If multiple samples were collected from a population, these samples were first analysed simultaneously (approximately 5 samples in a single run) using 4′,6-diamidino-2-phenylindole (DAPI) fluorochrome to reveal the possible presence of multiple cytotypes. About 0.25 cm^2^ of leaf tissue was chopped together with an appropriate volume of the internal standard using a sharp razor blade in a Petri dish containing 0.5 ml of ice-cold Otto I buffer (0.1 M citric acid, 0.5% Tween 20, [108]). *Bellis perennis* L. was selected as a primary reference standard, as it has a similar, but non-overlapping genome size with the majority of the studied samples (2C = 3.96 pg, [109]; 2C-value was calibrated according to the following internal standard). *Glycine max* (L.) Merr. ‘Polanka’ (2C = 2.50 pg, [80]) served as a reference standard for *Callitriche palustris* and *C. obtusangula*, because the genome size of these taxa overlapped with *Bellis*. The crude suspension was filtered through a 42-μm nylon mesh and incubated for ca 5 min. at room temperature. After incubation, isolated nuclei were stained with 1 mL of Otto II buffer (0.4 M Na_2_HPO_4_.12H_2_O) supplemented with DAPI (4 µg/ml) and β-mercaptoethanol (2 µl/ml). Samples were run on the flow cytometer after about one minute of staining, using a Partec PA II flow cytometer (Partec GmbH, Münster, Germany) equipped with a mercury arc lamp as the UV light excitation source. The fluorescence intensity of 3000 particles was recorded. Histograms were evaluated using FloMax software, ver. 2.4d (Partec GmbH).

Subsequently, all detected cytotypes from all populations were analyzed separately using propidium iodide FCM in order to estimate the variation in genome size. To determine the genome sizes for particular taxa in absolute units (pg of DNA), 178 samples representing all cytotypes were measured at least three times on different days to account for occasional fluctuations. Because genome size generally differed substantially between taxa (see below), a further 416 samples were each measured only once as a single measurement was sufficient for reliable assignment. The staining solution consisted of 1 mL of Otto II buffer supplemented with propidium iodide (50 µg/ml), RNase A, type IIA (50 µg/ml) and β-mercaptoethanol (2 µl/ml). The fluorescence intensity of 5000 particles was recorded using a Partec CyFlow instrument equipped with a green diode-pumped solid-state laser (Cobolt Samba, 532 nm, 150 mW output power). If the range in variation of the three measurements exceeded the 2% threshold, the outlying value was discarded and the sample re-analysed.

Because DAPI FCM was not able to distinguish *C. cophocarpa* and *C. stagnalis* in simultaneous analyses, the samples were measured individually using propidium iodide staining when either of these species was suspected.

### Morphological identification

Sufficiently developed plants exhibiting essential morphological characters were identified based on recent morphological studies [40], [49], [50]. These determinations were then compared with genome sizes obtained from FCM analysis, with discordance suggesting hybrid plants. Identification of juvenile and sterile samples was facilitated using FCM. Species for which overlapping or very similar 2C-values were found even when using propidium iodide staining (see below), were identified by the most relevant morphological characters: *C. palustris* and *C. obtusangula* were distinguished on the basis of conspicuously different fruits, and *C. obtusangula* by its remarkable elongate-ellipsoid and curved pollen grains. Sterile plants with 2C-values within the ranges of variation of these two species were cultivated until they produced characters necessary for unambiguous identification. *Callitriche lenisulca* was distinguished from Italian samples of *C. obtusangula* through its unique flower pattern (nodes with only male or female flowers alternating approximately regularly along the stem), small stamens and anthers, and spherical pollen grains [40], [45].

### Chromosome counts

The chromosome number was counted in at least one sample of each detected cytotype. Selected plants were cultivated in a tank (depth about 20 cm) until they began to form adventive roots on the stem. These adventive roots were used for chromosome counting.

The root tips were pre-treated in a saturated water solution of p-dichlorbenzene for approximately two hours, then fixed in a 3∶1 mixture of 96% ethanol and acetic acid, macerated in a 1∶1 mixture of ethanol and hydrochloric acid for 30 s, washed in water and stained with lacto-propionic orcein. The number of chromosomes was determined under a Carl Zeiss Jena NU microscope equipped with an Olympus Camedia C-2000 Z camera and Olympus E – 510 Digital SRL Camera.

Altogether, chromosome numbers of seven taxa were determined. The remaining three taxa (*C. hermaphroditica*, *C. lenisulca*, autotriploid *C. stagnalis*) failed to grow in cultivation.

### Data analysis

Differences in genome sizes between particular taxa were tested by Bonferroni (Dunn) t Test (α = 5%) using the SAS 9.2 statistical package (SAS Institute, Cary, NC, USA) and depicted as boxplots in STATISTICA. Distribution map was created using DMAP for Windows [110].

To compare the ecological preferences and co-occurrence of individual taxa, all localities were divided on the basis of habitat into the following seven categories (see Table S2): *ditch* (artificial depression/channel with muddy bottom and standing or almost standing eutrophic water), *exposed bottom* (of drained water body or from coastal zone), *fishpond* (artificial water body established for fish farming), *lake* (large natural water body), *pool* (small water body with standing water, natural or arising spontaneously after human disturbances), *puddle* (very small and shallow temporary accumulation of water, usually on paths, with substrate remaining wet after surface water disappearance), *reservoir* (moderate to large artificial water body, not for fish farming), *river* (flowing watercourse with high flow rates; subdivided into two subcategories: *mud* – muddy bottom, *sand* – sandy bottom) and *stream* (small to moderate flowing watercourse; subdivided the same as the previous category).

The categories “fishpond”, “lake” and “reservoir” were subsequently merged into the common category “reservoir”, due to the small number of lakes and reservoirs among the studied localities and the poor ecological differentiation between them.

## Results

### Genome size variation

**Figure 1:**
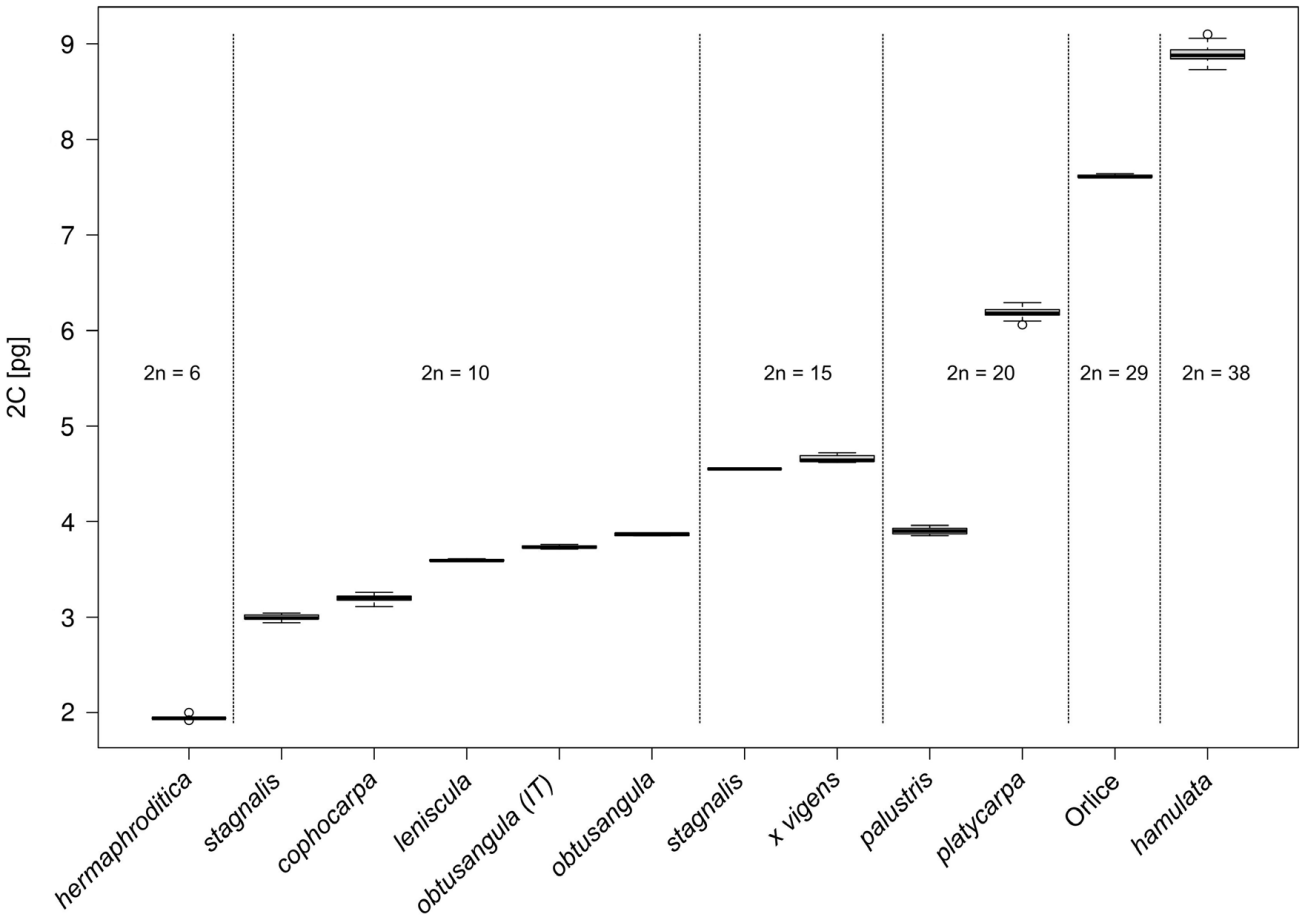
Box-and-whisker plots showing the holoploid genome sizes (2C-values) for 9 *Callitriche* species and two hybrids: *C. cophocarpa* × *C. platycarpa* (*C.* ×*vigens*) and a hybrid (probably *C. hamulata* × unreduced gamete of *C. cophocarpa*) from the Tichá Orlice River, Czech Republic (Orlice). Taxa with different chromosome numbers are separated by vertical lines. For *C. obtusangula*, values for samples from Italian (IT) and north-western Europe were plotted separately, due to significantly different genome sizes.

**Figure 2:**
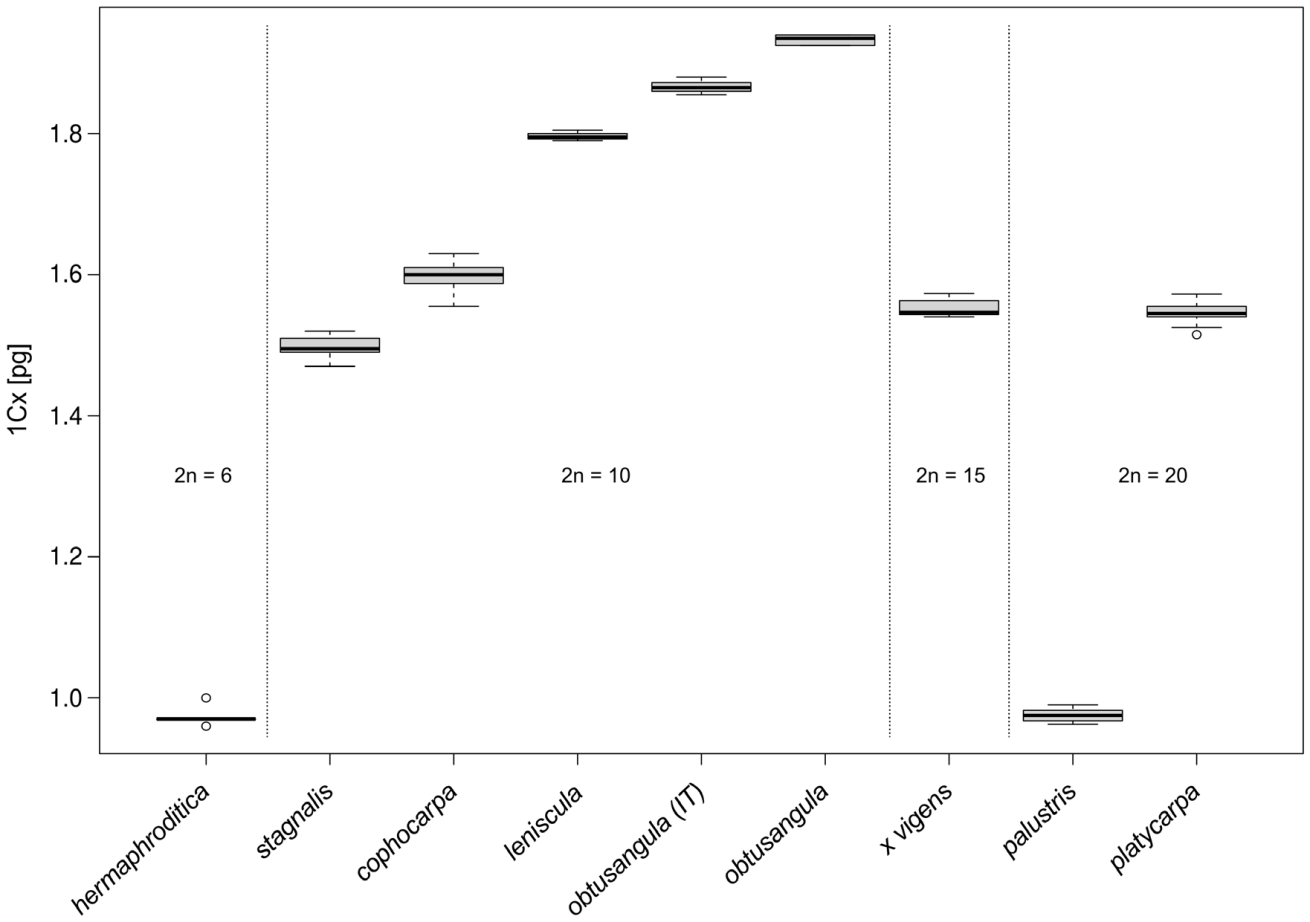
Box-and-whisker plots showing the monoploid genome sizes (1Cx-values) for 9 *Callitriche* taxa. The species *C. hamulata* (2n = 38) and the hybrid from the Tichá Orlice River (2n = 29) were not included due to aneuploid chromosome counts.

**Figure 3:**
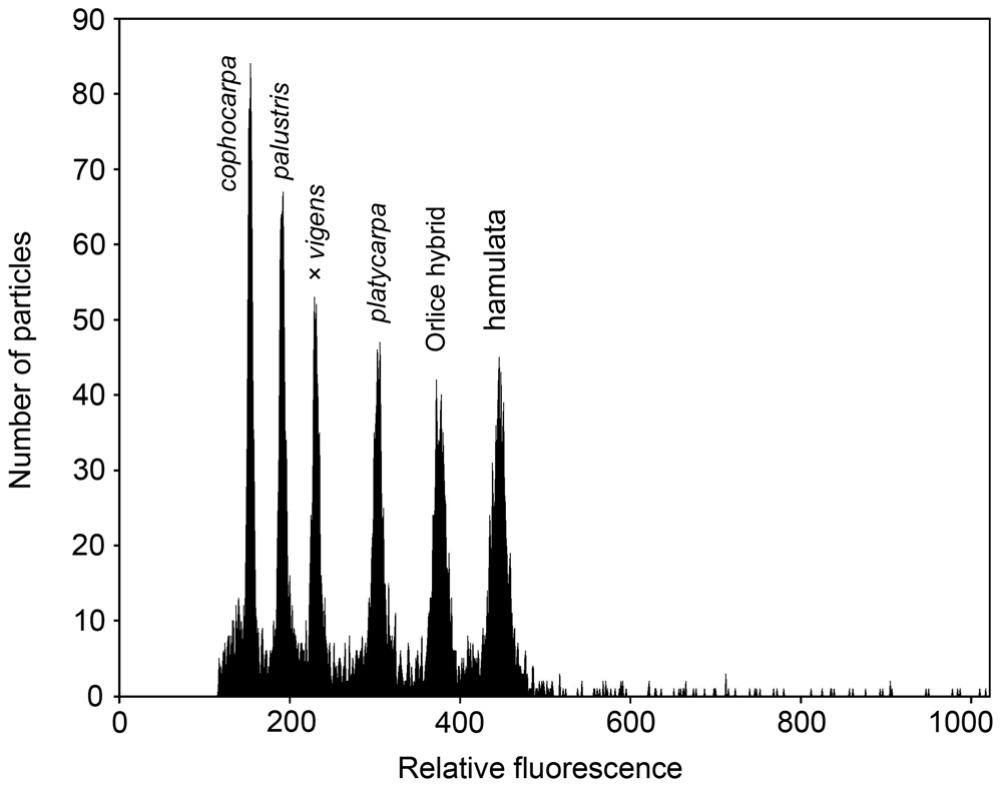
Flow cytometric histogram showing simultaneous analysis of 6 *Callitriche* taxa: *C. cophocarpa* (2n = 10), *C. palustris* (2n = 20), hybrid *C. cophocarpa × C. platycarpa* (*C. ×vigens*, 2n = 15), *C. platycarpa* (2n = 20), hybrid from the Tichá Orlice River (2n = 29) and *C. hamulata* (2n = 38). Nuclei of all samples were isolated, stained with propidium iodide and analysed simultaneously.

In total, 12 taxa of *Callitriche* were recorded, the overwhelming majority of these differing clearly and with statistical significance in nuclear DNA content (Table 1, Figs 1, 2, 3). Among the eight generally recognized species, six can be unambiguously defined by means of genome size (*C. hermaphroditica*, *C. stagnalis*, *C. cophocarpa*, *C. lenisulca*, *C. platycarpa*, *C. hamulata*). The diploid species *C. stagnalis* and *C. cophocarpa* have similar genome sizes (difference between means 7.0%), and simultaneous analyses of these species did not result in double-peaks. However, 2C-values of both these species are non-overlapping and differed significantly in a Bonferroni (Dunn) t Test.

**Table 1:** Flow cytometric results for 178 individuals of 12 *Callitriche* taxa, for which the genome size was determined using propidium iodide staining.

Taxon	Chromosome number (2n)	DNA ploidy level	No. of samples	Mean 2C-value ± s.e. (pg DNA)	2C-value range (pg DNA)	Variation (max/min, %)	Mean 1Cx-value (pg DNA)	Mean chromozome size (pg)	Internal standard*
*C. hermaphroditica*	6	2×	5	1.95±0.03	1.92 – 2.00	3.99	0.98	0.33	B
*C. stagnalis*	10	2×	26	2.99±0.03	2.95 – 3.04	3.40	1.50	0.30	B
*C. cophocarpa*	10	2×	35	3.20±0.04	3.11 – 3.26	4.96	1.60	0.32	B
*C. lenisulca*	10	2×	3	3.59±0.02	3.58 – 3.61	0.89	1.80	0.36	B
*C. obtusangula* (Italy)	probably 10	2×	3	3.73±0.03	3.71 – 3.76	1.35	1.87	0.37	G
*C. obtusangula*	10	2×	5	3.87±0.02	3.85 – 3.88	0.95	1.94	0.39	G
autotriploid *C. stagnalis*	probably 15	3×	1	4.55	–	–	1.52	0.30	B
*C. ×vigens* [*C. cophocarpa × platycarpa*]	15	3×	17	4.66±0.04	4.62 – 4.72	2.26	1.55	0.31	B
*C. palustris*	20	4×	19	3.90±0.04	3.85 – 3.96	2.86	0.98	0.20	G
*C. platycarpa*	20	4×	22	6.18±0.05	6.06 – 6.29	3.73	1.55	0.31	B
hybrid from Tichá Orlice River	29	6×	3	7.62±0.02	7.60 – 7.64	0.53	**	0.26	B
*C. hamulata* ***	38	8×	40	8.89±0.09	8.67 – 9.10	4.95	**	0.23	B

* B  =  *Bellis perennis* (2C = 3.96 pg); G = *Glycine max* ‘Polanka’ (2C = 2.50 pg).

** 1Cx-value cannot be meaningfully calculated due to aneuploid chromosome counts of these taxa.

*** *Callitriche hamulata* has recently been assigned to *C. brutia*, as *C. brutia* var. *hamulata* (Kütz. ex W.D.J. Koch) Lansdown [39], [40]. Both taxa are closely related and can be distinguished morphologically perhaps only in their fertile terrestrial forms. Nevertheless, a thorough study of the entire *C. brutia* complex on a large geographic scale, supported by statistical and analytical methods, is not yet available. *Callitriche brutia* and *C. hamulata* possess distinct chromosome numbers and apparently have different evolutionary histories (although the histories of both species are completely unknown and perhaps complex). In addition, their distribution and habitat requirements are partially different. For these reasons, we retain separate taxonomic treatments of these species, at least until the complex is subjected to a critical review using appropriate genetic markers and the mechanism of its origin elucidated.

The other taxa analysed from Central and Atlantic Europe, *C. palustris* and *C. obtusangula*, surprisingly exhibit very similar, overlapping genome sizes, although they differ in DNA-ploidy level (see below).

**Figure 4:**
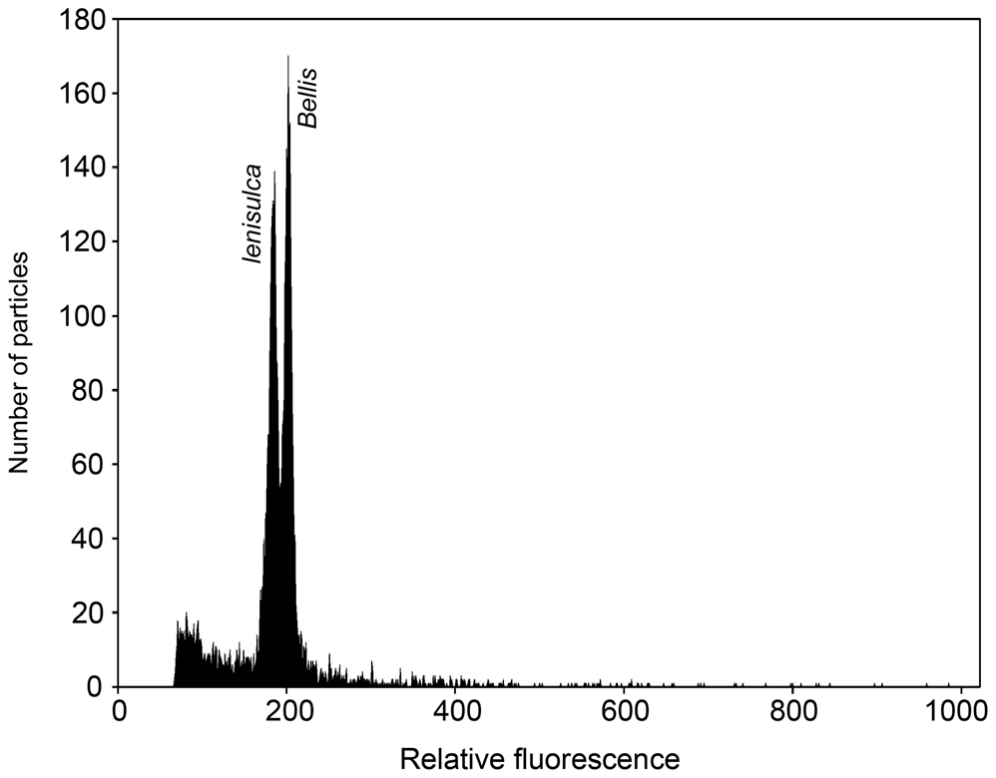
Flow cytometric analysis of *Callitriche lenisulca* with *Bellis perennis* as an internal standard, using propidium iodide staining.

The situation regarding diploid taxa sampled in Italy is more complicated. The Mediterranean species *C. lenisulca* differs significantly in genome size from both *C. cophocarpa* (difference between means 12.2%) and *C. obtusangula* from north-western Europe (difference 7.8%). However, Italian plants, assigned to *C. obtusangula* on the basis of pollen shape and gross morphology, possess a genome size distinct from north-western European plants of this species, with their 2C-value exactly intermediate between *C. lenisulca* and NW *C. obtusangula* (difference from *C. lenisulca* 3.9%, from NW *C. obtusangula* 3.8%; differences are small but statistically significant). On FCM histograms, *C. lenisulca* exhibited a peak clearly distinct from the peak of the internal standard *Bellis perennis* (Fig. 4), whereas both types of *C. obtusangula* always overlapped with *Bellis* and had to be measured with the *Glycine* standard.

In the Czech Republic, Germany and Denmark, non-fertile plants with aborted, deformed, yellow pollen grains were repeatedly found (26 samples). The genome size of these plants lies between some diploid species (*C. stagnalis*, *C. cophocarpa*) and tetraploid *C. platycarpa*, which suggests a triploid DNA-ploidy level. These plants were assigned to the F1 hybrid *C. cophocarpa* × *C. platycarpa* (*C.* ×*vigens*, see Discussion). The exception was the sole plant (specimen C125-13) from northern Bohemia (Czech Republic), which strongly resembled *C. stagnalis* and was partially fertile (but with most mericarps deformed or poorly developed). This plant possessed a slightly (and not significantly) smaller genome size than other triploids, which would better fit an autotriploid of *C. stagnalis*. This inference was confirmed by means of allozyme analysis (J. Prančl *et al.*, unpublished data).

Other plants most likely representing a product of interspecific hybridization (samples C061-12, C065-12, C066-12) were discovered at three sites in the Tichá Orlice River (eastern Bohemia). This hybrid had submerged aborted flowers and colourless, irregular pollen grains with shrunken protoplasts. *Callitriche hamulata* is considered to be one of the parental species, based on its large genome size. The tetraploid *C. platycarpa* (reduced gamete) and the diploids *C. stagnalis* and *C. cophocarpa* (unreduced gametes) are possible as the second parent.

Regarding the European species occurring in North America, *C. palustris* from USA (specimen C048-13) showed the same genome size as all the conspecific samples from Europe. Also *C. stagnalis*, which is naturalized in North America [111], does not differ in genome size from European conspecifics (C053-13, C054-13). Finally, the occurrence of *C. hamulata* on the Pacific coast of the USA [40] was confirmed by the samples from there having genome size identical to that of the European samples of this species (C049-13, C050-13, C051-13, C052-13).

### Chromosome counts

**Figure 5:**
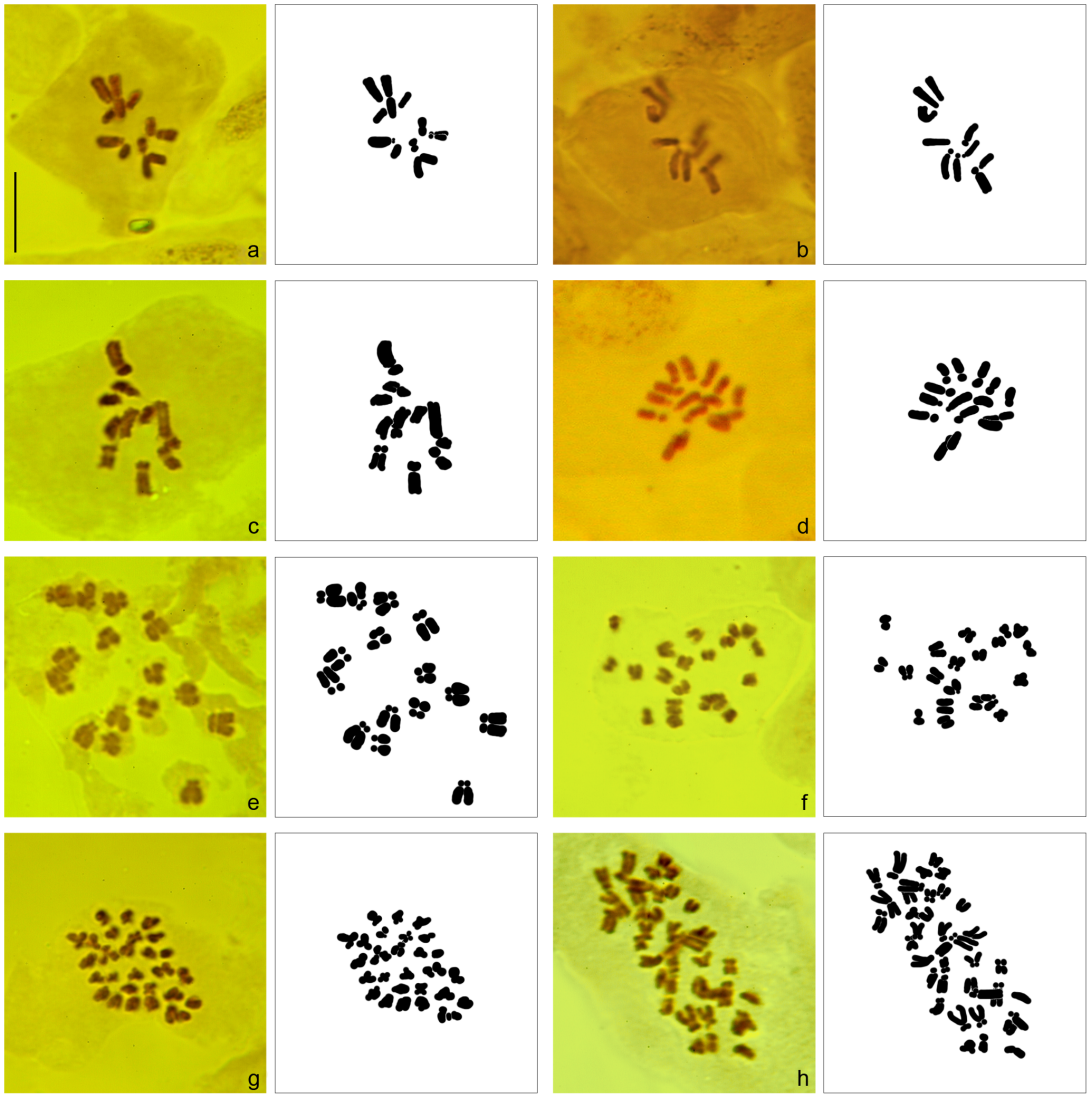
Chromosomes (photograph of the cytological preparation on the left with its interpretation on the right in each pair) of selected species and hybrids of *Callitriche* at mitotic metaphase in somatic cells, arranged according to increasing chromosome number and genome size. a – *C. stagnalis*, specimen C013-12, 2n = 10; b – *C. cophocarpa*, C002-12, 2n = 10; c – *C. obtusangula*, C052-12, 2n = 10; d – *C.* ×*vigens*, C059-08, 2n = 15; e – *C.* ×*vigens*, C021-12, 2n = 15; f – *C. palustris*, C019-12, 2n = 20; g – probable hybrid *C. hamulata* × *C. cophocarpa*, C066-12, 2n = 29; h – *C. hamulata*, C050-13, 2n = 38. Scale bar identical for all figures = 10 µm.

The chromosome number was determined for 12 individuals of 8 taxa (see Table 2, Fig. 5). Chromosome numbers quoted in published sources were confirmed in all studied species. *Callitriche obtusangula* (2n = 10) and *C. palustris* (2n = 20) differ in DNA ploidy level, although they cannot be distinguished by genome size. Triploid chromosome number (2n = 15) was confirmed in plants assigned to the hybrid *C.* ×*vigens*. Non-fertile plants discovered in the Tichá Orlice River possess an extraordinary chromosome number 2n = 29.

**Table 2:** Chromosome numbers of 8 *Callitriche* species counted in this study.

Taxon	Ref. no.*	Country	Chromosome number (2n)
*C. stagnalis*	C013-12	Czech Republic	10
*C. cophocarpa*	C002-12	Czech Republic	10
*C. obtusangula*	C052-12	Netherlands	10
*C. ×vigens* [*C. cophocarpa × C. platycarpa*]	C059-08	Czech Republic	15
	C021-12	Czech Republic	15
*C. palustris*	C019-12	Czech Republic	20
*C. platycarpa*	C011-12	Czech Republic	20
hybrid from Tichá Orlice River	C066-12	Czech Republic	29
*C. hamulata*	C007-12	Czech Republic	ca 38
	C050-13	USA (introduced)	38
	C084-13	Czech Republic	38
	C094-13	Czech Republic	38

*For samples details, see Table S2.

Karyotypes of species with 2n = 10 are different. *Callitriche cophocarpa* has two pairs of slightly bigger acrocentric chromosomes (Fig. 5b). *Callitriche stagnalis* possesses one pair of large metacentric chromosomes; the dimensions of particular chromosomes are the most variable among all the studied diploids (Fig. 5a). *Callitriche obtusangula* has one pair of large acrocentric chromosomes (Fig. 5c).

### Geographical distribution

**Figure 6:**
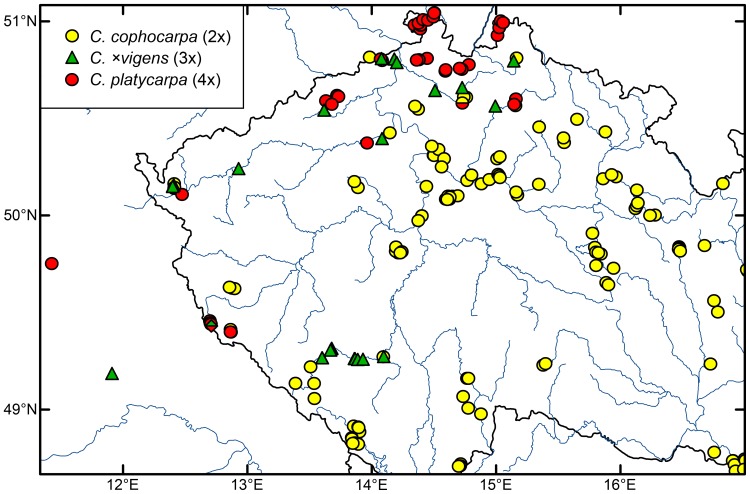
Map of sampled localities of diploid *Callitriche cophocarpa*, tetraploid *C. platycarpa* and their triploid hybrid *C.* ×*vigens* in north-western Czech Republic. Two populations with co-occurrence of two taxa are shown as two-colour bisected symbols.

Four species (*Callitriche cophocarpa*, *C. hamulata*, *C. palustris* and *C. stagnalis*) were recorded as common in Central Europe. Detailed screening performed in the Czech Republic revealed the limits of the distribution of the Subatlantic species *C. platycarpa* in the north-western part of Bohemia (see Fig. 6). Where the range of *C. platycarpa* overlaps that of the related but rather continental species *C. cophocarpa*, many populations were shown to be triploids and assigned to *C. ×vigens*. However, this hybrid was also abundant in the Otava River in southern Bohemia, where *C. platycarpa* has never been found (cf. [50]).

The occurrence of *C. platycarpa* in southern Italy (specimen C010-13) is also noteworthy, because this species was until recently known only from the northernmost Italian regions (cf. [40]). The Mediterranean-Atlantic species *C. obtusangula* was for the first time found in Denmark during this study (cf. [112]; specimens C034-12, C048-12) and the Subatlantic species *C. hamulata* was for the first time reliably recorded from Hungary (specimen C14-001).

### Ecological preferences

The habitat preferences of *Callitriche* taxa from 495 investigated localities are summarized in Table 3. For *C. cophocarpa*, *C. hamulata*, *C. palustris* and *C. stagnalis*, the numbers of localities were sufficiently high (>75) to assess environmental preferences. The strongest relationship with a specific habitat type was recorded for *C. stagnalis*. In total, 78.8% of *C. stagnalis* localities were in puddles. *Callitriche palustris* was found almost exclusively in standing water or on moist, exposed bottoms, with only one locality (1.3%) in running water. In contrast, *C. hamulata* showed the strongest relation to running waters (53.8% of localities). In rivers and streams, bottom substrate can play an important role: streams with *C. hamulata*, predominantly had sandy bottoms (87.0%), whereas those with *C. cophocarpa* had mostly muddy bottoms (75.0%).

**Table 3:** Ecological preferences of 594 *Callitriche* samples of 9 species and 2 hybrids and co-ocurrence of particular taxa in mixed populations.

Taxon	Total no. of populations	Mixed with other taxa	Type of habitat*									Average altitude
			ditch	exposed bottom	pool	puddle	reservoir	river (mud)	river (sand)	stream (mud)	stream (sand)	
*C. cophocarpa*	150	33	23	11	26	28	18	1	1	32	10	398
*C. hamulata*	172	58	1	13	21	17	27	5	45	7	36	411
*C. hermaphroditica*	4	–	–	–	–	–	4	–	–	–	–	442
*C. lenisulca*	3	–	2	–	1	–	–	–	–	–	–	83
*C. obtusangula*	8	–	3	–	–	–	–	–	1	2	2	169
*C. palustris*	76	34	–	22	1	37	15	1	–	–	–	449
*C. platycarpa*	49	17	5	1	4	7	8	2	–	16	6	306
*C. stagnalis*	104	33	5	1	4	82	5	1	–	4	2	413
*C. stagnalis* autotriploid	1	–	–	–	–	1	–	–	–	–	–	525
*C. ×vigens*	25	9	2	1	13	–	5	2	1	–	1	365
hybrid from Tichá Orlice River	3	3	–	–	–	–	–	–	2	–	1	278
Total number of samples	595	187	41	49	70	172	82	12	50	61	58	398
Total number of localities	495	88	39	38	58	135	69	9	46	53	48	399
Mixed localities	88	88	2	9	11	32	11	3	3	8	9	390

* For details, see Materials and Methods.

*Callitriche* taxa often occurred in mixed populations. Thus, 88 localities (17.8% of all localities, 31.5% of all samples) supported more than one taxon. Ten localities (2.0%) supported three taxa, and one locality (0.2%) supported four taxa. Generally, the proportion of mixed localities was highest in *C. palustris* (44.7% of all localities, compared to 34.7% for *C. platycarpa*, 33.9% for *C. hamulata*, 31.7% for *C. stagnalis* and 22.0% for *C. cophocarpa*). The most frequent co-occurrences were the pairs *C. platycarpa* and *C. hamulata* (14 populations, i.e. 28.6% of *C. platycarpa* localities) and *C. palustris* and *C. stagnalis* (17 populations, i.e. 22.7% of *C. palustris* localities).

## Discussion

This study represents the first application of flow cytometry to the genus *Callitriche* and the most comprehensive application of FCM to aquatic angiosperms (in terms of the number of plants collected as well as the number of populations). It has provided insights into methodological, evolutionary, taxonomic, and ecological issues, discussed below.

### Genome size as a tool for identification of *Callitriche* species

Flow cytometry has proven to be a reliable, fast, inexpensive and easy tool to distinguish Central-European *Callitriche* taxa (Table 1, Figs 1, 2, 3). Even homoploid species are recognizable on the basis of genome size. *Callitriche obtusangula* and *C. palustris* are the only two species indistinguishable by this approach. Fortunately, both species differ markedly in fruit shape, size and colour, pollen grain shape, and floating leaf shape [40], and mostly also in the general appearance of the plants. In fact, these two taxa are probably the most distinctive species of *Callitriche* in Europe. Thus, confusion between them is unlikely. Due to the unique mode of self-pollination (internal geitonogamy; [113]), individuals of *C. palustris* are almost always abundantly fertile [49], greatly facilitating determination.

Despite the very similar genome sizes, both species differ in ploidy level (diploid *C. obtusangula* vs. tetraploid *C. palustris*). This striking fact is made possible by the large genetic distance between these species: the widespread *C. palustris* seems to be more closely related to American than European taxa [70], whereas *C. obtusangula* is an exclusively European and North African species [40]. The species also have substantially different life strategies. *Callitriche palustris* is mostly an annual species with a rapid life cycle [42], [49]; therefore, evolutionary constraints leading to small genome size may play an important role in this species (cf. [114], [115]). On the other hand, *C. obtusangula* is typically perennial, often forming luxuriant vegetative stands. Several other examples of ecologically different congeners are currently known for which genome sizes ratios are incongruent with ploidy levels (e.g. *Chenopodium* [84]; *Anthoxanthum* – Chumová & Trávníček, unpublished data).

The main obstacle to research on water-starworts (and aquatic plants in general) lies in the enormous phenotypic plasticity of these plants and lack of prominent morphological characters. The absence of tools enabling unambiguous determination of particular taxa has resulted in frequent misidentifications and unreliable records. In many such morphologically challenging plant groups, flow cytometry has proven to be the first efficient tool for species and hybrid determination and served as the fundamental method for ensuing studies (e.g. *Chenopodium album* agg. [84]; *Dryopteris carthusiana* agg. [116]; *Fallopia* sect. *Reynoutria* [117]; *Pilosella* [118]). Likewise, easy identification of *Callitriche* species using FCM opens up great opportunities for further interdisciplinary research on this evolutionarily remarkable genus. We highly recommend FCM for taxa delimitation in forthcoming molecular studies on the genus. Genome size can also serve as an independent, species-specific character to define groups in taxonomic research.

Our results will be widely geographically applicable, as well, because many *Callitriche* species occur throughout Europe. Flow cytometry can be applied with equal success to species from Northern and Eastern Europe, where most of the *Callitriche* species represented were included in our study (with the exception of some unclear taxa close to *C. hermaphroditica*, e.g. *C. transvolgensis* in Russia; [40]). Only two species not covered by this study have been reported from north-western Europe: *Callitriche truncata* subsp. *occidentalis* from sect. *Pseudocallitriche* can perhaps be confused only with *C. hermaphroditica* in this region (both species have 2n = 6). *Callitriche brutia*, which is closely related and often indistinguishable from *C. hamulata* ([39], [44]; see comment in Table 1 footnote), has a unique chromosome number (2n = 28, in contrast with 2n = 38 in *C. hamulata*). Because exact genetic delimitation is necessary for further taxonomic assessment of these two problematic taxa, flow cytometry will be able to serve as a basic method for their delimitation.

The situation in the Mediterranean area is more complicated. Additional diploid species (2n = 6–10) are reported from that region, including *C. lusitanica*, *C. pulchra*, *C. truncata* subsp. *truncata* from sect. *Pseudocallitriche* and *C. cribrosa*, *C. lenisulca*, *C. regis-jubae* from sect. *Callitriche*. Our FCM results for *C. lenisulca* provide the foundation for further research on these species. *Callitriche lenisulca*, which is very similar and maybe closely related to *C. cophocarpa* or *C. obtusangula* [107], differs significantly in genome size from both these species. Both *C. lusitanica* and *C. cribrosa* have a different chromosome number (2n = 8) and it is likely that they will be distinct using FCM.

Two similar but significantly different 2C-values were identified in *C. obtusangula*. Italian plants have a smaller genome size than samples from north-western Europe. This differentiation may be associated with the several different karyotypes of this species described by Schotsman [44], [119]. Two karyotypes were reported from France [38], one of which occurs in Atlantic region and the other in Mediterranean region and the Rhine Valley. These two karyotypes were described as somewhat different ecologically, although morphologically indistinguishable. Molecular approaches will be necessary to elucidate their evolutionary origins.

The genome size of the Italian *C. obtusangula* was intermediate between that of Subatlantic specimens of this species and *C. lenisulca*. However, pollen of the Italian C. *obtusangula* was normally developed, which makes it less probable that these plants are F1 hybrids. Additionally, hybridization between these taxa is less likely due to the presence of an effective self-pollination system in *C. lenisulca* ([45]).

### Evolution of polyploid *Callitriche platycarpa*

The origin of polyploid species is currently a widely studied phenomenon. In taxonomically difficult groups that include polyploids, repeated origins of polyploid taxa appears to be the rule rather than the exception [120]–[122]. Especially in aquatic plants, many of which have undergone considerable morphological reduction, the possibility that allopolyploids recognized as single species may actually be polyphyletic cannot be excluded (see, for example, *Ranunculus penicillatus*; [17], [123]). In water-starworts, the tetraploid *C. platycarpa* is believed to be an allotetraploid with the parental species *C. cophocarpa* and *C. stagnalis* (see above). The observed range of genome size for *C. platycarpa* is equal to the sum of these two diploid congeners (see Table 1, Figs 2 and 3), which may support this hypothesis. Anyway, a molecular approach will be necessary to elucidate the evolution of *C. platycarpa*, as autopolyploid origin of some populations cannot be excluded and multiple allopolyploid formation through reciprocal hybridization events is also possible.

### Hybridization

Species-rich genera of aquatic plants may produce extremely high numbers of hybrids (e.g. 99 sufficiently recognized hybrids in *Potamogeton*; [26]). Many aquatic hybrid clones can occupy large areas, produce dominant stands [16], [33], [123], [124], or even exhibit invasive behaviour [125] or extensive introgression [19], [20]. However, the results of this study suggest that hybridization between most Central-European *Callitriche* species is not common, despite the frequent co-occurrence of most taxa (see above). The different pollination biology of particular taxa, high proportions of selfing (geitonogamy) and in some cases also ecological differences between species are presumably the main reasons why water-starworts rarely hybridize. For example, *C. hermaphroditica* and *C. hamulata* are hypohydrogamous (pollinated through wettable exine-reduced pollen under the water surface), whereas the rest of the studied species have pollen with an exine, which is not adapted to spread freely underwater [67], [126]. Some species have highly geitonogamous pollination, realized via contact between male and female flowers (“contacters”: *C. hamulata* [42], *C. lenisulca* [45]) or growth of pollen tubes through filaments and non-floral vegetative tissues (“internal geitonogamy”: *C. palustris* [113]). Therefore, in some cases, even though the stands of different species intermingle at a locality, transfer of pollen to the stigmas of the second species may be physically hardly possible.

The exception to the rule is the triploid hybrid taxon, which we assigned to the F1 hybrid *C. cophocarpa* × *C. platycarpa* (*C.* ×*vigens*). We consider this parental combination the most probable, because triploids were morphologically intermediate between *C. cophocarpa* and *C. platycarpa* (or indistinguishable from one or the other) and were detected almost exclusively in areas of co-occurrence of both species. The genome size of the triploids also best fits the hybrid combination of these two species. The hybrid was found at 25 localities; in some regions (northern and western Bohemia) it seems to be relatively abundant (see Fig. 6).

The hybrid is perennial, forming lush and highly viable vegetative stands, and was found occurring without the presence of parents in the overwhelming majority of localities. Triploid plants were also detected in the Otava River in southern Bohemia, where *C. platycarpa* is not known from the river basin. A similar case is known from Scandinavia [34] where *Callitriche* ×*vigens* is frequent in southernmost Sweden, although one of the parental species (*C. cophocarpa*) is fairly rare there. Occurrence of hybrids in different areas very long after the disappearances of their parents is well documented in *Potamogeton* and *Stuckenia* [16], [35], [37], [127]–[129], and it is probable in *Ranunculus* subsp. *Batrachium* [17], [33].

We cannot yet, however, entirely rule out that some populations of triploids may be of different origin (including hybridization between *C. platycarpa* and *C. stagnalis*, hybridization of diploids *C. stagnalis* and *C. cophocarpa* involving unreduced gametes, or formation of autotriploids of both species). In any case, the combination *C. cophocarpa* × *C. platycarpa* is the most probable, because (i) unreduced gametes are much rarer than reduced gametes; (ii) the putative parental taxa also share pollination systems and ecological preferences, with both often occurring in permanent water bodies, where the newly established non-fertile hybrids can persist. In contrast, *C. stagnalis* prefers to grow in very shallow water or terrestrially, often remains non-flowering in deeper water, and probably possesses a higher rate of geitonogamous pollination [50]. The single plant identified as autotriploid *C. stagnalis* is the very rare exception. This plant was found in a puddle on a forest path, unlike all the other triploids.

The most notable case of hybridization was detected in the Tichá Orlice River. All populations of the hybrid taxon were located in mixed populations with *C. hamulata*, from which they were morphologically indistinguishable without careful inspection of flowers. On the basis of the observed chromosome number (2n = 29), these plants probably represent a cross between *C. hamulata* (2n = 38) and a diploid (2n = 10) or tetraploid (2n = 20) species. The observed genome size (7.60–7.64 pg DNA) can be explained as the hybrid *C. hamulata* × *C. platycarpa* (expected 2C-value 7.40–7.70 pg), *C. hamulata* × unreduced gamete of *C. cophocarpa* (7.48–7.81 pg) or perhaps *C. hamulata* × unreduced gamete of *C. stagnalis* (7.32–7.59 pg). Thus, we hypothesize that this is a rather surprising hybrid between hypohydrogamous, underwater-flowering *C. hamulata*, and a non-hypohydrogamous species with (predominantly) aerial flowers. To date, an analogous case of hybridization has never been observed in angiosperms. The only other *Callitriche* species that has been observed in the Tichá Orlice River is *C. cophocarpa*. In one locality (C061-12), both *C. hamulata* and *C. cophocarpa* co-occurred with the hybrid. Therefore, the parental combination *C. hamulata* (reduced gamete) × *C. cophocarpa* (unreduced gamete) is most likely, but confirmation of this tentative identification by means of molecular markers would be necessary.

Flow cytometry does not enable us to confidently distinguish potential hybrids between homoploid species with similar genome sizes. In the present study, this limitation mainly involves the species *C. stagnalis* and *C. cophocarpa*, which are broadly sympatric in Central Europe. However, there is a clear (although narrow) gap between the genome sizes of the species, without any intermediate values. Many plants with extreme 2C-values and individuals appearing morphologically intermediate were cultivated but no reduced fertility or other indications of hybrid origin were ever observed. Based on these facts, we can exclude hybridization between these species occurring widely in nature. However, this crossing must have occurred at some point, due to the existence of the allopolyploid *C. platycarpa*, which has the same parental species.

### Reliability of published chromosome records and genome sizes

*Callitriche* species have relatively large chromosomes which can be counted relatively easily. Errors arising directly during the counting process are apparently rare in this genus. Mistakes caused by misidentification are much more likely. Fortunately, the monographer and *Callitriche* expert H. D. Schotsman published reliable chromosome counts for most European species in the 1950s and 1960s. These data served as a reference for other researchers. However, confusion can often occur between species with the same chromosome number (in Europe, especially between the species with 2n = 10). Without examining original specimens, it is usually impossible to know whether the published data were based on accurate identification. Chromosome counting combined with genome size determination will allow elimination of most confusion in the future.

The genome sizes obtained by Pijnacker & Schotsman using photometric cytometry with the Feulgen staining method [106] possess somewhat different absolute values and lower accuracy, but the ratio between 2C-values of particular taxa are ± similar to that in our study. The most striking difference is in 2C–values of *C. palustris* and *C. obtusangula*, which appeared to be clearly distinct in the study by Pijnacker & Schotsman but overlapping in our study.

### Geographical distribution of *Callitriche* taxa

Flow cytometry can fundamentally refine our understanding of the distribution of particular taxa (cf. [130], [131]). For example, the taxonomically difficult species *C. platycarpa* has been previously reported from various areas of the Czech Republic but not from the most northern and western parts of the country [132]. In contrast, our study involving flow cytometry detected *C. platycarpa* only in these areas. Therefore, we conclude that the local limit of the distribution of this species passes through this region. In light of our findings, the occurrence of *C. platycarpa* in more eastern parts of Central Europe (e.g., in Slovakia; [133]) seems to be very unlikely.

We also detected *C. hamulata* for Hungary, *C.* ×*vigens* to the Czech Republic and recorded *C. obtusangula* in Denmark, which is its northernmost known occurrence in continental Europe.

*Callitriche hermaphroditica* has been recently referred to as very rare and close to extinction in Central Europe, and probably isolated from its continuous distribution range in Northern Europe [49]. We found this species only in a single pond system in eastern Bohemia (C088-12, C089-12, C090-12). *Callitriche hermaphroditica* is very variable in fruit characters [40], and its intraspecific division needs clarification throughout its extensive distribution range. Based on the fruit size [39], Czech populations clearly belong to subsp. *hermaphroditica*. The additional sample (C127-13) obtained from Sweden was unfortunately sterile and failed to grow in cultivation.

FCM also enables easier identification of introduced taxa. On the west coast of North America, widespread occurrence of plants considered to be *C. hamulata* has recently been reported [40] and this was confirmed using flow cytometry.

### Ecological properties and their consequences

Many *Callitriche* species show relatively broad ecological amplitude and often grow in mixed populations. Despite this, we found that the frequency of occurrence in different biotopes can vary substantially between species. Knowledge of the ecology of water-starworts may facilitate their identification in the field because some species have never been recorded in certain types of habitat in spite of an extensive field survey.

During this study, considerable differences were recorded in the frequency of flowering and fruiting among different species. Whereas mature plants of *C. palustris* almost inevitably produced flowers and fruits in each biotope from which it was recorded, *C. cophocarpa* and *C. platycarpa* remained sterile at most localities (other species were also often sterile, but with lower frequency). Although both *C. cophocarpa* and *C. platycarpa* often grow and form large stands in flowing water, they almost never develop fruits under these circumstances. Both taxa also rarely flower in shaded habitats. For these reasons, species that seldom fruit are highly under-recorded in field surveys. Flow cytometry allows estimation of the true abundances of *Callitriche* species in particular biotopes and also enables identification of mixed populations even in habitats where some species have never been observed to produce fruits or fruit very rarely.

## Conclusions

This study represents the first application of flow cytometry to the genus *Callitriche*. FCM was shown to be the best analytical method for distinguishing *Callitriche* species. This technique also helped increase our knowledge of variation, hybridization, distribution and ecology of particular taxa.

This genus has been considered extremely difficult taxonomically and therefore has been an unpopular subject for research. Nevertheless, the water-starworts are quite intriguing in terms of the evolution of pollination mechanisms and the frequent incidence of polyploidy in different evolutionary lineages [40]. We validated FCM as a powerful tool not only for determining *Callitriche* taxa, but also as a basic method for future multidisciplinary research on the genus. Moreover, applying this method also to other, similarly complex aquatic plant groups should be very promising.

## Supporting Information

Table S1 **Complete list of chromosome numbers published for the genus** ***Callitriche*** **.**
(DOC)

Table S2 **Locality details and genome sizes of 494** ***Callitriche*** **populations from 11 countries, including reference number, number of analysed samples, genome size (three times-measured values are in bold, containing mean and standard deviation), geographic coordinates, altitude, type of habitat (with categories described in Materials and Methods), other** ***Callitriche*** **taxa recorded on the same locality, date of collection and collector name(s) (JanR  =  Jan Rydlo, JarR  =  Jaroslav Rydlo, JP  =  Jan Prančl, KK  =  Klára Kabátová, PT  =  Pavel Trávníček, ZK  =  Zdeněk Kaplan).**
(DOC)
