# Supplementary material for: Genome Size as a Key to Evolutionary Complex Aquatic Plants: Polyploidy and Hybridization in Callitriche (Plantaginaceae)
Source: PLoS One. 2014 Sep 11;9(9):e105997. doi: 10.1371/journal.pone.0105997 (PMC4161354; doi:10.1371/journal.pone.0105997)
Supplement: Table S1 — Complete list of chromosome numbers published for the genus Callitriche . (DOC) [file pone.0105997.s001.doc]

Table S1. Complete list of chromosome numbers published for the genus *Callitriche*

| **Section** | **Taxon** | **Native distribution** | **2n** | **References** | **Notes** |
| --- | --- | --- | --- | --- | --- |
| Pseudo-callitriche | *C. hermaphroditica* L. | boreal Europe and Asia, boreal and temperate areas of North America | 6 | Canada: [1]; Czechoslovakia (Bohemia): [2,3]; Denmark: [4]; England: [5,6]; Greenland: [7]; Poland: [8]; Russia: [9]; Sweden: [10]; USA: [1,11]; unknown origin: [12] (cultivated plants, Munich Botanical Garden), [13] | most of published counts not distinguish between subsp. *hermaphroditica* and subsp. *macrocarpa*(as decribed in [14]); at least Martinsson [10] referred to both subspecies, Jones [5] to subsp. *macrocarpa* and Černohous [2] to subsp. *hermaphroditica* |
|  | *C. truncata* subsp. *occidentalis* (Rouy) Schotsman | coastal areas of W Europe and Mediterranean | 6 | England: [12]; Great Britain: [6]; Portugal: [15]; unknown origin: [16,17] |  |
|  | *C. pulchra* Schotsman | Greece – island of Gavdos, N Libya | 8 | Libya: [18] |  |
|  | *C. lusitanica* Schotsman | Iberian Peninsula, NW Africa | 8 | Morocco: [19]; Portugal: [12,19]; Spain: [19] |  |
| Callitriche | *C. cribrosa* Schotsman | Iberian Peninsula, Italy, NW Africa | 8 | Morocco: [20,21] |  |
|  | *C. stagnalis* Scop. | most of Europe, NW Africa and Macaronesia, probably Middle East | 10 | Belgium: [12]; Canada (naturalized): [22]; Czechoslovakia (Bohemia): [23]; Czech Republic: **this study**; Denmark: [4]; England: [5,24,25,26]; France: [12,24,27]; Germany: [12,24,28,29]; Great Britain: [6,30]; Italy: [12,31,32]; Morocco: [19]; Netherlands: [12,33]; New Zealand (naturalized): [34]; Poland: [35,36]; Portugal: [12,24]; Russia: [37]; Sweden: [38]; Spain: [39] (Baleares), [40]; Switzerland: [12,24]; USA (naturalized): [1]; Wales: [41] | Jørgensen [4] reported also 2n=20, but this number is apparently referred to *C. platycarpa,* which was not recognized by Jørgensen |
|  | *C. regis-jubae* Schotsman | SW of Iberian Peninsula, NW Africa | 10 | Morocco: [42] |  |
|  | *C. cophocarpa* Sendtn. | central, N and E Europe | 10 | Czechoslovakia: [43,44] (Slovakia), [23] (Bohemia), [45] (Moravia); Czech Republic: **this study**; Denmark: [6]; France: [46]; Germany: [12,28,29,47]; Poland: [35,36]; Slovakia: [48]; Sweden: [38,49]; Switzerland: [12,27,47]; Yugoslavia: [12,47] | other published chromosome numbers are doubtful (2n=12 for Sweden [50]; both 2n=10 and 2n=12 for Slovakia [43]) |
|  | *C. lenisulca* Clavaud | European & Asian Mediterranean | 10 | France: [51] |  |
|  | *C. mathezii* Schotsman | Morocco | 10 | Morocco: [52] |  |
|  | *C. obtusangula* Le Gall | W & S Europe, NW Africa | 10 | England: [5,12,24,26]; France: [12,24]; Germany: [12,24,29]; Great Britain: [6]; Italy: [12]; Morocco: [19]; Netherlands: [12,24,33], **this study**; Portugal: [12,24]; Tunisia: [19]; Wales: [41] |  |
|  | *C. favargeri* Schotsman | Ethiopia | 10 | Ethiopia: [53] |  |
|  | *C. hedbergiorum* Schotsman | Ethiopia | 10 | Ethiopia: [54] |  |
|  |  |  | 20 | Ethiopia: [55] |  |
|  | *C. deflexa* A.Braun | Mexico to Argentina | 10 | Morocco (introduced): [19]; Portugal (introduced): [15,19] |  |
|  | *C. peploides* Nutt. | SE USA, Cuba, Central America | 10 | USA: [1] |  |
|  | *C. terrestris* Raf. | E & SE USA | 10 | USA: [1] |  |
|  | *C. muelleri* Sond. | E Australia, New Zealand, Kermadec Islands | 10 | New Zealand: [34] |  |
|  | *C. ×vigens* K. Martinsson [*C. cophocarpa × platycarpa*] | N & central Europe | 15 | Czech Republic: **this study**; Denmark: [4,56]; France: [12,46,47]; Germany: [27,29,57,58]; Sweden: [38,59]; Switzerland: [12,27,47] | Winge [60] erroneously reported 2n=16 from the same locality as Jørgensen [4] |
|  | *"C. stagnalis"* |  | 15 | New Zealand: [34] | no additional informations |
|  | *C. vulcanicola* Schotsman | central tropical Africa | 18 | Kenya: [55] |  |
|  | *C. platycarpa* Kütz. | Atlantic Europe | 20 | Belgium: [12]; Czech Republic: **this study**; England: [12,26]; France: [12,27,46]; Germany: [12,27,28,29]; Great Britain: [6,30]; Netherlands: [12,33]; Poland: [36]; Spain: [12,40]; Sweden: [38]; Switzerland: [12,47]; Wales: [41] |  |
|  | *C. palustris* L. | Europe (predominantly central, N and E), Asia & North America (predominantly boreal and temperate) | 20 | Canada: [1,61]; Czech Republic: **this study**; Denmark: [4]; France: [62]; Germany: [12,29]; Netherlands: [12,33]; Poland: [8]; Russia: [9,37,63,64,65,66], [67] (Chukotka); Spain: [40,62]; Switzerland: [12]; USA: [1,11] |  |
|  | *C. anceps* Fernald | Greenland, Alaska, Canada, USA | 20 | Greenland: [68]; USA: [49] | taxonomically unclear species (cf. [69,70]) |
|  | *C. subanceps* Petrov | E Russia | 20 | Russia (Chukotka): [71] | taxonomically unclear species |
|  | *C. heterophylla* Pursh var. *heterophylla* | Nova Scotia, USA, Mexico, Guatemala, Hispaniola | 20 | Canada: [72]; USA: (this variety?) | the record 2n=10 for USA ([11], variety not specified) is doubtful |
|  |  |  | 40 | USA: [1] |  |
|  | *C. heterophylla* var. *bolanderi* (Hegelm.) Fassett | British Columbia to S California | 20 | Canada: [1,72]; USA: [1] |  |
|  | *C. marginata* Torr. | British Columbia to Gulf of California | 20 | Canada: [1]; USA: [1] |  |
|  | *C. nuttallii* Torr. | USA – Mexican Gulf area | 20 | USA: [1] |  |
|  | *C. petriei* R. Mason subsp. *petriei* | New Zealand | 20 | New Zealand: [34] |  |
|  |  |  | 30 | New Zealand: [34] | 2 records, in Dawson [73] reported as *C.* "aff. *petriei*" |
|  | *C. petriei* subsp. *chathamensis* R. Mason | Chatham Islands | 20 | New Zealand (Chatham Islands): [34] |  |
|  | *C. brutia* Petagna | W, NW and SW Europe, NW Africa, Middle East (?) | 28 | France: [12,19]; Germany: [29]; Morocco: [19]; Portugal: [15,19]; Spain: [40,74], [39] (Baleares); Wales: [26,41,75] | the number 2n=20 for Iceland [76] is doubtful |
|  | *C.* *hamulata × platycarpa*? |  | 29 | Sweden: [59] | the single population; parental species of this hybrid were not reliably proved |
|  | *C.* *hamulata × cophocarpa*? |  | 29 | Czech Republic: **this study** |  |
|  | *C. hamulata* Kütz. ex W.D.J. Koch | W, N & central Europe, Greenland | 38 | Czechoslovakia (Bohemia): [45,78]; Czech Republic: **this study**; Denmark: [4]; England: [5,77]; France: [12,27,47,79]; Germany: [12,27,29,47]; Great Britain: [6,26]; Iceland: [76]; Netherlands: [12,33]; Poland: [35]; Spain: [40]; Switzerland: [12,47]; Wales: [12,41,47] | the records of 2n=40 for Iceland [50,76] are doubtful; Wołek [80] states that he counted the material from S Poland, but without indicating the chromosome number |
|  | *C. trochlearis* Fassett | Oregon, California | 40 | USA: [1] |  |
|  | *C. antarctica* Engelm. ex Hegelm. | Tasmania, Tierra del Fuego, Falklands & other subantarctic islands | 40 | Australia (Macquarie Island): [81]; Falkland Islands: [82]; New Zealand (Campbell Island): [34] | [81] – meiosis (n=20) |
|  | *C. aucklandica* R.Mason | Auckland Islands | 40 | New Zealand (Auckland Islands): [34] |  |

The chromosome counts published by Villani et al. [83] from Italy were not included, because the plant determination was incorrect and based on inappropriate methodology. In light of these shortcomings, the doubtful count of *C. platycarpa* by Serra et al. [84] was also omitted, having the same authors as [83].

*Callitriche truncata* Guss. subsp. *truncata*, *C. truncata* subsp. *fimbriata* Schotsman and *C. transvolgensis* Tzvelev are the only European taxa with unknown chromosome numbers. Although Lansdown [85] provides chromosome numbers for these taxa (2n=6 for *C. truncata* subsp. *truncata*, 2n=10 for *C. truncata* subsp. *fimbriata* and *C. transvolgensis*), these numbers were reported in error (R.V. Lansdown, pers. comm. 2014). Martinsson [10] also states 2n=6 for *C. truncata* subsp. *truncata*, but does not list any sources. Actually, chromosome counts of these taxa probably have never been reliably published.

References to Table S1:

1. Philbrick CT (1994) Chromosome counts for *Callitriche* (Callitrichaceae) in North America. Rhodora 96: 383–386.
2. Černohous F (1980) *Callitriche hermaphroditica* v Československu [*Callitriche hermaphroditica* in Czechoslovakia]. Preslia 52: 203–208.
3. Löve Á, editor (1982a) Chromosome number reports LXXVI. Taxon 31: 574–598
4. Jørgensen CA (1923) Studies on Callitrichaceae. Bot Tidsskr 38: 81–126.
5. Jones H (1955) Notes on the identification of some British species of *Callitriche*. Watsonia 3: 186–192.
6. Savidge JP (1958) A cyto-taxonomical investigation of species of *Callitriche* occurring in north-west Europe. PhD Thesis, University of Liverpool, England.
7. Dalgaard V (1988) Chromosome numbers in some vascular plants from the Disko Bugt area (west Greenland). Willdenowia 18: 243–252.
8. Skalińska M, Jankun A, Wcisło, H, editors (1971) Studies in chromosome numbers of Polish Angiosperms. Eighth contribution. Acta Biol Cracov, Ser Bot 14: 55–102.
9. Lavrenko AN, Serditov NP, Ulle ZG (1991) Chisla khromosom nekotorykh vidov sosudistykh rastenii Pechoro-Ilychskogo zapovednika (Komi USSR) [Chromosome numbers in some species of vascular plants from the Pechoro-Ilychsky Reservation (Komi ASSR)]. Bot Zhurn (Moscow & Leningrad) 76: 473–476.
10. Martinsson K (1991) Geographical variation in fruit morphology in Swedish *Callitriche hermaphroditica* (Callitrichaceae). Nord J Bot 11: 497–512.
11. Milek JA, Pratt GA (1968) Cytological studies of three species in the genus *Callitriche*. J Colorado-Wyoming Acad Sci 6: 52.
12. Schotsman HD (1967) Les Callitriches. Espèces de France et taxa nouveaux d’Europe. In: Jovet P, editor. Flora de France, vol. 1. Paris: Editions Paul Lechevalier, pp. 1–151.
13. Wagenaar EB (1969 End-to-end chromosome attachments in mitotic interphase and their possible significance to meiotic chromosome pairing. Chromosoma 26: 410–426.
14. Lansdown RV (2006) Notes on the water-starworts (*Callitriche*) recorded in Europe. Watsonia 26: 105–120.
15. Schotsman HD (1961) Notes on some Portuguese species of *Callitriche*. Bol Soc Broteriana 35: 95–127.
16. Darlington CD, Janaki Ammal EK (1945) Chromosome atlas of cultivated plants. London: G. Allen & Unwin Ltd. 397 p.
17. Darlington CD, Wylie AP (1956) Chromosome atlas of flowering plants. New York: Macmillian. 519 p.
18. Schotsman HD (1969) Contribution a l’etude des Callitriches du bassin Mediterraneen et du Maroc. Le nombre chromosomique de *Callitriche pulchra* Schotsm. Bull Cent Etud Rech Sci, Biarritz, 7: 869–872.
19. Schotsman HD (1977) Callitriches de la région Méditerranéenne. Nouvelles observations. Bull Cent Etud Rech Sci, Biarritz, 11: 241–312.
20. Schotsman HD (1968) Nombre chromosomique de *Callitriche cribrosa* Schotsm. Bull Cent Etud Rech Sci, Biarritz, 7: 87–88.
21. Schotsman HD (1971) Etudes sur les Callitriches du Maroc. I. Quelques nouvelles observations sur *Callitriche cribrosa* Schotsm. Bull Soc Sci Nat Phys Maroc 51: 157–166.
22. Gervais C, Parent M, Trahan R, Plante S (1997) Callitrichaceae. In: Stace CA, editor. IOPB chromosome data 12. Newslett Int Organ Pl Biosyst (Oslo) 28: 17.
23. Prančl J (2013) Rod *Callitriche* (hvězdoš) v České republice. II. *C. cophocarpa*, *C. stagnalis*, *C. platycarpa*, *C.* × *vigens* [The genus *Callitriche* (water-starwort) in the Czech Republic. II. *C. cophocarpa*, *C. stagnalis*, *C. platycarpa*, *C.* × *vigens*]. Zprávy Čes Bot Společ 48: 179–262.
24. Schotsman HD (1961) Races chromosomiques chez *Callitriche stagnalis* Scop. et *Callitriche obtusangula* Legall. Ber Schweiz Bot Ges 71: 5–17.
25. Montgomery L, Khalaf M, Bailey JP, Gornall RJ (1997) Contributions to a cytological catalogue of the British and Irish flora, 5. Watsonia 21: 365–368.
26. Demars BOL, Gornall RJ (2003) Identification of British species of *Callitriche* by means of isozymes. Watsonia 24: 389–399.
27. Gregor T, Hand R (2009) Chromosomenzahlen von Farn- und Samenpflanzen aus Deutschland 4. Kochia 4: 37–46.
28. Dersch G (1965) Notizen über das Vorkommen von *Callitriche*-Arten in (Nord-)Hessen. Hess Flor Briefe 14: 35–44.
29. Dersch G (1986) Zur Verbreitung der *Callitriche*-Arten (Wassersterne) in Niedersachsen. Gött Flor Rundbr 20: 79–100.
30. Savidge JP. 1956. *Callitriche platycarpa* Kütz. Proc Bot Soc British Isles 2: 2.
31. Ficini G, Garbari F, Giordani A, Tomei PE. 1980. Numeri cromosomici per la Flora Italiana: 683–689. Inform Bot Ital 12: 113–116.
32. Gomarasca S, Cicatelli A, Maggioni LA, Castiglione S (2012) The use of *Callitriche* for water quality determination can be misleading: An example from the Po Valley in Northern Italy. Pl Biosystems 147: 459–471.
33. Schotsman HD (1954) A taxonomic spectrum of the section *Eu-Callitriche* in the Netherlands. Acta Bot Neerl 3: 313–384.
34. Beuzenberg EJ, Hair JB (1963) Contributions to a chromosome atlas of the New Zealand flora – 5. Miscellaneous families. New Zealand J Bot 1: 53–67.
35. Skalińska M, Pogan E, Jankun A, editors (1968) Dalsze badania nad kariologia flory polskiej Cz. VII. [Further studies in chromosome numbers of Polish Angiosperms. Seventh contribution]. Acta Biol Cracov, Ser Bot 11: 199–224.
36. Bączkiewicz A, Szoszkiewicz K, Cichocka, J, Celińsky K, Drapikowska M, et al. (2007) Isozyme patterns of *Callitriche cophocarpa*, *C. stagnalis* and *C. platycarpa* from 13 Polish rivers. Biol Lett 44: 103–114.
37. Sokolovskaya AP (1932 K sistematike i kariologii roda *Callitriche* [On the systematics and karyology of the genus *Callitriche*]. Trudy Petergofsk Est-Nauchnogo Instit 8: 150–172.
38. Martinsson K (1985) Problem kring *Callitriche platycarpa*, plattlånke [Problems concerning *Callitriche platycarpa*, various-leaved Water-starwort]. Svensk Bot Tidskr 79: 165–174.
39. Schotsman HD, Andreas CH (1980) Callitriches de la région Méditerranéenne. III. – Observations personelles et nouvelles remarques sur les espèces des Iles Baléares (Menorca et Mallorca). Bull Cent Etud Rech Sci, Biarritz, 13: 77–88.
40. Fernández Bernaldo de Quirós C (1987) Números cromosomáticos de algunas especies acuáticas de *Ranunculus* L. y *Callitriche* en Asturias. Revista Biol Univ Oviedo 5: 65–70.
41. Lewis-Jones LJ, Kay QON (1977) The cytotaxonomy and distribution of water starworts (*Callitriche* spp) in West Glamorgan. Nature in Wales 15: 180–183.
42. Schotsman HD (1973) Note sur *Callitriche regis-jubae* nov. spec. Espece nouvelle du bassin mediterraneen occidental. Bull Soc Hist Nat Afr Nord 64: 25–37.
43. Májovský J, editor (1978) Index of chromosome numbers of Slovakian flora (Part 6). Acta Fac Rerum Nat Univ Comen, Ser Bot 26: 1–42.
44. Löve Á, editor (1978) In IOPB chromosome number reports. LXI. Taxon 27: 375–392.
45. Měsíček J, Jarolímová V (1992) List of chromosome numbers of the Czech vascular plants. Praha: Academia. 144 p.
46. Schotsman HD, Haldimann G (1981) Callitriches inédites du Jura français *C. cophocarpa* Sendtn., *C. platycarpa* Kütz. (Angiospermae) et l’hybride dans la partie septentrionale. Bull Soc Neuchâteuloise Sci Nat 104: 131–143.
47. Schotsman HD (1961) Contribution a l’étude des *Callitriche* du canton de Neuchatel. Bull Soc Neuchâteuloise Sci Nat 84: 89–101.
48. Májovský J, Uhríková A, Javorčíková D, Mičieta K, Králik E, et al. (2000) Prvý doplnok karyotaxonomického prehľadu flóry Slovenska [First addition to the karyotaxonomical overview of the flora of Slovakia]. Acta Fac Rerum Nat Univ Comen, Ser Bot, Suppl. 1: 1–127.
49. Löve Á, editor (1975) IOPB chromosome number reports L. Taxon 24: 671–678.
50. Löve Á, Löve D (1948) Chromosome numbers of Northern plant species. Reykjavík: Ingolfsprent. 131 p.
51. Schotsman HD, Andreas CH (1974) *Callitriche lenisulca* Clav. espèce méconnue. Bull Cent Etud Rech Sci, Biarritz, 10: 285–316.
52. Schotsman HD (1982) Le nombre chromosomique de *Callitriche mathezii* Schotsm. Bull Cent Etud Rech Sci, Biarritz, 14: 235–238.
53. Schotsman HD (1984) Les Callitriches d’Afrique intertropicale continentale. I. Une espèce nouvelle d’Ethiopie: *Callitriche favargeri* Schotsman, sp. nova. Bot Helv 94: 285–294.
54. Schotsman HD (1988) Les *Callitriche* L. de l’Afrique intertropicale continentale III. Historique et description de trois nouvelles espèces. Bull Mus Natl Hist Nat, Paris, B, Adansonia 10: 3–18.
55. Hedberg O, Hedberg I (2001) Tropical African *Callitriche* (Callitrichaceae): A neglected and taxonomically difficult part of an evolutionary extremely interesting family. Biol Skr 54: 19–30.
56. Savidge JP (1959) An interspecific hybrid in European *Callitriche*. Proc Bot Soc British Isles 3: 335.
57. Dersch G (1974) Über einige Chromosomenzählungen an mitteleuropäischen Blütenpflanzen. II. Philippia 2: 75–82.
58. Gregor T (2010) 1670. *Callitriche* ×*vigens* (= *Callitriche cophocarpa* × *platycarpa*). In: Fundmeldungen. Neufunde – Bestätigungen – Verluste. Bot Naturschutz Hessen 23: 120.
59. Martinsson K (1991) Natural hybridization within the genus *Callitriche* (Callitrichaceae) in Sweden. Nord J Bot 11: 143–151.
60. Winge Ø (1917) Studier over planterigets chromosomtal og chromosomernes betydning. Medd Carisb Lab 13: 127–267.
61. Löve Á, editor (1982) Chromosome number reports LXXVII. Taxon 31: 761–777.
62. Haldimann G (1989) Agamospermie chez la forme submergée de *Callitriche palustris* L. et contribution à l’étude cytologique de cette espèce. – Bull Soc Neuchâteuloise Sci Nat 112: 29–32.
63. Krogulevich RE (1978) Kariologicheskiy analiz vidov flory Vostochnogo Sayana [Karyological analysis of the species of the flora of the Eastern Sayan Mts.]. In: Malyshev LI, Peshkova GA, editors. Flora Pribajkaľya [Flora of the Pribaikalye]. Novosibirsk: Nauka, pp. 19–48.
64. Probatova NS, Rudyka EG, Shatalova SA (2001) Chisla khromosom nekotorykh vidov flory okrestnostei Vladivostok (Primorskii krai) [Chromosome numbers in some plant species from the environs of Vladivostok City (Primorsky region)]. Bot Zhurn 86: 168–172.
65. Marhold K, editor (2010) IAPT/IOPB chromosome data 9. Taxon 59: 1298–1302, E1–E15.
66. Marhold K, editor (2011) IAPT/IOPB chromosome data 12. Taxon 60: 1784–1796, E1–E72.
67. Zhukova PG (1982) Chisla khromosom nekotorykh vidov rasteniy severo-vostoka Azii [Chromosome numbers of some plant species of northern-eastern Asia]. Bot Zhurn SSSR 67: 360–365
68. Dalgaard V (1989) Additional chromosome numbers in vascular plants from the Disko Bugt area (West Greenland). Willdenowia 19: 199–213.
69. Fassett NC (1951) *Callitriche* in the New World. Rhodora 53: 137–155, 161–182, 185–194, 209–222.
70. Lansdown RV (2009) Nomenclatural notes on *Callitriche* (Callitrichaceae) in North America. *Novon* 19: 364–369.
71. Zhukova PG, Petrovsky VV (1975 Khromosomnye chisla nekotorykh vidov rastenii zapadnoi Chukotki [Chromosome number of some Western Chukotka plant species]. Bot Zhurn (Moscow & Leningrad) 60: 395–401.
72. Taylor RL, Mulligan FA (1968) Flora of the Queen Charlotte Islands. Part 2. Cytological aspects of the vascular plants. Ottawa: Queen’s Printers. 148 p.
73. Dawson MI (2000) Index of chromosome numbers of indigenous New Zealand spermatophytes. New Zealand J Bot 38: 47–150.
74. Löve Á, Kjellqvist E (1974) Cytotaxonomy of Spanish plants. IV. Dicotyledons: Caesalpiniaceae – Asteraceae. Lagascalia 4: 153–211.
75. Wentworth JE, Bailey JP, Gornall RJ (1991) Contributions to a cytological catalogue of the British and Irish flora, 1. Watsonia 18: 415–417.
76. Löve Á, Löve D (1956) Cytotaxonomical conspectus of the Icelandic flora. Acta Horti Gotob 20: 65–290.
77. David RW (1958) An introduction to the British species of *Callitriche*. Proc Bot Soc British Isles 3: 28–32.
78. Prančl J (2012) Rod *Callitriche* (hvězdoš) v České republice. I. Úvod a určování, druhy *C. hermaphroditica*, *C. hamulata* a *C. palustris* [The genus *Callitriche* (water-starwort) in the Czech Republic. I. Introduction and determination, the species *C. hermaphroditica*, *C. hamulata* and *C. palustris*]. Zprávy Čes Bot Společ 47: 209–290.
79. Haldimann G (1982) Contribution a l’étude cytologique de *Callitriche hamulata* Kutz. (Angiospermae). Bull Soc Neuchâteuloise Sci Nat 105: 59–63.
80. Wołek J (1971) Rozmieszczenie roślin wodnych w dolinie Dunajca na przedpolu Pienińskiego Parku Narodovego [Distribution of the aquatic plants in the Dunajec River valley in the Foreland of the Pieniny National Park]. Fragm Florist Geobot 17: 237–250.
81. Moore DM (1960) Chromosome numbers of flowering plants from Macquarie Island. Bot Notiser 113: 185–191.
82. Moore DM (1967) Chromosome numbers of Falkland Islands Angiosperms. Br Antarct Surv Bull 14: 69–82.
83. Villani M, Marcurri R, Bassan E, Magrin M, Tornadore N (2004) The genus *Callitriche* in northeastern Italy: First systematic contribution. Israel J Pl Sci 52: 331–340.
84. Serra C, Valdés B, Marcucci R, Tornadore N (2001) Reports (1254–1263). In: Kamari G, Blanché C, Garbari F, editors (2001) Mediterranean chromosome number reports – 11. Fl Medit 11: 466–473.
85. Lansdown RV (2008) Water-starworts (*Callitriche*) of Europe. B.S.B.I. Handbook no. 11. London: Botanical Society of the British Isles. 184 p.
