# Supplementary material for: Genome Size as a Key to Evolutionary Complex Aquatic Plants: Polyploidy and Hybridization in Callitriche (Plantaginaceae)
Source: PLoS One. 2014 Sep 11;9(9):e105997. doi: 10.1371/journal.pone.0105997 (PMC4161354; doi:10.1371/journal.pone.0105997)
Supplement: Table S2 — Locality details and genome sizes of 494 Callitriche populations from 11 countries, including reference number, number of analysed samples, genome size (three times-measured values are in bold, containing mean and standard deviation), geographic coordinates, altitude, type of habitat (with categories described in Materials and Methods), other Callitriche taxa recorded on the same locality, date of collection and collector name(s) (JanR = Jan Rydlo, JarR = Jaroslav Rydlo, JP = Jan Prančl, KK = Klára Kabátová, PT = Pavel Trávníček, ZK = Zdeněk Kaplan). (DOC) [file pone.0105997.s002.doc]

Table S1: Locality details for 1076 *Callitriche* individuals from 494 localities, including reference number, number of analysed samples, genome size (three times-measured values are in bold, containing mean and standard deviation), geographic coordinates, altitude, type of habitat (with categories described in Materials and Methods), other *Callitriche* taxa recorded on the same locality, date of collection and collector name(s) (JanR = Jan Rydlo, JarR = Jaroslav Rydlo, JP = Jan Prančl, KK = Klára Kabátová, PT = Pavel Trávníček, ZK = Zdeněk Kaplan).

| **Reference no.** | **No. of indivi-duals** | **2C-value (pg) + s.d.** | **Locality** | **Coordinates (WGS 84)** | **Altitude (m)** | **Type of habitat** | **Other taxa** | **Date + Collector(s)** |
| --- | --- | --- | --- | --- | --- | --- | --- | --- |
| ***C. cophocarpa* Sendtn.** | | | | | | | | |
|  |  |  |  |  |  |  |  |  |
| C007-07 | 1 | 3.27 | Czech Republic, distr. Tábor, Hamr, ditch of Vřesenský stream below overflow of Hluboký u Hamru fishpond | 49°9'37"N, 14°46'15"E | 425 | ditch |  | 28 June 2007 JP & ZK |
| C009-07a | 2 | 3.18 | Czech Republic, distr. Klatovy, Sušice, Roušarka stream on S edge of town | 49°13'12.9"N, 13°30'33.5"E | 480 | stream (sand) | *C. hamulata* | 29 June 2007 JP |
| C010-07 | 1 | 3.27 | Czech Republic, distr. Nymburk, Libice nad Cidlinou, Libický luh forest, Bačovka stream | 50°6'16"N, 15°10'9"E | 185 | stream (mud) |  | 6 July 2007 JP |
| C015-07 | 1 | 3.20 | Czech Republic, distr. Mělník, Kly, pool near right bank of Labe River | 50°18'31.8"N, 14°29'47.1"E | 160 | exposed bottom |  | 8 Aug. 2007 JP |
| C016-07 | 1 | 3.22 | Czech Republic, distr. Mělník, Kly, Tuhaňská strouha canal in village | 50°18'28.9"N, 14°30'8.1"E | 160 | stream (mud) |  | 8 Aug. 2007 JP |
| C028-07 | 1 | 3.10 | Czech Republic, distr. Nymburk, Loučeň, Knížecí fishpond | 50°17'28.7"N, 15°0'28.7"E | 245 | exposed bottom |  | 22 Aug. 2007 JP |
| C029-07 | 1 | 3.18 | Czech Republic, distr. Nymburk, Loučeň, rivulet below small pond by spring Dobrá Voda | 50°18'5"N, 15°1'48"E | 250 | stream (mud) |  | 22 Aug. 2007 JP |
| C035-07 | 1 | 3.21 | Czech Republic, distr. Jindřichův Hradec, Třeboň, Prostřední Stoka canal near railway station Třeboň lázně | 49°0'24.0"N, 14°46'36.2"E | 420 | stream (mud) |  | 28 June 2007 JP |
| C001-08a | 3 | 3.25 | Czech Republic, Praha, city distr. Modřany, pools "Modřanské laguny" on right bank of Vltava River | 49°59'48.3"N, 14°24'11.1"E | 190 | pool | *C. hamulata* | 1 May 2008 JP |
| C002-08 | 3 | **3.25±0.03** | Czech Republic, Praha, city distr. Běchovice, Běchovický stream | 50°5'1.0"N, 14°37'30.1"E | 235 | stream (mud) |  | 8 May 2008 JP |
| C003-08 | 6 | **3.21±0.03** | Czech Republic, Praha, city distr. Běchovice, pool in littoral zone of Počernický fishpond | 50°4'59.3"N, 14°35'46.9"E | 230 | pool |  | 8 May 2008 JP |
| C004-08 | 4 | 3.19 | Czech Republic, Praha, city distr. Zbraslav, locality Krňák, Lipanský stream | 49°58'23"N, 14°22'29"E | 195 | stream (mud) | *C. stagnalis* | 15 May 2008 JP |
| C006-08a | 2 | 3.22 | Czech Republic, distr. Chrudim, Rabštejnská Lhota, small pond on Podhůra stream | 49°54'28.5"N, 15°46'31.6"E | 315 | fishpond | *C. hamulata* | 23 May 2008 JP |
| C009-08 | 1 | 3.18 | Czech Republic, distr. Chrudim, Nasavrky, small pond in locality V Koutech | 49°50'6.6"N, 15°47'40.5"E | 530 | pool |  | 24 May 2008 JP |
| C010-08 | 1 | **3.18±0.01** | Czech Republic, distr. Chrudim, Trhová Kamenice, settlement Petrkov, forest fishpond | 49°48'4.0"N, 15°50'35.4"E | 585 | fishpond |  | 24 May 2008 JP |
| C015-08 | 1 | 3.20 | Czech Republic, distr. Chrudim, Jeníkov, ruts on forest path near solitude Lány | 49°43'39.2"N, 15°56'42.1"E | 610 | puddle |  | 25 May 2008 JP |
| C016-08 | 2 | **3.18±0.03** | Czech Republic, distr. Praha-východ, Káraný, locality Lipovka – Grado, ditch | 50°10'45.8"N, 14°46'2.0"E | 175 | ditch |  | 30 May 2008 JP |
| C017-08b | 2 | 3.24 | Czech Republic, distr. Břeclav, ruts on forest path NE of chateau Pohansko | 48°44'12"N, 16°54'20"E | 155 | puddle | *C. palustris* | 11 June 2008 JP |
| C018-18 | 1 | 3.28 | Czech Republic, distr. Břeclav, ditch NE of chateau Pohansko | 48°44'8"N, 16°54'11"E | 150 | ditch |  | 11 June 2008 JP |
| C019-08 | 1 | 3.26 | Czech Republic, distr. Břeclav, pool Bornova jama near chateau Lány | 48°42'56"N, 16°55'7"E | 150 | pool |  | 11 June 2008 JP |
| C021-08 | 1 | 3.25 | Czech Republic, distr. Břeclav, ditch SSE of chateau Lány | 48°40'57.3"N, 16°55'34.1"E | 150 | ditch |  | 11 June 2008 JP |
| C0234-08 | 1 | **3.22±0.03** | Czech Republic, distr. Břeclav, Kostice, Stibůrkovská jezera area, complex of pools | 48°44'41"N, 16°59'54"E | 145 | pool |  | 12 June 2008 JP |
| C025-08 | 1 | **3.22±0.01** | Czech Republic, distr. Klatovy, Horská Kvilda, tributary of Hamerský stream W of village | 49°3'22.6"N, 13°32'23.6"E | 1010 | stream (sand) |  | 2 July 2008 I. Prančlová |
| C030-08 | 1 | 3.19 | Czech Republic, distr. Jindřichův Hradec, Lutová, ditch along bank of Velký Holý fishpond | 48°58'36.0"N, 14°52'42.4"E | 435 | ditch |  | 10 July 2008 JP |
| C037-08 | 4 | **3.25±0.03** | Czech Republic, distr. Jindřichův Hradec, Lužnice, ditch under railway bridge | 49°4'0.2"N, 14°44'9.6"E | 425 | ditch |  | 12 July 2008 JP |
| C047-08 | 1 | 3.26 | Czech Republic, distr. Olomouc, Mladeč, pool in floodplain forest N of village | 49°42'46"N, 17°1'22.E | 240 | pool |  | 23 July 2008 JP |
| C061-08 | 1 | 3.17 | Czech Republic, distr. Cheb, Nová Ves, pool in kaolin quarry SSW of village | 50°9'42"N, 12°24'42"E | 440 | pool | *C. hamulata* | 3 Aug. 2008 JP |
| C070-08 | 2 | 3.19 | Czech Republic, distr. Prachatice, Volary, Volarský stream near bus station | 48°54'34"N, 13°53'30"E | 755 | stream (mud) |  | 6 Sept. 2008 JP |
| C071-08 | 6 | 3.19 | Czech Republic, distr. Prachatice, Volary, Volarský stream under railway bridge | 48°54'24.3"N, 13°53'42.1"E | 750 | stream (mud) |  | 6 Sept. 2008 JP |
| C072-08 | 1 | 3.17 | Czech Republic, distr. Prachatice, Volary, Planerův Dvůr settlement, small meadow reservoir | 48°53'6"N, 13°54'9"E | 760 | pool |  | 6 Sept. 2008 JP |
| C076-08 | 2 | **3.16±0.03** | Czech Republic, distr. Prachatice, Volary, lower fishpond on Jedlový stream in Stögrova Huť settlement | 48°54'52.4"N, 13°51'19.1"E | 795 | fishpond | *C. palustris* | 6 Sept. 2008 JP |
| C080-08 | 1 | 3.18 | Czech Republic, distr. Prachatice, Stožec, ditch along forest path SE of Na vrchu hill | 48°50'56"N, 13°50'31"E | 855 | puddle |  | 7 Sept. 2008 JP |
| C081-08 | 1 | 3.16 | Czech Republic, distr. Prachatice, Stožec, former bed of Schwarzenberský canal near crossing with Jelení stream | 48°49'33"N, 13°50'51"E | 910 | puddle |  | 7 Sept. 2008 JP |
| C083-08 | 1 | 3.17 | Czech Republic, distr. Prachatice, Jelení, puddle near bank of Hučina stream | 48°49'42"N, 13°51'50"E | 805 | puddle |  | 7 Sept. 2008 JP |
| C084-08 | 2 | 3.24 | Czech Republic, distr. Prachatice, Jelení, Jelení jezírko reservoir | 48°49'15.2"N, 13°53'32.2"E | 945 | reservoir |  | 7 Sept. 2008 JP |
| C086-08 | 1 | 3.25 | Czech Republic, distr. Žďár nad Sázavou, Radostín, stream flowing from peat-bog Radostínské rašeliniště | 49°39'6"N, 15°53'14.2"E | 620 | stream (mud) |  | 22 Sept. 2008 JP |
| C089-08 | 2 | 3.21 | Czech Republic, distr. Žďár nad Sázavou, Karlov, ditch SW of village, near estuary to Velké Dářko fishpond | 49°38'35"N, 15°54'7"E | 615 | ditch |  | 22 Sept. 2008 JP |
| C092-08 | 1 | 3.24 | Czech Republic, distr. Příbram, Voznice, Voznický stream under bridge of highway R4 | 49°49'0.6"N, 14°13'18.6"E | 360 | stream (mud) |  | 6 Nov. 2008 JP |
| C093-08 | 2 | 3.18 | Czech Republic, distr. Příbram, Voznice, Horní fishpond (Horní Tušiny), tall-segde vegetation in littoral zone | 49°48'25"N, 14°12'46"E | 400 | exposed bottom |  | 6 Nov. 2008 JP |
| C094-08 | 3 | 3.22 | Czech Republic, distr. Příbram, Voznice, Charvátův fishpond | 49°48'56.1"N, 14°11'34.3"E | 415 | exposed bottom |  | 6 Nov. 2008 JP |
| C096-08 | 1 | 3.20 | Czech Republic, distr. Příbram, Chouzavá, ruts on forest path W of hill Točná | 49°50'12"N, 14°11'42"E | 420 | puddle | *C. stagnalis* | 6 Nov. 2008 JP |
| C101-08 | 1 | **3.21±0.04** | Czech Republic, distr. Prachatice, Stožec, puddle on forest path ESE of village | 48°51'22"N, 13°50'2"E | 850 | puddle |  | 7 Sept. 2008 JP |
| C102-08 | 1 | 3.16 | Czech Republic, distr. Česká Lípa, Hradčany, Hradčanské rybníky area, Hradčanský stream below Černý fishpond | 50°36'29.1"N, 14°45'38.6"E | 279 | stream (sand) |  | 17 Sept. 2008 J. Sádlo & P. Petřík |
| C103-08 | 1 | 3.24 | Czech Republic, distr. Hradec Králové, Běleč nad Orlicí, pool on left bank of Orlice River | 50°11'54.0"N, 15°57'54.7"E | 236 | pool |  | 25 Sept. 2008 ZK |
| C002-09 | 3 | 3.22 | Czech Republic, distr. Litoměřice, Písty, pool on W edge of village | 50°25'30"N, 14°8'45"E | 160 | pool |  | 25 Apr. 2009 JP |
| C003-09 | 2 | **3.22±0.03** | Czech Republic, distr. Klatovy, Rejštejn, former side bed of Losenice stream near Václavíkova sawmill | 49°8'3"N, 13°32'14"E | 605 | pool |  | 30 Apr. 2009 JP |
| C005-09 | 2 | 3.19 | Czech Republic, distr. Mělník, Jelenice, forest marsh near locality Polabská černava | 50°20'26"N, 14°32'16"E | 185 | stream (mud) |  | 8 May 2008 JP |
| C006-09 | 1 | 3.21 | Czech Republic, distr. Mělník, Přívory, Košátecký stream | 50°17'32"N, 14°34'57"E | 175 | stream (mud) |  | 8 May 2008 JP |
| C007-09 | 4 | **3.18±0.03** | Czech Republic, distr. Mělník, Kozly, ditch S of village | 50°14'57.6"N, 14°33'43.6"E | 165 | ditch |  | 8 May 2008 JP |
| C008-09 | 2 | 3.18 | Czech Republic, distr. Nymburk, Dvorce, in Mlynařice stream under road bridge E of village | 50°12'28.9"N, 14°48'8.0"E | 185 | stream (mud) |  | 9 May 2008 JP |
| C010-09 | 1 | 3.19 | Czech Republic, distr. Rakovník, Nové Strašecí, ditch on E edge of locality Podhůrka | 50°8'35"N, 13°53'24"E | 405 | ditch |  | 22 May 2009 JP |
| C013-09 | 1 | 3.18 | Czech Republic, Praha, city distr. Dolní Chabry, trough close to fishpond in street Pod hrází | 50°8'56.5"N, 14°26'25.0"E | 205 | stream (mud) |  | 24 May 2009 JP |
| C015-08 | 1 | 3.22 | Czech Republic, distr. Nymburk, Semice, ditch on E edge of village | 50°9'44.9"N, 14°52'57.3"E | 175 | ditch |  | 29 May 2009 JP |
| C019-09 | 1 | **3.23±0.02** | Czech Republic, distr. Česká Lípa, Hradčany, Hradčanské rybníky area, marsh below overflow of Strážovský fishpond | 50°36'39"N, 14°44'29"E | 275 | puddle | *C. hamulata* | 8 June 2009 JP |
| C022-09 | 1 | 3.20 | Czech Republic, distr. Česká Lípa, Břehyně, Břehyně-Pecopala area, ruts on path E of Břehyňský fishpond | 50°34'43"N, 14°43'42"E | 275 | puddle | *C. platycarpa* | 8 June 2009 JP |
| C031-09 | 1 | 3.22 | Czech Republic, distr. Litoměřice, Julčín, locality Na Černčí, pit in forest | 50°32'49.7"N 14°22'27.3"E | 305 | puddle |  | 19 June 2009 JP |
| C032-09 | 1 | 3.25 | Czech Republic, distr. Litoměřice, Tetčiněves, Úštěcký stream in village | 50°33'40."N, 14°20'49"E | 200 | stream (sand) |  | 19 June 2009 JP |
| C044-09 | 2 | 3.18 | Czech Republic, distr. Karviná, Dolní Marklovice, small fishpond close to state border | 49°53'7.3"N, 18°34'4.6"E | 227 | fishpond | *C. palustris* | 9 Aug. 2009 JP |
| C045-09 | 1 | **3.19±0.02** | Czech Republic, distr. Karviná, Dolní Marklovice, stream flowing out of small fishpond close to state border | 49°53'5.8"N, 18°34'3.4"E | 227 | stream (mud) |  | 9 Aug. 2009 JP |
| C047-09 | 1 | 3.21 | Czech Republic, distr. Karviná, Dolní Marklovice, small fishpond near motocross track N of village | 49°54'5.3"N, 18°34'10.5"E | 258 | fishpond |  | 9 Aug. 2009 JP |
| C051-09 | 1 | 3.15 | Czech Republic, distr. Karviná, Prstná, pool below outlet of fishpond on SW part of village | 49°54'50.4"N, 18°33'35.6"E | 236 | pool |  | 9 Aug. 2009 JP |
| C054-09 | 1 | **3.11±0.03** | Czech Republic, distr. Nový Jičín, Hukovice, marsh beside main forest road in forest Roveň | 49°37'19.3"N, 18°2'36.4"E | 275 | exposed bottom |  | 11 Aug. 2009 JP |
| C075-09 | 2 | 3.19 | Czech Republic, distr. Prostějov, Pohora, Podhora stream close to outlet of fishpond in village | 49°33'36.6"N, 16°45'11.0"E | 570 | stream (mud) |  | 25 Aug. 2009 JP |
| C078-09 | 1 | 3.20 | Czech Republic, distr. Blansko, Benešov, puddle on intersection of forest paths WNW of hill Skalky | 49°30'8.5"N, 16°47'2.3"E | 710 | puddle | *C. palustris* | 25 Aug. 2009 JP |
| C083-09 | 1 | 8.83 | Czech Republic, distr. Blansko, Žďárná, ruts on forest path SE of village | 49°26'58.3"N, 16°47'10.2"E | 575 | puddle | *C. palustris, C. stagnalis* | 26 Aug. 2009 JP |
| C091-09 | 1 | 3.22 | Czech Republic, distr. Brno-venkov, Ochoz u Brna, pool on Říčka stream SW of Svobodův Mlýn solitude | 49°14'3.8"N, 16°43'30.2"E | 290 | pool |  | 30 Aug. 2009 JP |
| C092-09 | 1 | 3.14 | Czech Republic, distr. České Budějovice, Staré Hutě, slough below dam of Mlýnský fishpond | 48°42'35.0"N, 14°42'43.2"E | 765 | puddle |  | 18 Sept. 2009 JP |
| C094-09b | 1 | **3.20±0.03** | Czech Republic, distr. České Budějovice, Staré Hutě, blind arm of Lužný stream WSW of settlement Mlýnský Vrch | 48°42'27.7"N, 14°41'56.2"E | 750 | pool | *C. hamulata* | 18 Sept. 2009 JP |
| C096-09 | 1 | **3.20±0.02** | Czech Republic, distr. České Budějovice, Staré Hutě, small ditch on plot of house in village | 48°43'11.9"N, 14°42'56.6"E | 797 | ditch |  | 20 Sept. 2009 JP |
| C101-09 | 1 | 3.20 | Czech Republic, distr. Pardubice, Dolní Jelení, ditch along forest path S of Oborecký fishpond | 50°2'5.9"N, 16°7'15.5"E | 285 | puddle |  | 8 Oct. 2009 JP |
| C102-09 | 3 | **3.20±0.01** | Czech Republic, distr. Pardubice, Rousínov, side arm of Čermná stream close to Horní Pecák fishpond | 50°2'57.1"N, 16°7'54.4"E | 275 | ditch | *C. stagnalis* | 8 Oct. 2009 JP |
| C104-09 | 1 | 3.19 | Czech Republic, distr. Pardubice, Korunka, Čermná stream in village | 50°3'48.7"N, 16°8'27.2"E | 265 | stream (mud) |  | 8 Oct. 2009 JP |
| C108-09b | 1 | 3.22 | Czech Republic, distr. Svitavy, Opatov, puddle on side of forest path S of railway station Semanín | 49°50'17.2"N, 16°28'17.2"E | 430 | puddle | *C. hamulata, C. palustris, C. stagnalis* | 19 Oct. 2009 JP |
| C111-09 | 1 | 3.24 | Czech Republic, distr. Svitavy, Opatov, puddle on forest path N of pond Rebelant | 49°50'1.3"N, 16°28'30.3"E | 436 | puddle |  | 19 Oct. 2009 JP |
| C112-09 | 3 | 3.22 | Czech Republic, distr. Svitavy, Opatov, small pond Rebelant | 49°49'49.7"N, 16°28'28.0"E | 440 | reservoir |  | 19 Oct. 2009 JP |
| C115-09 | 1 | 3.25 | Czech Republic, distr. Svitavy, Opatov, ditch along forest road SW of pond Rebelant | 49°49'32.5"N, 16°28'8.0"E | 450 | puddle |  | 19 Oct. 2009 JP |
| C116-09 | 4 | **3.17±0.00** | Czech Republic, distr. Svitavy, Opatovec, Terčový fishpond | 49°48'58.4"N, 16°28'52.9"E | 443 | fishpond |  | 19 Oct. 2009 JP |
| C003-10 | 2 | 3.14 | Czech Republic, distr. Olomouc, Horka nad Moravou, Častava stream on edge of Častava area | 49°38'31.3"N, 17°12'52.4"E | 215 | stream (mud) |  | 12 May 2010 JP |
| C004-10 | 1 | **3.19±0.03** | Czech Republic, distr. Olomouc, Horka nad Moravou, Častava stream near Podbradský fishpond | 49°38'39.5"N, 17°12'12.8"E | 215 | stream (mud) |  | 12 May 2010 JP |
| C005-10 | 1 | 3.16 | Czech Republic, distr. Olomouc, Hynkov, ditch SSE of village | 49°39'44.9"N, 17°10'29.0"E | 220 | ditch |  | 12 May 2010 JP |
| C006-10 | 1 | 3.16 | Czech Republic, distr. Olomouc, Štěpánov, ditch WNW of railway station | 49°40'33.2"N, 17°11'55.7"E | 220 | ditch |  | 12 May 2010 JP |
| C016-10 | 3 | **3.17±0.03** | Czech Republic, distr. Semily, Kadeřavec, small artificial pool WNW of Valdštejn castle | 50°33'53.8"N, 15°9'23.0"E | 375 | pool |  | 12 June 2010 JP |
| C019-10 | 1 | **3.17±0.00** | Czech Republic, distr. Břeclav, Hlohovec, ditch between small fishponds W of Hlohovecký fishpond | 48°46'46.6"N, 16°45'18.0"E | 170 | ditch |  | 7 June 2010 PT |
| C022-10 | 3 | **3.18±0.03** | Czech Republic, distr. Jičín, Ostroměř, millrace of Javorka stream | 50°22'30.0"N, 15°32'52.3"E | 265 | stream (sand) | *C. hamulata* | 26 June 2010 JP |
| C023-10 | 1 | 3.20 | Czech Republic, distr. Jičín, Šárovcova Lhota, ditch near settlement Mezihoří | 50°23'56.6"N, 15°32'34.1"E | 275 | ditch |  | 26 June 2010 JP |
| C032-10 | 1 | 3.18 | Czech Republic, distr. Zlín, Fryšták, stream in town distr. Dolní Ves | 49°16'31.3"N, 17°40'29.9"E | 246 | stream (mud) |  | 30 June 2010 JP |
| C034-10 | 2 | **3.19±0.02** | Czech Republic, distr. Zlín, Lukov, Bezedník fishpond | 49°17'59.1"N, 17°43'26.4"E | 320 | fishpond | *C. palustris* | 1 July 2010 JP |
| C042-10 | 1 | 3.26 | Czech Republic, distr. Zlín, Vlčková, puddle on forest path NE of hill Ondřejovsko | 49°19'52.6"N, 17°44'6.2"E | 570 | puddle | *C. stagnalis* | 1 July 2010 JP |
| C046-10 | 1 | 3.14 | Czech Republic, distr. Domažlice, Mezholezy, small fishpond on Mezholezský stream W of village | 49°37'21.2"N, 12°53'50.8"E | 439 | fishpond |  | 5 July 2010 JP & ZK |
| C048-10 | 2 | 3.13 | Czech Republic, distr. Domažlice, Mezholezy, small forest pond SE of rock formation Skalní mísy | 49°37'28.7"N, 12°52'41.6"E | 450 | reservoir |  | 5 July 2010 JP & ZK |
| C050-10 | 1 | 3.17 | Czech Republic, distr. Tachov, Racov, locality Racovské rybníčky, overgrown northern pond | 49°37'47.1"N, 12°51'25.0"E | 485 | pool |  | 5 July 2010 JP & ZK |
| C064-10 | 2 | 3.18 | Czech Republic, distr. Domažlice, Pila, locality Louka u Šnajberského rybníka, artificial pool N of Černý fishpond | 49°24'42.7"N, 12°51'55.8"E | 469 | pool |  | 9 July 2010 JP & M. Štech |
| C112-10 | 1 | 3.12 | Czech Republic, distr. Nymburk, Kostomlaty nad Labem, Vlkava stream od SW edge of village | 50°11'5.0"N, 14°56'46.6"E | 180 | stream (mud) |  | 2 Aug. 2010 JP |
| C133-10 | 1 | **3.20±0.02** | Czech Republic, distr. Rychnov nad Kněžnou, Čestice, meadow ditch W of railway station | 50°7'50.0"N, 16°7'59.4"E | 260 | ditch |  | 7 Aug. 2010 ZK |
| C135-10 | 1 | 3.23 | Czech Republic, distr. Chrudim, Rohozná, rivulet in alder carr close to NW edge of Velký Rohozenský fishpond | 49°48'37.8"N, 15°48'14.8"E | 578 | stream (mud) |  | 27 Aug. 2010 JP & ZK |
| C137-10 | 2 | 3.22 | Czech Republic, distr. Chrudim, Rohozná, Hubský fishpond | 49°48'27.2"N, 15°49'22.0"E | 575 | exposed bottom | *C. hamulata, C. palustris* | 27 Aug. 2010 JP & ZK |
| C143-10 | 1 | 3.22 | Czech Republic, distr. Havlíčkův Brod, Rovný, small forest fishpond NE of village | 49°44'29.0"N, 15°48'16.2"E | 570 | fishpond |  | 28 Aug. 2010 JP & ZK |
| C155-10 | 1 | 3.14 | Czech Republic, distr. Klatovy, Prášily, on bank at confluence of Křemelná and Prášilský streams | 49°8'5.2"N, 13°23'13.5"E | 810 | exposed bottom |  | 25 Sept. 2010 JP |
| C156-10 | 2 | 3.18 | Czech Republic, distr. Písek, Zátaví, Řežabinecký stream near estuary to Otava River | 49°16'16.4"N, 14°5'35.8"E | 367 | stream (mud) |  | 29 Sept. 2010 JP |
| C159-10 | 1 | 3.21 | Czech Republic, distr. Tábor, Hamr, Smíchov II fishpond | 49°9'36.7"N, 14°46'41.7"E | 420 | exposed bottom | *C. palustris* | 20 Sept. 2010 S. Píšová |
| C001-11 | 2 | 3.18 | Czech Republic, Praha, city distr. Klánovice, stream on W edge of village | 50°05'53.5"N, 14°38'54.6"E | 263 | stream (mud) |  | 11 May 2011 JP |
| C002-11 | 1 | **3.13±0.02** | Czech Republic, Praha, city distr. Horní Počernice, pool between SE edge of village and nameless fishpond | 50°05'56.0"N, 14°36'52.2"E | 245 | pool |  | 11 May 2011 JP |
| C004-11 | 1 | 3.19 | Czech Republic, distr. Ústí nad Orlicí, Horní Morava, on forest path NNE of quarry above village | 50°9'49.7"N 16°49'27.5"E | 795 | puddle |  | 14 May 2011 JP |
| C013-11 | 1 | 3.17 | Czech Republic, distr. Frýdek-Místek, Horní Lomná, puddles of forest path S of hill Úplaz | 49°31'0"N, 18°40'26"E | 905 | puddle |  | 12 June 2011 J. Prach |
| C041-11 | 1 | 3.23 | Poland, prov. Małopolskie, Jabłonka, marsh close to Czarna Orawa River WSW of village | 49°28'07.8"N, 19°40'22.8"E | 604 | puddle |  | 25 June 2011 JP & ZK |
| C047-11 | 1 | 3.21 | Slovakia, distr. Námestovo, Oravské Veselé, reservoir in the village | 49°27'45.0"N, 19°23'0.5"E | 711 | reservoir |  | 25 June 2011 JP & ZK |
| C062-11 | 1 | 3.13 | Czech Republic, distr. Praha-východ, Cyrilov, Šestajovický stream close to estuary to garden pond on SE edge of village | 50°6'3.7"N, 14°41'32.4"E | 250 | stream (mud) |  | 13 Aug. 2011 JP |
| C002-12 | 2 | 3.19 | Czech Republic, Praha, city distr. Horní Počernice, Xaverov, ditch NNW of hill Homole | 50°5'36.8"N, 14°37'43.9"E | 255 | ditch |  | 11 May 2012 JP |
| C004-12 | 1 | 3.24 | Czech Republic, Praha, city distr. Běchovice, ditch between village parts Běchovice I and Běchovice II | 50°4'53.1"N, 14°36'32.7"E | 235 | ditch |  | 11 May 2012 JP |
| C005-12 | 1 | 3.18 | Czech Republic, Mělník, Pšovka stream in town | 50°21'22.6"N, 14°29'0.8"E | 165 | stream (sand) |  | 13 May 2012 JP |
| C016-12 | 1 | 3.18 | Czech Republic, distr. Rakovník, Třtice, littoral zone of small pond close to solitude Třtická Lísa | 50°9'49.6"N, 13°50'20.4"E | 450 | exposed bottom | *C. hamulata* | 12 June 2012 JP & A. Potůčková |
| C017-12 | 4 | 3.18 | Czech Republic, distr. Rakovník, Třtice, locality V Bahnách, artificial pool | 50°10'27.2"N, 13°51'38.3"E | 418 | pool |  | 12 June 2012 JP & A. Potůčková |
| C025-12 | 1 | 3.25 | Slovakia, distr. Zlaté Moravce, Jelenec, on N bank of Jelenec fishpond | 48°24'4.1"N, 18°12'13.6"E | 234 | exposed bottom | *C. palustris* | 4 July 2012 JP |
| C033-12 | 5 | **3.25±0.01** | Denmark, Jutland, reg. Syddanmark, Seem, estuary of field ditch to Ribeå River ESE of town | 55°18'54.8"N, 08°50'04.0"E | 1 | stream (mud) |  | 31 July 2012 JP & ZK |
| C063-12 | 1 | **3.16±0.04** | Czech Republic, distr. Ústí nad Orlicí, Brandýs nad Orlicí, Tichá Orlice River below bridge of Žerotínova street | 50°0'4.9"N, 16°16'45.1"E | 295 | river (sand) | *C. hamulata, C. hamulata × C. cophocarpa?* | 15 Aug. 2012 JP |
| C074-12 | 1 | **3.12±0.02** | Poland, prov. Podlaskie/Mazowieckie, Tyszki-Gostery, Ruż stream near bridge of Droga Wojewódzka road NE of village | 52°59'33.0"N, 21°54'58.4"E | 111 | stream (sand) |  | 31 Aug. 2012 PT & B. Kubátová |
| C001-13 | 1 | 3.26 | Czech Republic, distr. Olomouc, Jívová, pool in valley of Bystřice stream near settlement Panský Mlýn | 49°42'21.3"N, 17°25'56.4"E | 404 | pool |  | 29 Apr. 2013 JP & KK |
| C011-13 | 1 | 3.19 | Czech Republic, distr. Hradec Králové, Pamětník, Mlýnská Cidlina stream in village | 50°07'25.9"N, 15°26'51.2"E | 210 | stream (mud) |  | 28 May 2013 JP |
| C013-13 | 1 | 3.20 | Czech Republic, distr. Břeclav, Kostice, pools in forest Tvrdonické polesí SE of village | 48°44'14.0"N, 17°00'21.9"E | 150 | pool | *C. hamulata* | 4 June 2013 JP & M. Hrdinová |
| C015-13 | 2 | 3.20 | Czech Republic, distr. Břeclav, Lanžhot, old separated arm of Morava River near border crossing of D2 highway | 48°41'29.9"N, 16°59'34.6"E | 150 | pool |  | 4 June 2013 JP & M. Hrdinová |
| C016-13 | 1 | **3.24±0.03** | Slovakia, distr. Galanta, Pusté Úľany, Nový stream close to road bridge SW of village | 48°13'35.2"N, 17°33'52.1"E | 115 | stream (mud) |  | 5 June 2013 JP & M. Hrdinová |
| C017-13 | 3 | 3.24 | Slovakia, distr. Senec, Hamuliakovo, canal in village near reservoir Hrušov on Dunaj River | 48°2'1.9"N, 17°14'55.3"E | 124 | stream (sand) |  | 6 June 2013 JP & M. Hrdinová |
| C019-13 | 1 | 3.24 | Czech Republic, distr. Příbram, Mokrovraty, puddle on side of forest path NE of hill Dolní Jezberná | 49°48'45.5"N, 14°14'33.6"E | 345 | puddle |  | 11 June 2013 JP & KK |
| C036-13 | 2 | 3.13 | Czech Republic, distr. Příbram, Mokrovraty, Dolní (Tušimský) fishpond | 49°48'23.7"N, 14°13'50.6"E | 380 | fishpond | *C. hamulata* | 11 June 2013 JP & KK |
| C027-13 | 1 | 3.20 | Czech Republic, distr. Svitavy, Třebařov, Rohlíček fishpond | 49°50'40.5"N, 16°40'35.1"E | 340 | fishpond |  | 20 June 2013 JP & ZK |
| C029-13 | 2 | 3.25 | Czech Republic, distr. Olomouc, Nové Zámky, old separated arm of Morava River in locality Pod Templem | 49°43'14.8"N, 17°01'01.4"E | 240 | pool | *C. hamulata* | 21 June 2013 JP & ZK |
| C030-13 | 1 | 3.23 | Czech Republic, distr. Přerov, Týn nad Bečvou, pool complex in N part of locality Týn nad Bečvou | 49°31'37.2"N, 17°38'33.3"E | 234 | pool |  | 21 June 2013 JP & ZK |
| C031-13 | 1 | 3.25 | Czech Republic, distr. Přerov, Tovačov, Splavská canal on SW edge of town | 49°25'39.8"N, 17°17'03.3"E | 199 | stream (mud) |  | 21 June 2013 JP & ZK |
| C038-13 | 1 | 3.18 | Czech Republic, distr. Jičín, Kbelnice, ditch in village | 50°27'18"N, 15°20'48"E | 280 | ditch |  | 4 July 2013 V. Sedláček & M. Chytrý |
| C039-13 | 2 | 3.15 | Czech Republic, distr. Trutnov, Borovnička, stream in alder carr SW of village | 50°29'41"N, 15°39'5"E | 470 | stream (sand) |  | 4 July 2013 P. Koutecký |
| C046-13 | 1 | 3.16 | Czech Republic, distr. Trutnov, Choustníkovo Hradiště, small fishpond on Hradišťský stream below caste in village | 50°25'47"N, 15°52'46"E | 300 | fishpond |  | 4 July 2013 JP |
| C063-13 | 1 | **3.17±0.05** | Czech Republic, distr. Cheb, Nový Drahov, settlement Hájek, marsh on meadow in S part of Soos area | 50°8'39.8"N, 12°24'49.4"E | 432 | puddle |  | 19 July 2013 KK & JarR |
| C066-13 | 2 | **3.22±0.04** | Czech Republic, distr. Cheb, Dvorek, Stodolský stream by road bridge NE of village | 50°8'19.5"N, 12°24'42.5"E | 431 | stream (sand) | *C. hamulata, C. stagnalis* | 19 July 2013 KK & JarR |
| C071-13 | 1 | **3.26** | Sweden, prov. Västernorrland, Bollstabruk, ditch along road no. 90 NNW of village | 63°3'18.0"N, 17°36'49.2"E | 98 | ditch |  | 3 Aug. 2013 JanR |
| C081-13 | 6 | **3.22±0.02** | Czech Republic, distr. Jablonec nad Nisou, Bedřichov, Blatný rybník reservoir | 50°48'36.9"N, 15°9'53.8"E | 770 | reservoir | *C. hamulata* | 22 Aug. 2013 JP & KK |
| C088-13 | 1 | 3.27 | Czech Republic, distr. Hradec Králové, Blešno, pool in alluvium of Orlice River S of village | 50°12'33.1"N, 15°55'45.5"E | 225 | exposed bottom |  | 1 Sept. 2013 JP & KK |
| C095-13 | 2 | **3.19±0.03** | Czech Republic, distr. Ústí nad Orlicí, Choceň, town distr. Peliny, confluence of outlet of separated river arm to Tichá Orlice River | 49°59'55.7"N, 16°14'17.4"E | 295 | river (mud) | *C. hamulata* | 1 Sept. 2013 JP & KK |
| C119-13 | 1 | **3.24±0.04** | Czech Republic, distr. Ústí nad Labem, Petrovice, artificial pool ENE of border crossing | 50°48'55"N, 13°59'3"E | 430 | pool |  | 27 Aug. 2013 JanR & JarR |
| C126-13 | 1 | 3.19 | Czech Republic, distr. Nymburk, Libice nad Cidlinou, Libický luh forest, pool Malý Přerov near confluence of Labe and Cidlina Rivers | 50°7'12.2"N, 15°9'43.7"E | 185 | pool |  | 1 Sept. 2013 JarR |
| C128-13 | 5 | 3.15 | Czech Republic, distr. Jihlava, Řásná, Velký Pařezitý fishpond | 49°13'41.4"N, 15°22'44.5"E | 675 | fishpond | *C. hamulata* | 8 Sept. 2013 JP |
| C131-13 | 1 | 3.21 | Czech Republic, distr. Jihlava, Řídelov, ditch along forest path near SW bank of Pilný fishpond | 49°14'08.7"N, 15°23'52.6"E | 620 | ditch | *C. stagnalis* | 8 Sept. 2013 JP |
| C138-13 | 1 | 3.20 | Czech Republic, distr. Frýdek-Místek, Horní Lomná, puddle on forest path NNE of Kalužný hill NNW of village | 49°34'24.2"N, 18°37'37.4"E | 975 | puddle |  | 16 Sept. 2013 JP & KK |
| C139-13 | 3 | 3.24 | Czech Republic, distr. Frýdek-Místek, Horní Lomná, puddle on forest path near observation tower Kozubová | 49°34'13.1"N, 18°40'24.7"E | 975 | puddle | *C. palustris* | 16 Sept. 2013 JP & KK |
| C141-13 | 1 | 3.23 | Slovakia (on borderline with Poland), distr. Námestovo, Vychylovka, puddle on ridge of forest road NE of Jaworzyna mountain | 49°23'58.4"N, 19°02'34.2"E | 1091 | puddle |  | 17 Sept. 2013 JP & KK |
| C142-13 | 1 | 3.24 | Slovakia (on borderline with Poland), distr. Námestovo, Novoť, puddle on ridge of forest road N of Úšust (Ozsust) mountain | 49°25'26.8"N, 19°11'30.2"E | 958 | puddle |  | 18 Sept. 2013 JP & KK |
| C143-13 | 3 | 3.23 | Slovakia (on borderline with Poland), distr. Námestovo, Mútňanská Píla, puddle on ridge of forest road WNW of village | 49°28'49.9"N, 19°13'31.4"E | 1074 | puddle |  | 17 Sept. 2013 JP & KK |
| C145-13 | 1 | 3.26 | Czech Republic, Olomouc, field ditch SW of city distr. Holice | 49°33'46"N, 17°17'19"E | 205 | ditch |  | 23 Sept. 2013 JarR |
| C147-13 | 1 | 3.18 | Czech Republic, distr. Nymburk, Veleliby, Liduška stream below bridge W of village | 50°12'41"N, 15°1'1"E | 195 | stream (mud) |  | 11 Oct. 2013. KK & JarR |
| C148-13 | 1 | 3.15 | Czech Republic, distr. Nymburk, Veleliby, Liduška stream below Zdonín farm | 50°12'13"N, 15°1'32"E | 190 | stream (mud) |  | 11 Oct. 2013. KK & JarR |
| C149-13 | 1 | 3.16 | Czech Republic, Nymburk, Liduška stream below railway bridge on NW edge of town | 50°11'39"N, 15°1'52"E | 185 | stream (mud) |  | 11 Oct. 2013. KK & JarR |
| ***C. hamulata* Kütz. ex W.D.J. Koch** | | | | | | | | |
|  |  |  |  |  |  |  |  |  |
| C009-07b | 1 | 9.01 | Czech Republic, distr. Klatovy, Sušice, Roušarka stream on S edge of town | 49°13'12.9"N, 13°30'33.5"E | 480 | stream (sand) | *C. cophocarpa* | 29 June 2007 JP |
| C017-07 | 1 | 8.83 | Czech Republic, distr. Mělník, Hořín, pool on left bank of Vltava River | 50°19'51.3"N, 14°28'35.1"E | 160 | exposed bottom |  | 8 Aug. 2007 JP |
| C021-07 | 1 | 9.00 | Czech Republic, distr. Rakovník, Řevničov, locality Prameny Klíčavy, puddle on forest path | 50°8'38.2"N, 13°49'37.2"E | 420 | puddle | *C. stagnalis* | 16 Aug. 2007 JP |
| C023-07 | 1 | 8.88 | Czech Republic, distr. Kladno, Lány, forest puddle near solitude Nový Dvůr | 50°8'57"N, 13°55'37"E | 425 | puddle | *C. stagnalis* | 16 Aug. 2007 JP |
| C036-07 | 1 | 9.05 | Czech Republic, distr. České Budějovice, Třebeč, Brouskův mlýn area, Stropnice stream | 48°52'52"N, 14°40'58"E | 450 | stream (sand) |  | 29 June 2007 ZK |
| C038-07 | 1 | 8.79 | Czech Republic, distr. Tábor, Bechyňská Smoleč, puddle on forest path | 49°18'35.6"N 14°33'33.7"E | 490 | puddle |  | 15 July 2007 H. Chudáčková |
| C001-08b | 1 | 8.99 | Czech Republic, Praha, city distr. Modřany, pools "Modřanské laguny" on right bank of Vltava River | 49°59'48.3"N, 14°24'11.1"E | 190 | pool | *C. cophocarpa* | 1 May 2008 JP |
| C006-08b | 1 | 8.90 | Czech Republic, distr. Chrudim, Rabštejnská Lhota, small pond on Podhůra stream | 49°54'28.5"N, 15°46'31.6"E | 315 | fishpond | *C. cophocarpa* | 23 May 2008 JP |
| C011-08 | 2 | 8.87 | Czech Republic, distr. Chrudim, Trhová Kamenice, settlement Petrkov, puddle on forest path | 49°47'55.8"N, 15°50'29.7"E | 575 | puddle |  | 24 May 2008 JP |
| C012-08 | 1 | 8.92 | Czech Republic, distr. Chrudim, Hamry, Chrudimka River in village | 49°44'22.6"N, 15°55'32.6"E | 585 | river (sand) |  | 25 May 2008 JP |
| C013-08 | 1 | **8.87±0.04** | Czech Republic, distr. Chrudim, Hamry, small fishpond SSE of village | 49°43'52.5"N, 15°55'49.9"E | 615 | fishpond |  | 25 May 2008 JP |
| C014-08 | 2 | 8.87 | Czech Republic, distr. Chrudim, Hamry, Chrudimka River SSE of village | 49°43'38.6"N, 15°56'8.5"E | 605 | river (sand) |  | 25 May 2008 JP |
| C027-08 | 1 | **8.85±0.06** | Czech Republic, distr. Děčín, Mezná, Kamenice stream in Divoká soutěska canyon | 50°51'50.0"N, 14°19'10.5"E | 170 | stream (sand) |  | 3 July 2008 JP |
| C029-08 | 1 | **8.96±0.05** | Czech Republic, distr. Jindřichův Hradec, Majdalena, Lužnice River in Stará řeka area | 48°59'17"N, 14°50'54"E | 435 | river (sand) |  | 10 July 2008 JP |
| C032-08 | 2 | 8.97 | Czech Republic, distr. Jindřichův Hradec, Hamr, Koštěnický stream | 48°57'8.3"N, 14°54'54.4"E | 450 | stream (sand) |  | 10 July 2008 JP |
| C044-08 | 2 | **8.88±0.03** | Czech Republic, distr. Praha-východ, Jevany, middle forest fishpond in Aldašín deer-park | 49°58'6.6"N, 14°50'21.2"E | 385 | exposed bottom | *C. palustris*, *C. stagnalis* | 19 July 2008 JP |
| C048-08 | 2 | 8.84 | Czech Republic, distr. Cheb, Krapice, Podsedek fishpond | 50°6'52"N, 12°19'26"E | 440 | exposed bottom |  | 1 Aug. 2008 JP |
| C049-08 | 1 | 8.85 | Czech Republic, distr. Cheb, Františkovy Lázně, settlement Lužná, puddle on forest path near Vražedný fishpond | 50°6'19"N, 12°17'16"E | 450 | puddle | *C. stagnalis* | 1 Aug. 2008 JP |
| C052-08 | 1 | 8.86 | Czech Republic, distr. Cheb, Ostroh, Slatinný stream | 50°7'58"N, 12°16'54"E | 490 | stream (sand) |  | 1 Aug. 2008 JP |
| C056-08 | 1 | 8.87 | Czech Republic, distr. Cheb, Podílná, puddle on forest path NE of Čtvrtý fishpond | 50°8'56"N, 12°13'27"E | 575 | puddle | *C. palustris* | 2 Aug. 2008 JP |
| C059-08 | 1 | 8.97 | Czech Republic, distr. Cheb, Skalná, Sázek stream near settlement Kateřina | 50°9'34"N, 12°24'24"E | 435 | stream (sand) |  | 3 Aug. 2008 JP |
| C060-08 | 1 | 8.81 | Czech Republic, distr. Cheb, Nová Ves, pool in kaolin quarry SSW of village | 50°9'42"N, 12°24'42"E | 440 | pool | *C. cophocarpa* | 3 Aug. 2008 JP |
| C064-08 | 2 | 8.77 | Czech Republic, distr. Sokolov, Kynšperk nad Ohří, Ohře River in camp | 50°7'32.5"N, 12°31'46.3"E | 415 | river (sand) |  | 3 Aug. 2008 JP |
| C069-08 | 2 | 8.84 | Czech Republic, distr. Liberec, Černousy, Smědá stream S of railway station | 51°0'6"N, 15°2'16"E | 220 | stream (mud) | *C. platycarpa* | 19 Aug. 2008 JP |
| C077-08 | 2 | 8.72 | Czech Republic, distr. Prachatice, Volary, settlement Soumarský Most, Vltava River in camp | 48°54'28.0"N, 13°49'37.7"E | 750 | river (sand) |  | 6 Sept. 2008 JP |
| C079-08 | 3 | 8.82 | Czech Republic, distr. Prachatice, Stožec, millrace of Studená Vltava stream | 48°51'31.8"N, 13°49'11.6"E | 780 | stream (sand) |  | 6 Sept. 2008 JP |
| C085-08 | 1 | 9.02 | Czech Republic, distr. Havlíčkův Brod, Hluboká, puddle on path NW of village | 49°39'51"N, 15°50'50"E | 560 | puddle |  | 22 Sept. 2008 JP |
| C088-08 | 1 | 8.97 | Czech Republic, distr. Žďár nad Sázavou, Karlov, Stružný stream (Sázava) near estuary to Velké Dářko fishpond | 49°38'48"N, 15°53'45"E | 615 | stream (sand) |  | 22 Sept. 2008 JP |
| C090-08 | 1 | 8.75 | Czech Republic, distr. Žďár nad Sázavou, Tři Studně, on path near NE bank of Medlovský fishpond | 49°36'52"N, 16°3'26"E | 715 | puddle |  | 23 Sept. 2008 JP |
| C091-08 | 3 | 8.83 | Czech Republic, distr. Žďár nad Sázavou, Milovy, Svratka stream, southernmost meander | 49°40'11"N, 16°5'26"E | 595 | stream (sand) |  | 23 Sept. 2008 JP |
| C105-08 | 1 | 8.86 | Czech Republic, distr. Mladá Boleslav, Bělá pod Bezdězem, village distr. Vrchbělá, puddle on forest path | 50°31'17"N, 14°46'38"E | 315 | puddle | *C. stagnalis* | 28 Sept. 2008 J. Suda |
| C004-09 | 2 | **8.83±0.08** | Czech Republic, distr. Klatovy, Rejštejn, Radešov settlement, Opolenecký stream near estuary to Otava River | 49°9'11.1"N, 13°30'48.4"E | 550 | stream (sand) |  | 30 Apr. 2009 JP |
| C016-09 | 1 | 9.01 | Czech Republic, distr. Česká Lípa, Mimoň, Ploučnice River in town | 50°39'32.8"N, 14°43'38.2"E | 280 | river (sand) |  | 8 June 2009 JP |
| C018-09 | 2 | **9.02±0.09** | Czech Republic, distr. Česká Lípa, Boreček, Ploučnice River S of settlement | 50°37'51.6"N, 14°43'5.7"E | 270 | river (sand) |  | 8 June 2009 JP |
| C020-09 | 1 | 9.16 | Czech Republic, distr. Česká Lípa, Hradčany, Hradčanské rybníky area, marsh below overflow of Strážovský fishpond | 50°36'39"N, 14°44'29"E | 275 | puddle | *C. cophocarpa* | 8 June 2009 JP |
| C027-09 | 1 | **9.04±0.07** | Czech Republic, Česká Lípa, Ploučnice River in town | 50°40'58.4"N, 14°32'15.9"E | 245 | river (sand) |  | 9 June 2009 JP |
| C046-09 | 1 | **8.85±0.05** | Czech Republic, distr. Karviná, Dolní Marklovice, Petrůvka stream in village | 49°53'31.8"N, 18°33'59.5"E | 225 | stream (sand) |  | 9 Aug. 2009 JP |
| C058-09 | 3 | 8.84 | Czech Republic, distr. Děčín, Mikulášovice, Mikulášovický stream in NW part of town | 50°58'50.7"N, 14°20'36.7"E | 385 | stream (mud) | *C. platycarpa* | 18 Aug. 2009 JP |
| C059-09 | 1 | 8.87 | Czech Republic, distr. Děčín, Mikulášovice, former pond E of settlement Tomášov | 50°57'24.8"N, 14°20'33.3"E | 470 | pool |  | 18 Aug. 2009 JP |
| C060-09 | 1 | 8.89 | Czech Republic, distr. Děčín, Mikulášovice, outdoor swimming pool, southern reservoir | 50°57.5'N, 14°21.8'E | 430 | reservoir |  | 18 Aug. 2009 JP |
| C061-09 | 1 | 8.89 | Czech Republic, distr. Děčín, Mikulášovice, small fishpond S of railway | 50°57'24.5"N, 14°22'33.4"E | 455 | exposed bottom |  | 18 Aug. 2009 JP |
| C064-09 | 2 | **8.88±0.08** | Czech Republic, distr. Děčín, Velký Šenov, Vilémovský stream in town | 50°59'24.3"N, 14°22'36.4"E | 350 | stream (mud) | *C. platycarpa* | 18 Aug. 2009 JP |
| C069-09 | 2 | 8.87 | Czech Republic, distr. Děčín, Šluknov, fishpond on SE part of settlement Nové Hraběcí | 51°0'40.3"N, 14°26'39.8"E | 350 | fishpond |  | 19 Aug. 2009 JP |
| C071-09 | 5 | 8.97 | Czech Republic, distr. Děčín, Šluknov, Stříbrný stream in town | 51°0'19.9"N, 14°27'5.7"E | 340 | stream (sand) | *C. platycarpa* | 19 Aug. 2009 JP |
| C072-09 | 2 | 8.95 | Czech Republic, distr. Děčín, Šluknov, southern pond in chateau park | 51°0'20.1"N, 14°27'17.6"E | 340 | fishpond |  | 19 Aug. 2009 JP |
| C080-09 | 1 | 8.87 | Czech Republic, distr. Blansko, Žďárná, on forest path NW of settlement Skelná Huť | 49°28'42.4"N, 16°47'12.4"E | 670 | puddle |  | 25 Aug. 2009 JP |
| C090-09 | 1 | 8.94 | Czech Republic, distr. Blansko, Vilémovice, locality Vývěry Punkvy, reappearing of subterranean Punkva stream from Punkevní caves | 49°22'14.0"N, 16°43'31.7"E | 375 | pool |  | 28 Aug. 2009 JP |
| C093-09 | 1 | **8.88±0.08** | Czech Republic, distr. České Budějovice, Staré Hutě, Lužný stream below outlet from Mlýnský fishpond | 48°42'33.5"N, 14°42'45.2"E | 767 | exposed bottom |  | 18 Sept. 2009 JP |
| C094-09a | 2 | 8.81 | Czech Republic, distr. České Budějovice, Staré Hutě, blind arm of Lužný stream WSW of settlement Mlýnský Vrch | 48°42'27.7"N, 14°41'56.2"E | 750 | pool | *C. cophocarpa* | 18 Sept. 2009 JP |
| C099-09 | 3 | **8.87±0.03** | Czech Republic, distr. Pardubice, Dolní Jelení, Oborecký fishpond | 50°2'29.6"N, 16°7'14.4"E | 280 | exposed bottom | *C. palustris* | 8 Oct. 2009 JP |
| C108-09a | 2 | 9.01 | Czech Republic, distr. Svitavy, Opatov, puddle on side of forest path S of railway station Semanín | 49°50'17.2"N, 16°28'17.2"E | 430 | puddle | *C. cophocarpa, C. palustris, C. stagnalis* | 19 Oct. 2009 JP |
| C109-09 | 1 | **8.99±0.08** | Czech Republic, distr. Svitavy, Opatov, puddle on forest path NNW of pond Rebelant | 49°50'2.7"N, 16°28'19.4"E | 437 | puddle | *C. stagnalis* | 19 Oct. 2009 JP |
| C114-09 | 1 | 8.83 | Czech Republic, distr. Svitavy, Opatov, ruts on forest path NW of pond Rebelant | 49°49'53.5"N, 16°28'20.0"E | 440 | puddle | *C. stagnalis* | 19 Oct. 2009 JP |
| C001-10 | 1 | **8.83±0.08** | Czech Republic, distr. Příbram, Sychrov, ditch on edge of forest meadow NNE of village | 49°46'8.2"N, 14°7'20.4"E | 420 | ditch |  | 8 May 2010 JP |
| C007-10 | 1 | **8.96±0.06** | Czech Republic, Praha, city distr. Modřany, pool connected to Vltava River | 49°59'52.7"N, 14°24'13.0"E | 185 | pool |  | 27 May 2010 JP |
| C012-10 | 1 | **8.91±0.05** | Czech Republic, Most, Bílina River in city | 50°30'57.0"N, 13°38'51.2"E | 221 | river (mud) |  | 2 June 2010 JP |
| C014-10 | 3 | **9.06±0.10** | Czech Republic, distr. Semily, Turnov, small ponds below castle Hrubý Rohozec | 50°35'58.6"N, 15°9'35.6"E | 246 | reservoir | *C. palustris*, *C. platycarpa* | 12 June 2010 JP |
| C021-10 | 3 | **8.84±0.06** | Czech Republic, distr. Jičín, Ostroměř, millrace of Javorka stream | 50°22'30.0"N, 15°32'52.3"E | 265 | stream (sand) | *C. cophocarpa* | 26 June 2010 JP |
| C026-10 | 1 | 8.75 | Czech Republic, distr. Jičín, Lázně Bělohrad, Bělohradská bažantnice pheasantry, pool Hraběnčino jezírko | 50°25'30.4"N, 15°35'53.6"E | 295 | pool |  | 26 June 2010 JP |
| C052-10 | 1 | **9.02±0.02** | Czech Republic, distr. Domažlice, Mezholezy, forest pool near western bank of Mezholezský fishpond | 49°37'45.6"N, 12°53'31.3"E | 435 | exposed bottom |  | 5 July 2010 JP & ZK |
| C071-10 | 1 | 8.89 | Czech Republic, Domažlice, Zubřina stream in town | 49°26'18.8"N, 12°55'41.9"E | 415 | stream (sand) |  | 5 July 2010 P. Koutecký |
| C079-10 | 1 | 8.85 | Czech Republic, distr. Klatovy, Horská Kvilda, Hamerský stream WSW of village | 49°3'18.7"N, 13°32'54.1"E | 1024 | stream (sand) |  | 17 July 2010 JP |
| C082-10 | 1 | 8.93 | Czech Republic, distr. Sokolov, Liboc, Libocký stream in village | 50°7'18.5"N, 12°31'0.9"E | 415 | stream (sand) |  | 19 July 2010 JP & ZK |
| C083-10 | 2 | 9.04 | Czech Republic, distr. Sokolov, Chotíkov, Ohře River in village | 50°6'58.0"N, 12°30'18.2"E | 413 | river (sand) |  | 19 July 2010 JP & ZK |
| C084-10 | 1 | 8.96 | Czech Republic, distr. Cheb, Mostov, Ohře River NW of village | 50°7'1.2"N, 12°29'22.1"E | 416 | river (sand) |  | 19 July 2010 JP & ZK |
| C085-10 | 1 | 8.98 | Czech Republic, distr. Cheb, Mostov, Ohře River in village | 50°6'54.3"N, 12°29'22.0"E | 416 | river (sand) |  | 19 July 2010 JP & ZK |
| C086-10 | 1 | 9.02 | Czech Republic, distr. Cheb, Mostov, blind arm of Ohře River | 50°06'57.4"N, 12°29'16.2"E | 416 | pool |  | 19 July 2010 JP & ZK |
| C091-10 | 1 | 8.87 | Czech Republic, distr. Liberec, Hrádek nad Nisou, Lužická Nisa River in town | 50°50'55.4"N, 14°50'50.3"E | 245 | river (sand) |  | 28 July 2010 JP |
| C093-10 | 2 | 8.89 | Czech Republic, distr. Liberec, Jablonné v Podještědí, Panenský stream between Pivovarský and Markvartický fishponds | 50°46'33.1"N, 14°46'54.1"E | 310 | stream (mud) | *C. platycarpa* | 28 July 2010 JP |
| C094-10 | 1 | 8.81 | Czech Republic, distr. Liberec, Jablonné v Podještědí, town distr. Zámecká, Panenský stream | 50°45'44.6"N, 14°45'28.4"E | 305 | stream (sand) |  | 28 July 2010 JP |
| C102-10 | 1 | 8.86 | Czech Republic, distr. Česká Lípa, Kunratice u Cvikova, Svitávka stream in village | 50°46'8.7"N, 14°40'43.4"E | 320 | stream (sand) |  | 28 July 2010 JP |
| C104-10 | 3 | 8.97 | Czech Republic, distr. Česká Lípa, Radvanec, Dobranovský stream close to estuary to Radvanecký fishpond | 50°44'59.2"N, 14°35'43.7"E | 294 | stream (mud) | *C. platycarpa* | 28 July 2010 JP |
| C106-10 | 4 | 8.72 | Czech Republic, distr. Česká Lípa, Sloup v Čechách, Dobranovský stream in village | 50°44'34.6"N, 14°35'32.2"E | 290 | stream (mud) | *C. platycarpa* | 29 July 2010 JP |
| C118-10 | 2 | 8.78 | Czech Republic, distr. Ústí nad Orlicí, Hnátnice, Tichá Orlice River close to railway station | 50°0'7.6"N, 16°27'38.1"E | 337 | river (sand) |  | 11 Aug. 2010 JP |
| C120-10 | 1 | 8.83 | Czech Republic, Ústí nad Orlicí, Tichá Orlice River in town | 49°58'30.5"N, 16°23'11.8"E | 324 | river (sand) |  | 12 Aug. 2010 JP |
| C121-10 | 3 | 8.79 | Czech Republic, distr. Ústí nad Orlicí, Sudislav nad Orlicí, Tichá Orlice River ESE of village | 49°59'2.2"N, 16°20'6.6"E | 315 | river (sand) |  | 12 Aug. 2010 JP |
| C136-10 | 2 | 8.91 | Czech Republic, distr. Chrudim, Rohozná, Hubský fishpond | 49°48'27.2"N, 15°49'22.0"E | 575 | exposed bottom | *C. cophocarpa, C. palustris* | 27 Aug. 2010 JP & ZK |
| C139-10 | 1 | **8.97±0.09** | Czech Republic, distr. Chrudim, Trhová Kamenice, Chrudimka River in village | 49°47'9.2"N, 15°48'53.5"E | 525 | river (sand) |  | 28 Aug. 2010 JP & ZK |
| C142-10 | 1 | 8.95 | Czech Republic, distr. Havlíčkův Brod, Kocourov, Návesní fishpond | 49°45'9.5"N, 15°47'59.9"E | 552 | fishpond |  | 28 Aug. 2010 JP & ZK |
| C145-10 | 3 | **9.04±0.10** | Czech Republic, distr. Havlíčkův Brod, Stružinec, Januš fishpond | 49°44'11.8"N, 15°50'43.5"E | 548 | exposed bottom | *C. palustris* | 28 Aug. 2010 JP & ZK |
| C146-10 | 1 | 8.93 | Czech Republic, distr. Chrudim, Vortová, Návesník fishpond | 49°42'45.9"N, 15°55'45.3"E | 617 | fishpond |  | 28 Aug. 2010 JP & ZK |
| C147-10 | 5 | **8.89±0.03** | Czech Republic, distr. Žďár nad Sázavou, Račín, locality Mlýnský potok a Uhlířky, Losenický stream SW of village | 49°36'33.7"N, 15°50'52.6"E | 597 | stream (sand) |  | 29 Aug. 2010 JP & ZK |
| C151-10 | 1 | 9.02 | Czech Republic, distr. Děčín, Šluknov, Spréva (Spree) stream on state border in former village Fukov | 51°2'37"N, 14°30'15"E | 300 | stream (sand) | *C. platycarpa* | 9 Sept. 2010 P. Bauer |
| C153-10 | 1 | 8.75 | Czech Republic, distr. Prachatice, Borová Lada, Vltava River in village | 48°59'26.5"N, 13°39'41.8"E | 892 | stream (sand) |  | 25 Sept. 2010 JP |
| C014-11 | 2 | 8.72 | Czech Republic, distr. Klatovy, Dobršín, Otava River S of village | 49°15'18.5"N, 13°33'40.1"E | 453 | river (sand) |  | 17 June 2011 JP & P. Koutecký |
| C015-11 | 1 | 8.84 | Czech Republic, distr. Klatovy, Čepice, Otava River W of village | 49°15'59.7"N, 13°35'17.1"E | 450 | river (sand) |  | 17 June 2011 JP & P. Koutecký |
| C017-11 | 3 | 8.72 | Czech Republic, distr. Klatovy, Čepice, blind arm on right bank of Otava River ESE of village | 49°15'57.5"N, 13°36'3.7"E | 445 | pool | *C. ×vigens* | 17 June 2011 JP & P. Koutecký |
| C019-11 | 1 | 8.80 | Czech Republic, distr. Klatovy, Čepice, blind arm on leftbank of Otava River ESE of village | 49°15'58.2"N, 13°36'13.0"E | 445 | pool | *C. ×vigens* | 17 June 2011 JP & P. Koutecký |
| C021-11 | 3 | 8.70 | Czech Republic, distr. Klatovy, Velké Hydčice, blind arm of Otava River N of village | 49°18'19.5"N, 13°40'9.8"E | 426 | pool | *C. ×vigens* | 17 June 2011 JP & P. Koutecký |
| C026-11 | 1 | 8.83 | Czech Republic, distr. Klatovy, Horažďovice, on alluvium of Otava River below weir on SE edge of town | 49°18'52.9"N, 13°43'8.3"E | 415 | exposed bottom |  | 18 June 2011 JP & P. Koutecký |
| C029-11 | 1 | 8.80 | Czech Republic, distr. Strakonice, Střelské Hoštice, Otava River on S edge of village | 49°17'38.8"N, 13°45'21.2"E | 408 | river (sand) |  | 18 June 2011 JP & P. Koutecký |
| C030-11 | 1 | **8.77±0.07** | Czech Republic, distr. Strakonice, Horní Poříčí, Otava River below weir | 49°17'4.3"N, 13°46'47.7"E | 405 | river (sand) |  | 18 June 2011 JP & P. Koutecký |
| C033-11 | 1 | 8.82 | Czech Republic, distr. Strakonice, Katovice, on alluvium of Otava River below weir in village | 49°16'20.7"N, 13°49'30.7"E | 400 | exposed bottom | *C. palustris* | 18 June 2011 JP & P. Koutecký |
| C038-11 | 2 | 8.69 | Czech Republic, Strakonice, town distr. Podskalí, blind arm on right bank of Otava River in camp | 49°15'26.2"N, 13°53'19.8"E | 390 | pool | *C. ×vigens* | 19 June 2011 JP & P. Koutecký |
| C040-11 | 3 | 8.83 | Poland, prov. Małopolskie, Jabłonka, Czarna Orawa River WSW of village | 49°28'07.8"N, 19°40'22.8"E | 604 | river (sand) |  | 25 June 2011 JP & ZK |
| C045-11 | 8 | **8.88±0.04** | Slovakia, distr. Námestovo, Rabča, Modré jazierko pool close to Polhoranka stream near S edge of village | 49°27'32.7"N, 19°29'50.1"E | 620 | pool |  | 24 June 2011 JP & ZK |
| C007-12 | 3 | 9.11 | Czech Republic, distr. Mělník, Vrbno, pool on left side of Vltava River ENE of village | 50°19'41.1"N, 14°28'34.5"E | 160 | pool |  | 13 May 2012 JP |
| C014-12 | 1 | 8.89 | Czech Republic, distr. Rakovník, Řevničov, puddle on forest path N of Horní Kracle fishpond | 50°8'35.7"N, 13°50'30.4"E | 410 | puddle | *C. stagnalis* | 12 June 2012 JP & A. Potůčková |
| C015-12 | 4 | 8.78 | Czech Republic, distr. Rakovník, Třtice, littoral zone of small pond close to solitude Třtická Lísa | 50°9'49.6"N, 13°50'20.4"E | 450 | exposed bottom | *C. cophocarpa* | 12 June 2012 JP & A. Potůčková |
| C022-12 | 3 | 8.82 | Czech Republic, distr. Mladá Boleslav, Dolení Kruhy, Jizera River NNW of village | 50°33'45.6"N, 14°59'41.3"E | 230 | river (sand) | *C. ×vigens* | 14 June 2012 JP & PT |
| C026-12 | 1 | 8.72 | Czech Republic, distr. Český Krumlov, Vyšší Brod, Vltava River E of town | 48°37'15.6"N, 14°19'38.1"E | 550 | river (sand) |  | 19 July 2012 JP |
| C027-12 | 1 | 8.82 | Czech Republic, distr. Český Krumlov, Hrudkov, Vltava River SE of village | 48°37'36.9"N, 14°21'20.4"E | 540 | river (sand) |  | 19 July 2012 JP |
| C029-12 | 1 | 8.90 | Czech Republic, Český Krumlov, Vltava River on E edge of town | 48°48'49.3"N, 14°19'47.0"E | 495 | river (sand) |  | 21 July 2012 JP |
| C035-12 | 2 | 8.90 | Denmark, Jutland, reg. Nordjylland, Trængstrup, Sonderupå stream near bridge of Suldrupvej road | 56°51'08.5"N, 09°38'11.3"E | 18 | stream (sand) | *C. platycarpa* | 1 Aug. 2012 JP & ZK |
| C039-12 | 1 | 9.27 | Denmark, Jutland, reg. Midtjylland, Nees, Søndersund lake | 56°23'57.0"N, 08°13'51.3"E | 0 | lake | *C. platycarpa, C. ×vigens* | 1 Aug. 2012 JP & ZK |
| C043-12 | 3 | 8.98 | Denmark, Jutland, reg. Midtjylland, Silkeborg, small pond between Lupinvej and Borgergade streets | 56°10'57.8"N, 09°32'53.5"E | 29 | fishpond |  | 1 Aug. 2012 JP & ZK |
| C045-12 | 1 | **9.10±0.09** | Denmark, Jutland, reg. Midtjylland, Tarm, Sonderå River WNW of town | 55°55'06.4"N, 08°28'58.0"E | 1 | river (mud) | *C. platycarpa* | 3 Aug. 2012 JP & ZK |
| C047-12 | 1 | 9.22 | Denmark, Jutland, reg. Syddanmark, Tange, Varde Ǻ River NE of town | 55°38'39.2"N, 08°32'37.7"E | 4 | river (mud) |  | 3 Aug. 2012 JP & ZK |
| C050-12 | 1 | 9.20 | Germany, Lower Saxony, Baven, Örtze River below the bridge of Zur Örtze road | 52°50'41.9"N, 10°6'00.7"E | 52 | river (mud) | *C. platycarpa* | 3 Aug. 2012 JP & ZK |
| C058-12 | 16 | 8.98 | Czech Republic, Ústí nad Orlicí, Tichá Orlice River under railway bridge | 49°58'17.0"N, 16°23'05.4"E | 320 | river (sand) |  | 15 Aug. 2012 JP |
| C059-12 | 15 | 9.03 | Czech Republic, Ústí nad Orlicí, town distr. Kerhartice, Tichá Orlice River above bridge of Sokolská street | 49°58'19.6"N, 16°21'44.0"E | 315 | river (sand) |  | 15 Aug. 2012 JP |
| C060-12 | 12 | 9.02 | Czech Republic, Ústí nad Orlicí, town distr. Kerhartice, Tichá Orlice River W of bridge of Sokolská street | 49°58'22.2"N, 16°21'24.6"E | 315 | river (sand) |  | 15 Aug. 2012 JP |
| C062-12 | 26 | **8.85±0.09** | Czech Republic, distr. Ústí nad Orlicí, Brandýs nad Orlicí, Tichá Orlice River below bridge of Žerotínova street *(resampled for chromosome counting as C094-13, 1 IX 2013 JP & KK)* | 50°0'4.9"N, 16°16'45.1"E | 295 | river (sand) | *C. cophocarpa, C. hamulata × C. cophocarpa?* | 15 Aug. 2012 JP |
| C064-12 | 5 | **8.78±0.08** | Czech Republic, distr. Ústí nad Orlicí, Choceň, Tichá Orlice River on SW edge of town | 49°59'43.2"N, 16°12'36.1"E | 270 | river (sand) | *C. hamulata × C. cophocarpa?* | 15 Aug. 2012 JP |
| C067-12 | 10 | **8.91±0.05** | Czech Republic, distr. Ústí nad Orlicí, Choceň, millrace of Tichá Orlice River on NW edge of town | 50°0'4.5"N, 16°12'13.2"E | 270 | stream (sand) | *C. hamulata × C. cophocarpa?* | 15 Aug. 2012 JP |
| C069-12 | 1 | 8.94 | Czech Republic, distr. Prachatice, Lenora, Teplá Vltava River below bridge in village | 48°55'39.1"N, 13°47'41.1"E | 765 | stream (sand) |  | 14 Aug. 2012 M. Hrdinová |
| C071-12 | 1 | 8.65 | Czech Republic, distr. Prachatice, Pěkná, Vltava River W of village | 48°51'9.7"N, 13°55'11.4"E | 730 | river (sand) |  | 15 Aug. 2012 M. Hrdinová |
| C072-12 | 1 | 8.84 | Czech Republic, distr. Prachatice, Ovesná, Vltava River ESE of railway station | 48°48'18.5"N, 13°56'47.2"E | 725 | river (sand) |  | 15 Aug. 2012 M. Hrdinová |
| C073-12 | 4 | 8.83 | Czech Republic, Liberec, Lužická Nisa River in city centre | 50°46'01.1"N, 15°03'10.2"E | 350 | stream (sand) |  | 26 Aug. 2012 JP |
| C078-12 | 4 | 9.04 | Czech Republic, distr. Liberec, Arnoltice, small blind arm of Bulovský stream close road bridge in village | 50°57'47.4"N, 15°05'47.8"E | 290 | pool |  | 11 Oct. 2012 JP |
| C079-12 | 3 | 8.85 | Czech Republic, distr. Liberec, Horní Pertoltice, small pond SSE of village | 50°58'30.7"N, 15°05'43.2"E | 289 | reservoir |  | 11 Oct. 2012 JP |
| C080-12 | 1 | 8.94 | Czech Republic, distr. Liberec, Horní Pertoltice, Šálkův fishpond | 50°59'01.5"N, 15°06'12.5"E | 319 | fishpond | *C. palustris* | 11 Oct. 2012 JP |
| C087-12 | 1 | 8.75 | Czech Republic, distr. Svitavy, Jedlová, Pulec fishpond | 49°39'37.5"N, 16°20'15.2"E | 584 | exposed bottom | *C. palustris* | 16 Oct. 2012 JP |
| C091-12 | 1 | 8.86 | Czech Republic, distr. Domažlice, Stráž, Zelenovský fishpond | 49°24'36.8"N, 12°53'27.6"E | 480 | fishpond |  | 29 Oct. 2013 JP |
| C014-13 | 3 | **8.88±0.06** | Czech Republic, distr. Břeclav, Kostice, pools in forest Tvrdonické polesí SE of village | 48°44'14.0"N, 17°00'21.9"E | 150 | pool | *C. cophocarpa* | 4 June 2013 JP & M. Hrdinová |
| C022-13 | 1 | **8.84±0.05** | Czech Republic, distr. Příbram, Mokrovraty, Dolní (Tušimský) fishpond | 49°48'23.7"N, 14°13'50.6"E | 380 | fishpond | *C. cophocarpa* | 11 June 2013 JP & KK |
| C025-13 | 1 | 8.86 | Czech Republic, distr. Žďár nad Sázavou, Švařec, Svratka River in S part of village | 49°31'03.0"N, 16°20'31.5"E | 350 | river (sand) |  | 20 June 2013 JP & ZK |
| C028-13 | 2 | **8.91±0.04** | Czech Republic, distr. Olomouc, Nové Zámky, old separated arm of Morava River in locality Pod Templem | 49°43'14.8"N, 17°01'01.4"E | 240 | pool | *C. cophocarpa* | 21 June 2013 JP & ZK |
| C033-13 | 1 | 8.74 | Czech Republic, distr. Jindřichův Hradec, Lomy, fishpond in central part of village | 49°6'39.6"N, 15°9'28.2"E | 570 | fishpond |  | 25 June 2013 S. Píšová |
| C044-13 | 3 | 8.79 | Czech Republic, distr. Trutnov, Kohoutov, Rabiš fishpond | 50°27'6.5"N, 15°53'14.2"E | 470 | fishpond | *C. palustris* | 4 July 2013 JP |
| C047-13 | 5 | 8.88 | Czech Republic, distr. Trutnov, Choustníkovo Hradiště, Hradišťský stream in village | 50°25'34.6"N, 15°52'42.0"E | 284 | stream (sand) |  | 4 July 2013 JP |
| C049-13 | 1 | **8.86±0.01** | USA, Oregon, Multnomah County, Troutdale, Sandy River Delta Park, old separated arm of Delta River | 45°33'20.3"N, 122°22'0.1"W | 4 | pool |  | 25 July 2013 JP & T. Kávová |
| C050-13 | 1 | **8.86±0.02** | USA, Oregon, Lane County, Eugene, canal by bridge of Seavey Loop Road in city | 44°0'41.8"N, 123°0'19.1"W | 139 | stream (mud) |  | 28 July 2013 JP & T. Kávová |
| C051-13 | 1 | **8.85±0.07** | USA, Oregon, Lane County, Florence, Woahink Lake | 43°55'41.7"N, 124°5'53.3"W | 15 | lake |  | 28 July 2013 JP & T. Kávová |
| C052-13 | 2 | 8.97 | USA, Oregon, Curry County, Langlois, Floras Lake | 42°54'9.0"N, 124°30'9.3"W | 4 | lake |  | 29 July 2013 JP & T. Kávová |
| C058-13 | 1 | **8.73±0.05** | Czech Republic, distr. Cheb, Milhostov, Lužní stream on S edge of locality Děvín | 50°9'30.8"N, 12°25'41.5"E | 440 | stream (sand) |  | 19 July 2013 KK & JarR |
| C059-13 | 1 | 8.76 | Czech Republic, distr. Cheb, Milhostov, Lužní stream near S edge of locality Děvín | 50°9'25"N, 12°25'45"E | 440 | stream (sand) |  | 19 July 2013 KK & JarR |
| C060-13 | 1 | 8.67 | Czech Republic, distr. Cheb, Milhostov, Lužní stream WSW of village | 50°9'12"N, 12°25'43"E | 439 | stream (sand) |  | 19 July 2013 KK & JarR |
| C061-13 | 1 | 8.67 | Czech Republic, distr. Cheb, Povodí, Lužní stream on confluence with Sázek stream | 50°8'57.4"N, 12°25'27.2"E | 436 | stream (sand) | *C. stagnalis* | 19 July 2013 KK & JarR |
| C064-13 | 1 | 8.75 | Czech Republic, distr. Cheb, Milhostov, small pond WSW of village | 50°8'59.6"N, 12°25'33.1"E | 438 | reservoir |  | 18 July 2013 KK & JarR |
| C065-13 | 7 | **8.81±0.04** | Czech Republic, distr. Cheb, Dvorek, Stodolský stream by road bridge NE of village | 50°8'19.5"N, 12°24'42.5"E | 431 | stream (sand) | *C. cophocarpa, C. stagnalis* | 19 July 2013 KK & JarR |
| C069-13b | 1 | 8.93 | Czech Republic, distr. Domažlice, Nemanice, blind arm of Nemanický stream SW of settlement Nemaničky | 49°25'48.6"N, 12°42'40.6"E | 504 | pool | *C. platycarpa, C. ×vigens* | 10 Aug. 2013 M. Hrdinová |
| C080-13 | 2 | **8.89±0.10** | Czech Republic, distr. Jablonec nad Nisou, Bedřichov, Blatný rybník reservoir | 50°48'36.9"N, 15°9'53.8"E | 770 | reservoir | *C. cophocarpa* | 22 Aug. 2013 JP & KK |
| C084-13 | 1 | 8.91 | Czech Republic, distr. Klatovy, Horská Kvilda, Hamerský stream SW of village | 49°3'15.6"N, 13°33'17.7"E | 1030 | stream (sand) |  | 25 Aug. 2013 JP & KK |
| C086-13 | 1 | 8.95 | Czech Republic, distr. Cheb, Prameny, Dlouhá stoka canal NE of village | 50°03'43.2"N, 12°44'43.7"E | 780 | stream (sand) |  | 21 Aug. 2013 J. Chrtek jr. |
| C090-13 | 19 | **8.80±0.04** | Czech Republic, distr. Ústí nad Orlicí, Orlické Podhůří, Tichá Orlice River by railway bridge near settlement Luh | 49°59'34.0"N, 16°20'9.5"E | 315 | river (sand) |  | 1 Sept. 2013 JP & KK |
| C091-13 | 28 | **8.82±0.04** | Czech Republic, distr. Ústí nad Orlicí, Orlické Podhůří, Tichá Orlice River under bridge in settlement Bezpráví | 49°59'55.6"N, 16°20'1.2"E | 315 | river (sand) |  | 1 Sept. 2013 JP & KK |
| C096-13 | 1 | 8.85 | Czech Republic, distr. Ústí nad Orlicí, Choceň, town distr. Peliny, confluence of outlet of separated river arm to Tichá Orlice River | 49°59'55.7"N, 16°14'17.4"E | 295 | river (mud) | *C. cophocarpa* | 1 Sept. 2013 JP & KK |
| C097-13 | 1 | 8.98 | Czech Republic, distr. Cheb, Jindřichov, Ohře River near village | 50°6'24"N, 12°23'47"E | 435 | river (sand) |  | 24 Aug. 2013 JanR & JarR |
| C098-13 | 1 | 8.95 | Czech Republic, distr. Cheb, Tršnice, Ohře River near village | 50°6'50"N, 12°24'6"E | 435 | river (sand) |  | 24 Aug. 2013 JanR & JarR |
| C099-13 | 1 | 8.90 | Czech Republic, distr. Cheb, Vokov, Ohře River ESE of settlement Třídvoří | 50°6'54"N, 12°26'39"E | 425 | river (sand) |  | 24 Aug. 2013 JanR & JarR |
| C100-13 | 1 | 8.84 | Czech Republic, distr. Cheb, Nebanice, Ohře River near village | 50°6'41"N, 12°28'12"E | 420 | river (sand) |  | 24 Aug. 2013 JanR & JarR |
| C101-13 | 1 | 8.68 | Czech Republic, distr. Cheb, Dobroše, Ohře River near village | 50°6'44"N, 12°31'3"E | 415 | river (sand) |  | 24 Aug. 2013 JanR & JarR |
| C102-13 | 1 | 8.88 | Czech Republic, distr. Sokolov, Liboc, Ohře River near village | 50°7'13"N, 12°31'11"E | 415 | river (sand) |  | 24 Aug. 2013 JanR & JarR |
| C103-13 | 1 | 8.71 | Czech Republic, distr. Cheb, Dobroše, pool on right bank of Ohře River N of village | 50°6'43"N, 12°30'55"E | 415 | pool |  | 24 Aug. 2013 JanR & JarR |
| C104-13 | 1 | 8.89 | Czech Republic, distr. Sokolov, Dasnice, Ohře River in village | 50°8'46"N, 12°33'56.0"E | 410 | river (sand) |  | 25 Aug. 2013 JanR & JarR |
| C105-13 | 1 | 8.79 | Czech Republic, distr. Sokolov, Šabina, Ohře River in village | 50°8'5"N, 12°34'45"E | 410 | river (sand) |  | 25 Aug. 2013 JanR & JarR |
| C106-13 | 1 | 8.88 | Czech Republic, Sokolov, Ohře River in town | 50°11'0"N, 12°38'31"E | 400 | river (sand) |  | 25 Aug. 2013 JanR & JarR |
| C107-13 | 1 | 8.75 | Czech Republic, distr. Karlovy Vary, Dalovice, Ohře River near village | 50°14'35"N, 12°53'36"E | 370 | river (sand) |  | 29 Aug. 2013 JanR & JarR |
| C109-13 | 1 | 8.97 | Czech Republic, distr. Karlovy Vary, Sedlečko, blind arm of Ohře River NNE of settlement Hubertus | 50°14'27"N, 12°55'49"E | 365 | pool | *C. ×vigens* | 29 Aug. 2013 JanR & JarR |
| C111-13 | 1 | 8.82 | Czech Republic, distr. Ústí nad Labem, Ostrov, fishpond close to hotel Ostrov | 50°48'15"N, 14°2'49"E | 450 | fishpond |  | 28 Aug. 2013 JanR & JarR |
| C112-13 | 1 | 8.76 | Czech Republic, distr. Ústí nad Labem, Ostrov, fishpond above hotel Ostrov | 50°48'13"N, 14°2'51"E | 450 | fishpond |  | 28 Aug. 2013 JanR & JarR |
| C113-13 | 1 | 8.92 | Czech Republic, distr. Ústí nad Labem, Petrovice, fishpond on Olšový stream E of village | 50°48'5"N, 13°59'32"E | 475 | fishpond |  | 27 Aug. 2013 JanR & JarR |
| C117-13 | 1 | **8.92±0.01** | Czech Republic, distr. Ústí nad Labem, Ostrov, Ostrovský fishpond | 50°48'20"N, 14°2'47"E | 445 | fishpond |  | 28 Aug. 2013 JanR & JarR |
| C118-13 | 1 | 8.82 | Czech Republic, distr. Děčín, Maxičky, Rybníček fishpond | 50°48'29"N, 14°10'55"E | 435 | fishpond |  | 26 Aug. 2013 JanR & JarR |
| C120-13 | 1 | 8.78 | Czech Republic, distr. Ústí nad Labem, Libouchec, upper fishpond in locality Libouchecké rybníčky | 50°45'59"N, 14°3'34"E | 330 | fishpond |  | 27 Aug. 2013 JanR & JarR |
| C129-13 | 5 | 8.88 | Czech Republic, distr. Jihlava, Řásná, Velký Pařezitý fishpond | 49°13'41.4"N, 15°22'44.5"E | 675 | fishpond | *C. cophocarpa* | 8 Sept. 2013 JP |
| C132-13b | 1 | 8.81 | Germany, Bayern, Hohenfels, Follerenbach stream between Hohenfels and Kalmünz villages | 49°11'7.2"N, 11°54'45.7"E | 364 | stream (sand) | *C. ×vigens* | 7 Sept. 2013 KK |
| C133-13 | 2 | 8.92 | Czech Republic, distr. Praha-východ, Kostelec nad Černými Lesy, puddle on forest path SSW of town | 49°58'13.6"N, 14°50'47.7"E | 390 | puddle | *C. stagnalis* | 9 Sept. 2013 JP |
| C144-13 | 1 | 8.81 | Czech Republic, distr. Olomouc, Domašov nad Bystřicí, Bystřice stream in village | 49°44'30"N, 17°26'47.0"E | 495 | stream (sand) |  | 25 Sept. 2013 JarR |
| C146-13 | 1 | 8.90 | Czech Republic, distr. Trutnov, Arnultovice, on forest path ENE of village | 50°33'24"N, 15°44'23"E | 465 | puddle |  | 25 Sept. 2013 JanR |
| C14-001 | 1 | **8.67±0.09** | Hungary, Zala county, Csesztreg, „Holt-Kerka-rivulet“ in village | 46°42'59"N, 16°30'46"E | 180 | stream (sand) |  | 24 Apr. 2014 A. Mesterházy |
| ***C. hermaphroditica* L.** | | | | | | | | |
|  |  |  |  |  |  |  |  |  |
| C097-09 | 1 | **1.92±0.02** | Czech Republic, Institute of Botany ASCR in Třeboň, cultivated (probably originates from Eastern Bohemia) |  |  |  |  | 23 Sept. 2009 JP |
| C088-12 | 2 | **1.94±0.01** | Czech Republic, distr. Svitavy, Jedlová, Ráček I pond | 49°40'7.0"N, 16°19'59.5"E | 595 | reservoir |  | 11 Oct. 2012 JP |
| C089-12 | 3 | **1.94±0.02** | Czech Republic, distr. Svitavy, Jedlová, Ráček III pond | 49°40'7.3"N, 16°19'53.7"E | 595 | reservoir |  | 11 Oct. 2012 JP |
| C090-12 | 1 | **1.93±0.01** | Czech Republic, distr. Svitavy, Jedlová, Ráček II pond | 49°40'14.3"N, 16°19'52.5"E | 595 | reservoir |  | 11 Oct. 2012 JP |
| C127-13 | 1 | **2.00±0.02** | Sweden, prov. Östergötland, Tranås, Lake Sommen, part Torpasjön, Stora Bianäset | 57°58'49.4"N, 15°6'55.0"E | 153 | lake |  | 25 Aug. 2013 A. Svenson |
| ***C. lenisulca* Clavaud** | | | | | | | | |
|  |  |  |  |  |  |  |  |  |
| C004-13 | 1 | **3.59±0.02** | Italy, prov. Siena, Chiusi, ditch near road Strada Provinciale 300, N of town | 43°2'51.0"N, 11°56'8.4"E | 250 | ditch |  | 23 May 2013 PT & B. Kubátová |
| C005-13 | 1 | **3.58±0.04** | Italy, prov. Ravenna, Ravenna, Bardello area, pool E of Strade Statale Romea road | 44°32'26.2"N, 12°14'19.3"E | -1 | pool |  | 24 May 2013 PT & B. Kubátová |
| C006-13 | 1 | **3.61±0.01** | Italy, prov. Ravenna, Ravenna, ditch in fields near road Via Bevano, S of city | 44°16'37.2"N, 12°14'29.5"E | 1 | ditch |  | 23 May 2013 PT & B. Kubátová |
| ***C. obtusangula* Le Gall** | | | | | | | | |
|  |  |  |  |  |  |  |  |  |
| C034-12 | 5 | **3.87±0.02** | Denmark, Jutland, reg. Syddanmark, Varming, drainage ditch near Ribeå River E of town | 55°19'17.5"N, 08°50'09.9"E | 1 | ditch |  | 31 July 2012 JP & ZK |
| C048-12 | 1 | **3.88±0.02** | Denmark, Jutland, reg. Syddanmark, Højer, Højer Havn, system of drainage ditches SSW of village | 54°56'16.2"N, 08°41'12.1"E | 0 | ditch |  | 3 Aug. 2012 JP & ZK |
| C049-12 | 20 | **3.85±0.02** | Germany, Lower Saxony, Garstedt, Luhe River below bridge of Vierhöfener Straße | 53°16'47.1"N, 10°10'59.7"E | 16 | river (sand) |  | 3 Aug. 2012 JP & ZK |
| C052-12 | 1 | **3.85±0.04** | Netherlands, prov. Noord-Brabant, Breda, Turfvaart canal on SW edge of town | 51°33'50.1"N, 4°44'3.3"E | 30 | stream (mud) |  | 1 Aug. 2012 PT & B. Kubátová |
| C053-12 | 1 | **3.88±0.02** | Belgium, Flemish Region, Retie, canal below bridge of Postelsebaan street | 51°16'38.4"N, 5°9'2.7"E | 30 | stream (mud) |  | 31 July 2012 PT & B. Kubátová |
| C007-13 | 1 | **3.76±0.00** | Italy, prov. Rieti, Colli sul Velino, race of Fiume Velino River near Hotel Relais Villa D'Assio | 42°29'30.0"N, 12°48'7.1"E | 379 | stream (sand) |  | 23 May 2013 PT & B. Kubátová |
| C008-13 | 1 | **3.71±0.01** | Italy, prov. Salerno, Sala Consilina, ditch near Strada Provinciale 11i Road | 40°22'5.2"N, 15°35'58.6"E | 453 | ditch |  | 21 May 2013 PT & B. Kubátová |
| C009-13 | 1 | **3.73±0.02** | Italy, prov. Salerno, Sala Consilina, stream near Strada Provinciale 11i road | 40°22'1.9"N, 15°35'53.3"E | 453 | stream (sand) |  | 21 May 2013 PT & B. Kubátová |
| ***C. palustris* L.** | | | | | | | | |
|  |  |  |  |  |  |  |  |  |
| C006-07 | 1 | 3.87 | Czech Republic, distr. Jindřichův Hradec, Lomnice nad Lužnicí, Velký Lomnický fishpond | 49°5'33"N, 14°42'35"E | 415 | exposed bottom |  | 27 June 2007 JP & ZK |
| C018-13 | 1 | 3.91 | Czech Republic, distr. Rakovník, Lužná, fishpond near solitude Krásná dolina | 50°7'26.7"N, 13°47'48.3"E | 370 | fishpond |  | 15 Aug. 2007 JP |
| C037-07 | 1 | 3.91 | Czech Republic, distr. Klatovy, Hartmanice, ruts on path S of solitude Dobrá Voda | 49°8'43.7"N, 13°26'8.1"E | 940 | puddle |  | 1 July 2007 JP |
| C008-08 | 1 | **3.86±0.05** | Czech Republic, distr. Chrudim, Nasavrky, Horní fishpond | 49°50'23.5"N, 15°47'57.6"E | 520 | fishpond |  | 23 May 2008 JP |
| C017-08a | 1 | 3.93 | Czech Republic, distr. Břeclav, ruts on forest path NE of chateau Pohansko | 48°44'12"N, 16°54'20"E | 155 | puddle | *C. cophocarpa* | 11 June 2008 JP |
| C022-08 | 2 | **3.88±0.03** | Czech Republic, distr. Břeclav, Lanžhot, puddles on forest path NE of town | 48°43'56"N, 16°59'30"E | 145 | puddle |  | 12 June 2008 JP |
| C028-08 | 1 | 3.91 | Czech Republic, distr. Jindřichův Hradec, Třeboň, alder carr by Kaprový fishpond | 48°59'28"N, 14°46'59"E | 435 | puddle |  | 10 July 2008 JP |
| C035-08 | 1 | 3.93 | Czech Republic, distr. Jindřichův Hradec, Žíteč, Černá Cepáků fishpond | 49°0'39"N, 14°53'30"E | 445 | fishpond |  | 11 July 2008 JP |
| C036-08 | 2 | **3.90±0.03** | Czech Republic, distr. Jindřichův Hradec, Lužnice, Namšal fishpond | 49°3'26.5"N, 14°45'20.8"E | 425 | exposed bottom |  | 12 July 2008 JP |
| C038-08 | 3 | 3.91 | Czech Republic, distr. Jindřichův Hradec, Lomnice nad Lužnicí, Malý Lomnický fishpond | 49°5'35"N, 14°43'1"E | 425 | fishpond |  | 12 July 2008 JP |
| C042-08 | 1 | 3.93 | Czech Republic, distr. Praha-východ, Jevany, ruts on side of path E of village | 49°58'19"N, 14°51'5"E | 370 | puddle | *C. stagnalis* | 19 July 2008 JP |
| C045-08 | 1 | 3.88 | Czech Republic, distr. Praha-východ, Jevany, middle forest fishpond in Aldašín deer-park | 49°58'6.6"N, 14°50'21.2"E | 385 | exposed bottom | *C. hamulata, C. stagnalis* | 19 July 2008 JP |
| C054-08 | 1 | 3.86 | Czech Republic, distr. Cheb, Podílná, puddle on forest path WNW of Čtvrtý fishpond | 50°8'52"N, 12°13'7"E | 550 | puddle |  | 2 Aug. 2008 JP |
| C055-08 | 1 | 3.85 | Czech Republic, distr. Cheb, Podílná, puddle on forest path NE of Čtvrtý fishpond | 50°8'56"N, 12°13'27"E | 575 | puddle | *C. hamulata* | 2 Aug. 2008 JP |
| C073-08 | 1 | 3.83 | Czech Republic, distr. Prachatice, Volary, forest path SE of town | 48°53'43"N, 13°52'47"E | 755 | puddle |  | 6 Sept. 2008 JP |
| C075-08 | 1 | 3.92 | Czech Republic, distr. Prachatice, Volary, lower fishpond on Jedlový stream in Stögrova Huť settlement | 48°54'52.4"N, 13°51'19.1"E | 795 | fishpond | *C. cophocarpa* | 6 Sept. 2008 JP |
| C082-08 | 1 | 3.89 | Czech Republic, distr. Prachatice, Stožec, puddle on forest path near rock formation Gabrielstein | 48°49'49"N, 13°51'3"E | 895 | puddle |  | 7 Sept. 2008 JP |
| C098-08 | 1 | 3.95 | Czech Republic, distr. Praha-východ, Kytín, pudle on crossroads of forest paths WSW of village | 49°50'48.0"N, 14°11'51.0"E | 470 | puddle | *C. stagnalis* | 6 Nov. 2008 JP |
| C009-09 | 1 | 3.87 | Czech Republic, distr. České Budějovice, Koloděje nad Lužnicí, fishpond by road on S edge of village | 49°14'50"N, 14°25'13"E | 375 | exposed bottom |  | 16 May 2009 JP |
| C014-09 | 1 | **3.93±0.03** | Czech Republic, distr. Praha-východ, Podolanka, biggest fishpond on SE edge of village | 50°9'18.1"N, 14°36'3.7"E | 220 | exposed bottom |  | 24 May 2009 JP |
| C021-09 | 1 | 3.83 | Czech Republic, distr. Česká Lípa, Hradčany, Hradčanské rybníky area, puddle on forest path near Strážovský fishpond | 50°36'31"N, 14°44'30"E | 275 | puddle |  | 8 June 2009 JP |
| C025-09 | 1 | 3.93 | Czech Republic, distr. Česká Lípa, Rybnov, alluvium of Bobří stream between Jílovka and Velká Komora fishponds | 50°37'21"N, 14°30'40"E | 260 | exposed bottom |  | 9 June 2009 JP |
| C029-09 | 1 | **3.93±0.02** | Czech Republic, distr. Litoměřice, Děkovka, puddle on forest path NE of hill Hrádek | 50°29'29.1"N, 13°55'10.6"E | 495 | puddle |  | 17 June 2009 JP |
| C037-09 | 1 | 3.90 | Czech Republic, distr. Karlovy Vary, Přestání, on intersection of forest paths W of village | 50°4'25.1"N, 13°4'18.7"E | 610 | puddle | *C. stagnalis* | 6 Aug. 2009 JP |
| C042-09 | 1 | 3.92 | Czech Republic, distr. Karlovy Vary, Toužim, Velká Studna fishpond | 50°3'28.1"N, 12°59'58.0"E | 615 | fishpond |  | 6 Aug. 2009 JP |
| C043-09 | 2 | 3.93 | Czech Republic, distr. Karviná, Dolní Marklovice, small fishpond close to state border | 49°53'7.3"N, 18°34'4.6"E | 227 | fishpond | *C. cophocarpa* | 9 Aug. 2009 JP |
| C048-09 | 1 | **3.91±0.03** | Czech Republic, distr. Karviná, Prstná, on intersection of paths N of settlement Kempy | 49°54'38.5"N, 18°34'9.4"E | 270 | puddle |  | 9 Aug. 2009 JP |
| C074-09 | 1 | 3.96 | Czech Republic, distr. Prostějov, Šubířov, puddle on forest path E of village | 49°36'1.0"N, 16°49'53.1"E | 601 | puddle |  | 24 Aug. 2009 JP |
| C079-09 | 1 | 3.92 | Czech Republic, distr. Blansko, Benešov, puddle on intersection of forest paths WNW of hill Skalky | 49°30'8.5"N, 16°47'2.3"E | 710 | puddle | *C. cophocarpa* | 25 Aug. 2009 JP |
| C082-09 | 1 | 3.92 | Czech Republic, distr. Blansko, Žďárná, ruts on forest path SE of village | 49°26'58.3"N, 16°47'10.2"E | 575 | puddle | *C. cophocarpa, C. stagnalis* | 26 Aug. 2009 JP |
| C100-09 | 2 | **3.87±0.04** | Czech Republic, distr. Pardubice, Dolní Jelení, Oborecký fishpond | 50°2'29.6"N, 16°7'14.4"E | 280 | exposed bottom | *C. hamulata* | 8 Oct. 2009 JP |
| C105-09 | 1 | **3.89±0.04** | Czech Republic, distr. Svitavy, Opatov, marsh below dam of Nový fishpond | 49°50'28.7"N, 16°29'24.2"E | 423 | exposed bottom |  | 19 Oct. 2009 JP |
| C106-09 | 1 | 3.92 | Czech Republic, distr. Svitavy, Opatov, puddle on side of forest path S of railway station Semanín | 49°50'17.2"N, 16°28'17.2"E | 430 | puddle | *C. cophocarpa, C. hamulata, C. stagnalis* | 19 Oct. 2009 JP |
| C002-10 | 1 | **3.87±0.04** | Czech Republic, distr. Příbram, Obořiště, Přívaží fishpond | 49°45'14.2"N, 14°8'48.5"E | 398 | exposed bottom |  | 8 May 2010 JP |
| C009-10 | 1 | **3.87±0.03** | Czech Republic, distr. Teplice, Duchcov, fishpond Hájská I | 50°36'51.4"N, 13°43'32.2"E | 230 | exposed bottom |  | 1 June 2010 JP |
| C015-10 | 1 | **3.90±0.02** | Czech Republic, distr. Semily, Turnov, small ponds below castle Hrubý Rohozec | 50°35'58.6"N, 15°9'35.6"E | 246 | reservoir | *C. hamulata, C. platycarpa* | 12 June 2010 JP |
| C025-10 | 1 | 3.86 | Czech Republic, distr. Jičín, Lukavec u Hořic, puddle on forest path E of Zákopský fishpond | 50°24'40.6"N, 15°37'8.8"E | 336 | puddle | *C. stagnalis* | 26 June 2010 JP |
| C027-10 | 1 | 3.88 | Czech Republic, distr. Zlín, Zbožensko, puddle on forest path NNW of village | 49°14'33.4"N, 17°36'48.4"E | 265 | puddle |  | 30 June 2010 JP |
| C029-10 | 1 | 3.92 | Czech Republic, Zlín, city distr. Mladcová, puddle on forest path S of Zadní vrch hill | 49°15'30.2"N, 17°38'9.7"E | 360 | puddle |  | 30 June 2010 JP |
| C030-10 | 1 | **3.85±0.05** | Czech Republic, Zlín, city distr. Zlínské Paseky, puddle on forest path NW of settlement Mezihoří | 49°15'17.6"N, 17°39'29.5"E | 343 | puddle |  | 30 June 2010 JP |
| C031-10 | 1 | 3.94 | Czech Republic, Zlín, city distr. Zlínské Paseky, puddle on side of forest road N of settlement Kocanda | 49°15'9.5"N, 17°40'22.4"E | 330 | puddle |  | 30 June 2010 JP |
| C033-10 | 1 | 3.91 | Czech Republic, distr. Zlín, Lukov, Bezedník fishpond | 49°17'59.1"N, 17°43'26.4"E | 320 | fishpond | *C. cophocarpa* | 1 July 2010 JP |
| C038-10 | 1 | 3.96 | Czech Republic, distr. Zlín, Vlčková, puddle on forest path ENE of hill Bzová | 49°19'16.5"N, 17°44'27.1"E | 563 | puddle | *C. stagnalis* | 1 July 2010 JP |
| C041-10 | 1 | **3.96±0.02** | Czech Republic, distr. Zlín, Vlčková, puddle on intersection of forest paths NNE of hill Ondřejovsko | 49°19'39.3"N, 17°43'43.7"E | 560 | puddle | *C. stagnalis* | 1 July 2010 JP |
| C058-10 | 1 | 3.96 | Czech Republic, distr. Domažlice, Novosedly, ruts on forest path SSW of castle ruins Starý Herštejn | 49°27'29.5"N, 12°42'28.7"E | 560 | puddle | *C. stagnalis* | 6 July 2010 JP & P. Koutecký |
| C065-10 | 1 | 3.91 | Czech Republic, distr. Domažlice, Pila, Hadrovec fishpond | 49°24'48.4"N, 12°52'5.5"E | 465 | fishpond |  | 9 July 2010 JP & M. Štech |
| C098-10 | 1 | 3.91 | Czech Republic, distr. Česká Lípa, Velký Valtinov, puddle on path ESE of Zaječí fishpond | 50°45'7.6"N, 14°43'30.6"E | 325 | puddle | *C. platycarpa*, *C. stagnalis* | 28 July 2010 JP |
| C101-10 | 1 | 3.89 | Czech Republic, distr. Česká Lípa, Kunratice u Cvikova, ruts on forest path SW of Kamenáč hill | 50°45'27.0"N, 14°42'27.3"E | 333 | puddle | *C. platycarpa*, *C. stagnalis* | 28 July 2010 JP |
| C131-10 | 1 | 3.90 | Czech Republic, distr. Znojmo, Olbramkostel, puddles along forest path SE of castle ruins Šimperk | 48°55'33.2"N, 15°55'17.2"E | 387 | puddle | *C. stagnalis* | 16 Aug. 2010 JP |
| C138-10 | 1 | 3.88 | Czech Republic, distr. Chrudim, Rohozná, Hubský fishpond | 49°48'27.2"N, 15°49'22.0"E | 575 | exposed bottom | *C. cophocarpa, C. hamulata* | 27 Aug. 2010 JP & ZK |
| C140-10 | 1 | 3.93 | Czech Republic, distr. Havlíčkův Brod, Kocourov, puddles on dam of Návesní fishpond | 49°45'13.7"N, 15°48'1.4"E | 553 | puddle | *C. stagnalis* | 28 Aug. 2010 JP & ZK |
| C144-10 | 2 | 3.88 | Czech Republic, distr. Havlíčkův Brod, Stružinec, Januš fishpond | 49°44'11.8"N, 15°50'43.5"E | 548 | exposed bottom | *C. hamulata* | 28 Aug. 2010 JP & ZK |
| C158-10 | 1 | **3.95±0.04** | Czech Republic, distr. Tábor, Hamr, Smíchov II fishpond | 49°9'36.7"N, 14°46'41.7"E | 420 | exposed bottom | *C. cophocarpa* | 20 Sept. 2010 S. Píšová |
| C006-11 | 2 | 3.91 | Czech Republic, distr. Nymburk, Loučeň, puddle on forest path E of spring Dobrá Voda | 50°18'02.7"N, 15°02'07.3"E | 264 | puddle | *C. stagnalis* | 30 May 2011 JP |
| C024-11 | 1 | **3.87±0.03** | Czech Republic, distr. Klatovy, Horažďovice, on alluvium in blind arm of Otava River on SW edge of town | 49°19'16.1"N, 13°41'29.0"E | 420 | exposed bottom |  | 17 June 2011 JP & P. Koutecký |
| C034-11 | 1 | 3.85 | Czech Republic, distr. Strakonice, Katovice, on alluvium of Otava River below weir in village | 49°16'20.7"N, 13°49'30.7"E | 400 | exposed bottom | *C. hamulata* | 18 June 2011 JP & P. Koutecký |
| C042-11 | 2 | **3.90±0.03** | Slovakia, distr. Námestovo, Zubrohlava, exposed bottom on bank of Orava reservoir | 49°25'06.4"N, 19°30'50.9"E | 595 | exposed bottom |  | 24 June 2011 JP & ZK |
| C044-11 | 2 | 3.90 | Slovakia, distr. Námestovo, Bobrov, exposed bottom on bank of Orava reservoir | 49°24'48.2"N, 19°31'47.4"E | 595 | exposed bottom |  | 24 June 2011 JP & ZK |
| C046-11 | 1 | 3.88 | Slovakia, distr. Námestovo, Mútňanská Píla, meadow ditch near NW edge of village | 49°28'06.8"N, 19°17'28.3"E | 785 | exposed bottom |  | 25 June 2011 JP & ZK |
| C060-11 | 1 | 3.88 | Czech Republic, distr. Třebíč, Číměř, on bank of water reservoir Dalešice ENE of village | 49°12'03.5"N, 16°00'27.0"E | 385 | exposed bottom |  | 7 July 2011 P. Koutecký |
| C019-12 | 1 | 3.91 | Czech Republic, distr. Mladá Boleslav, Březina, Oběšenec fishpond | 50°32'20.2"N, 15°3'36.4"E | 238 | fishpond |  | 14 June 2012 JP & PT |
| C023-12 | 1 | 3.85 | Slovakia, distr. Zlaté Moravce, Mankovce, Mankovce reservoir | 48°25'11.0"N, 18°19'55.0"E | 230 | reservoir |  | 4 July 2012 JP |
| C024-12 | 2 | 3.93 | Slovakia, distr. Zlaté Moravce, Jelenec, on N bank of Jelenec fishpond | 48°24'4.1"N, 18°12'13.6"E | 234 | exposed bottom | *C. cophocarpa* | 4 July 2012 JP |
| C081-12 | 1 | **3.85±0.01** | Czech Republic, distr. Liberec, Horní Pertoltice, Šálkův fishpond | 50°59'01.5"N, 15°06'12.5"E | 319 | fishpond | *C. hamulata* | 11 Oct. 2012 JP |
| C082-12 | 1 | 3.85 | Czech Republic, distr. Liberec, Dolní Pertoltice, puddle of path on forest edge NNW of village | 50°59'07.4"N, 15°03'49.5"E | 290 | puddle |  | 11 Oct. 2012 JP |
| C086-12 | 1 | 3.93 | Czech Republic, distr. Svitavy, Jedlová, Pulec fishpond | 49°39'37.5"N, 16°20'15.2"E | 584 | exposed bottom | *C. hamulata* | 16 Oct. 2012 JP |
| C098-12 | 1 | 3.91 | Czech Republic, distr. Svitavy, Jedlová, small pool in Zlatý stream in village near Dubovec fishpond | 49°39'21.4"N, 16°19'49.6"E | 580 | pool |  | 16 Oct. 2012 JP |
| C021-13 | 1 | 3.89 | Czech Republic, distr. Příbram, Mokrovraty, puddle on meadow path NE of hill Dolní Jezberná | 49°48'43.8"N, 14°14'44.7"E | 335 | puddle | *C. stagnalis* | 11 June 2013 JP & KK |
| C032-13 | 1 | 3.85 | Czech Republic, distr. Jindřichův Hradec, Lomy, fishpond in W part of village | 49°06'39.1"N, 15°09'24.5"E | 570 | exposed bottom |  | 25 June 2013 S. Píšová |
| C034-13 | 1 | 3.81 | Czech Republic, distr. Jindřichův Hradec, Nová Olešná, puddles on meadow near NE bank of Zadní Obecní fishpond | 49°09'49.7"N, 15°09'37.6"E | 554 | puddle |  | 27 June 2013 ZK |
| C043-13 | 1 | 3.80 | Czech Republic, distr. Trutnov, Kocbeře, puddle on forest path between village and Rabiš fishpond | 50°27'09.2"N, 15°52'42.4"E | 470 | puddle | *C. stagnalis* | 4 July 2013 JP |
| C045-13 | 2 | 3.85 | Czech Republic, distr. Trutnov, Kohoutov, Rabiš fishpond | 50°27'6.5"N, 15°53'14.2"E | 470 | fishpond | *C. hamulata* | 4 July 2013 JP |
| C048-13 | 1 | **3.96±0.00** | USA, Washington, Snohomish County, Cathcart, lake S of Shadow Lake near Shohomish River | 47°51'33.8"N, 122°5'30.3"W | 6 | lake |  | 15 July 2013 JP & T. Kávová |
| C073-13 | 1 | **3.94±0.04** | Norway, prov. Sør-Trøndelag, Hjerkinn/Kongsvoll/Drivdalen Protection Area, path near Gåvålivatnet lake | 62°16'26.3"N, 9°37'29.1"E | 941 | puddle |  | 4 Aug. 2013 KK |
| C140-13 | 2 | 3.82 | Czech Republic, distr. Frýdek-Místek, Horní Lomná, puddle on forest path near observation tower Kozubová | 49°34'13.1"N, 18°40'24.7"E | 975 | puddle | *C. cophocarpa* | 16 Sept. 2013 JP & KK |
| C150-13 | 1 | 3.93 | Sweden, prov. Lappland, Karesuando, Munionjoki River on N edge of village | 68°26'33"N, 22°28'54"E | 320 | river (mud) |  | 6 Aug. 2013 ZK |
| ***C. platycarpa* Kütz.** | | | | | | | | |
|  |  |  |  |  |  |  |  |  |
| C065-08 | 5 | **6.18±0.02** | Czech Republic, distr. Liberec, Kunratice, small retention reservoir E of village | 50°55'31.4"N, 15°0'44.8"E | 300 | reservoir |  | 19 Aug. 2008 JP |
| C067-08 | 3 | **6.16±0.04** | Czech Republic, distr. Liberec, Višňová, Višňovský stream in village | 50°57'59.3"N, 15°1'32.3"E | 235 | stream (mud) |  | 19 Aug. 2008 JP |
| C068-08 | 1 | **6.15±0.05** | Czech Republic, distr. Liberec, Černousy, Smědá stream S of railway station | 51°0'6"N, 15°2'16"E | 220 | stream (mud) | *C. hamulata* | 19 Aug. 2008 JP |
| C001-09 | 1 | **6.17±0.05** | Czech Republic, distr. Louny, Stradonice, side arm of Debeřský stream | 50°22'21"N, 13°57'48"E | 200 | stream (mud) |  | 25 Apr. 2009 JP |
| C023-09 | 2 | **6.21±0.06** | Czech Republic, distr. Česká Lípa, Břehyně, Břehyně-Pecopala area, ruts on path E of Břehyňský fishpond | 50°34'43"N, 14°43'42"E | 275 | puddle | *C. cophocarpa* | 8 June 2009 JP |
| C057-09 | 2 | **6.19±0.02** | Czech Republic, distr. Děčín, Mikulášovice, Mikulášovický stream in NW part of town | 50°58'50.7"N, 14°20'36.7"E | 385 | stream (mud) | *C. hamulata* | 18 Aug. 2009 JP |
| C062-09 | 3 | **6.26±0.06** | Czech Republic, distr. Děčín, Mikulášovice, ditch on E part of town | 50°57'36.7"N, 14°23'28.0"E | 455 | ditch |  | 18 Aug. 2009 JP |
| C063-09 | 2 | **6.18±0.02** | Czech Republic, distr. Děčín, Velký Šenov, settlement Leopoldka, ruts on forest patk SSE of Černý fishpond | 50°58'43.7"N, 14°23'33.4"E | 395 | puddle |  | 18 Aug. 2009 JP |
| C065-09 | 2 | **6.22±0.07** | Czech Republic, distr. Děčín, Velký Šenov, Vilémovský stream in town | 50°59'24.3"N, 14°22'36.4"E | 350 | stream (mud) | *C. hamulata* | 18 Aug. 2009 JP |
| C066-09 | 1 | **6.22±0.05** | Czech Republic, distr. Děčín, Velký Šenov, slough beside path NNE of Partyzánský vrch hill | 51°0'29.3"N, 14°24'50.2"E | 385 | puddle |  | 19 Aug. 2009 JP |
| C070-09 | 5 | **6.18±0.02** | Czech Republic, distr. Děčín, Šluknov, Stříbrný stream in town | 51°0'19.9"N, 14°27'5.7"E | 340 | stream (sand) | *C. hamulata* | 19 Aug. 2009 JP |
| C008-10 | 2 | 6.11 | Czech Republic, distr. Teplice, Duchcov, outfall from storage ponds to fishpond Hranáč | 50°36'43.0"N, 13°43'57.0"E | 226 | ditch |  | 1 June 2010 JP |
| C010-10 | 2 | **6.15±0.05** | Czech Republic, distr. Teplice, Háj u Duchcova, Hájský stream near Salát fishpond | 50°37'5.2"N, 13°43'14.2"E | 234 | stream (mud) |  | 1 June 2010 JP |
| C013-10 | 4 | **6.16±0.06** | Czech Republic, distr. Semily, Turnov, small ponds below castle Hrubý Rohozec | 50°35'58.6"N, 15°9'35.6"E | 246 | reservoir | *C. hamulata, C. palustris* | 12 June 2010 JP |
| C018-10 | 3 | 6.18 | Czech Republic, distr. Semily, Mašov, fishpond in village | 50°34'19.6"N, 15°8'43.0"E | 257 | fishpond |  | 12 June 2010 JP |
| C057-10 | 2 | 6.09 | Czech Republic, distr. Domažlice, Novosedly, rivulet in alder carr NNE of village | 49°27'25.1"N, 12°41'56.2"E | 533 | stream (mud) |  | 6 July 2010 JP & P. Koutecký |
| C062-10 | 3 | 6.12 | Czech Republic, distr. Domažlice, Babylon, millrace of Teplá Bystřice stream close to railway station | 49°23'54.4"N, 12°52'9.2"E | 483 | stream (mud) |  | 9 July 2010 JP & M. Štech |
| C063-10 | 12 | **6.20±0.03** | Czech Republic, distr. Domažlice, Babylon, Babylon fishpond | 49°23'58.4"N, 12°51'53.2"E | 465 | fishpond |  | 9 July 2010 JP & M. Štech |
| C074-10 | 2 | 6.14 | Czech Republic, distr. Domažlice, Novosedly, Nemanický stream NW of village | 49°26'46.7"N, 12°42'10.1"E | 510 | stream (sand) |  | 7 July 2010 ZK |
| C087-10 | 2 | 6.23 | Czech Republic, distr. Cheb, Odrava, ditch near Ohře River NW of village | 50°6'27.1"N, 12°28'29.6"E | 420 | ditch |  | 19 July 2010 JP & ZK |
| C092-10 | 4 | 6.15 | Czech Republic, distr. Liberec, Jablonné v Podještědí, Panenský stream between Pivovarský and Markvartický fishponds | 50°46'33.1"N, 14°46'54.1"E | 310 | stream (mud) | *C. hamulata* | 28 July 2010 JP |
| C097-10 | 1 | **6.27±0.02** | Czech Republic, distr. Česká Lípa, Velký Valtinov, puddle on path ESE of Zaječí fishpond | 50°45'7.6"N, 14°43'30.6"E | 325 | puddle | *C. palustris*, *C. stagnalis* | 28 July 2010 JP |
| C100-10 | 1 | 6.18 | Czech Republic, distr. Česká Lípa, Kunratice u Cvikova, ruts on forest path SW of Kamenáč hill | 50°45'27.0"N, 14°42'27.3"E | 333 | puddle | *C. palustris*, *C. stagnalis* | 28 July 2010 JP |
| C103-10 | 3 | 6.17 | Czech Republic, distr. Česká Lípa, Radvanec, Dobranovský stream close to estuary to Radvanecký fishpond | 50°44'59.2"N, 14°35'43.7"E | 294 | stream (mud) | *C. hamulata* | 28 July 2010 JP |
| C105-10 | 3 | 6.19 | Czech Republic, distr. Česká Lípa, Sloup v Čechách, Dobranovský stream in village | 50°44'34.6"N, 14°35'32.2"E | 290 | stream (mud) | *C. hamulata* | 29 July 2010 JP |
| C108-10 | 2 | 6.14 | Czech Republic, distr. Česká Lípa, Horní Kamenice, small reservoir on little stream N of village | 50°48'31.3"N, 14°26'43.3"E | 355 | pool |  | 29 July 2010 JP |
| C109-08 | 1 | 6.24 | Czech Republic, distr. Česká Lípa, Kamenická Nová Víska, bank of Kamenice stream NNE of village | 50°48'5.2"N, 14°22'48.1"E | 250 | exposed bottom |  | 29 July 2010 JP |
| C111-10 | 2 | **6.19±0.02** | Czech Republic, distr. Česká Lípa, Janská, flooded depression under rock overhang in gorge of Olešnička stream S of village | 50°48'0.8"N, 14°21'43.1"E | 245 | pool |  | 29 July 2010 JP |
| C150-10 | 2 | 6.19 | Czech Republic, distr. Děčín, Šluknov, Spréva (Spree) stream on state border in former village Fukov | 51°2'37"N, 14°30'15"E | 300 | stream (sand) | *C. hamulata* | 9 Sept. 2010 P. Bauer |
| C010-12 | 1 | 6.23 | Czech Republic, distr. Most, Louka u Litvínova, on S bank of Plutovský fishpond | 50°35'24.2"N, 13°37'57.3"E | 295 | fishpond |  | 27 May 2012 JP |
| C011-12 | 3 | 6.22 | Czech Republic, distr. Most, Mariánské Radčice, Radčický stream E of village | 50°34'18.9"N, 13°40'45.8"E | 250 | stream (mud) |  | 27 May 2012 JP |
| C032-12 | 3 | 6.30 | Denmark, Jutland, Hajstrup, reg. Syddanmark, Grønå stream above bridge of Slogsherredsvej road | 54°56'00.6"N, 09°06'53.7"E | 12 | stream (mud) |  | 31 July 2012 JP & ZK |
| C036-12 | 2 | 6.24 | Denmark, Jutland, reg. Nordjylland, Trængstrup, Sonderupå stream near bridge of Suldrupvej road | 56°51'08.5"N, 09°38'11.3"E | 18 | stream (sand) | *C. hamulata* | 1 Aug. 2012 JP & ZK |
| C037-12 | 1 | 6.19 | Denmark, Jutland, reg. Nordjylland, Bislev, Binderupå stream near the bridge of Jordemodervej road | 56°56'14.8"N, 09°38'15.7"E | 22 | stream (sand) |  | 1 Aug. 2012 JP & ZK |
| C040-12 | 3 | 6.25 | Denmark, Jutland, reg. Midtjylland, Nees, Søndersund lake | 56°23'57.0"N, 08°13'51.3"E | 0 | lake | *C. hamulata, C. ×vigens* | 1 Aug. 2012 JP & ZK |
| C042-12 | 2 | 6.11 | Denmark, Jutland, reg. Midtjylland, Nees, Indfjorden lake | 56°24'38.3"N, 08°13'50.0"E | 0 | lake |  | 1 Aug. 2012 JP & ZK |
| C044-12 | 1 | **6.24±0.04** | Denmark, Jutland, reg. Midtjylland, Tarm, Sonderå River WNW of town | 55°55'06.4"N, 08°28'58.0"E | 1 | river (mud) | *C. hamulata* | 3 Aug. 2012 JP & ZK |
| C046-12 | 2 | **6.29±0.03** | Denmark, Jutland, reg. Syddanmark, Tange, ditch between Varde Ǻ River and Lundvej Road NE of town | 55°38'41.1"N, 08°32'35.7"E | 6 | ditch |  | 3 Aug. 2012 JP & ZK |
| C051-12 | 3 | 6.21 | Germany, Lower Saxony, Baven, Örtze River below the bridge of Zur Örtze road | 52°50'41.9"N, 10°6'00.7"E | 52 | river (mud) | *C. hamulata* | 3 Aug. 2012 JP & ZK |
| C054-12 | 1 | 6.22 | Germany, Niedersachsen, Nordstemmen, Leine River below bridge of Reichsstraße street | 52°8'50.1"N, 9°46'3.9"E | 76 | stream (mud) |  | 3 Aug. 2012 PT & B. Kubátová |
| C077-12 | 1 | 6.11 | Germany, Bayern, Potterstein, Weihersbach stream near Gasthof Schüttersmühle | 49°45'5.2"N, 11°25'37.9"E | 411 | stream (mud) |  | 10 Sept. 2012 A. Knotek |
| C096-12 | 2 | 6.20 | Czech Republic, distr. Děčín, Šluknov, puddle on forest path in forest complex between town distr. Království and former settlement Fukov | 51°1'19.8"N, 14°29'41.9"E | 380 | puddle |  | 31 Dec. 2012 JP |
| C097-12 | 1 | 6.19 | Czech Republic, distr. Děčín, Šluknov, puddle on crossroads of paths near cemetery of former settlement Fukov | 51°2'32.9"N, 14°30'6.9"E | 310 | puddle |  | 31 Dec. 2012 JP |
| C010-13 | 1 | **6.06±0.01** | Italy, prov. Cosenza, Acri, ditch by waterlogged meadow near Strada Statale 660 road, N of Lago di Cecita | 39°26'18.5"N, 16°31'29.2"E | 1207 | ditch |  | 20 May 2013 PT & B. Kubátová |
| C069-13a | 2 | 6.27 | Czech Republic, distr. Domažlice, Nemanice, blind arm of Nemanický stream SW of settlement Nemaničky | 49°25'48.6"N, 12°42'40.6"E | 504 | pool | *C. hamulata, C. ×vigens* | 10 Aug. 2013 M. Hrdinová |
| C070-13 | 1 | 6.24 | Czech Republic, distr. Domažlice, Novosedly, Nemanický stream SW of village | 49°26'21.4"N, 12°42'27.4"E | 510 | stream (sand) |  | 11 Aug. 2013 M. Hrdinová |
| C079-13 | 3 | **6.11±0.01** | Czech Republic, distr. Liberec, Černousy, stream along second uppermost fishpond in pond cascade E of settlement V Poli | 50°59'35.3"N, 15°03'26.4"E | 242 | stream (mud) | *C. stagnalis* | 22 Aug. 2013 JP & KK |
| C122-13 | 1 | **6.16±0.00** | Czech Republic, distr. Děčín, Zadní Ves, pool opposite restaurant Hraniční Bouda in village | 50°47'51"N, 14°5'0,5"E | 560 | pool |  | 28 Aug. 2013 JanR & JarR |
| C124-13 | 3 | **6.10±0.02** | Czech Republic, distr. Děčín, Zadní Ves, Oborský fishpond | 50°48'21"N, 14°4'20"E | 525 | fishpond |  | 28 Aug. 2013 JanR & JarR |
| ***C. stagnalis* Scop.** | | | | | | | | |
|  |  |  |  |  |  |  |  |  |
| C008-07 | 1 | 3.01 | Czech Republic, distr. Jindřichův Hradec, Horusice, locality Ruda, puddle on path near Horusický fishpond | 49°9'8"N, 14°41'22"E | 415 | puddle |  | 28 June 2007 JP & ZK |
| C014-07 | 1 | 2.99 | Czech Republic, distr. Příbram, Čenkov, Běštínský stream below spring | 49°47'23"N, 14°1'41"E | 550 | stream (mud) |  | 29 July 2007 JP |
| C019-07 | 1 | 2.93 | Czech Republic, distr. Rakovník, Lužná, puddle on forest path near solitude Krásná dolina | 50°7'42"N, 13°48'3"E | 385 | puddle |  | 15 Aug. 2007 JP |
| C020-07 | 1 | 3.01 | Czech Republic, distr. Rakovník, Řevničov, locality Prameny Klíčavy, puddle on forest path | 50°8'38.2"N, 13°49'37.2"E | 420 | puddle | *C. hamulata* | 16 Aug. 2007 JP |
| C024-07 | 1 | 2.99 | Czech Republic, distr. Kladno, Lány, forest puddle near solitude Nový Dvůr | 50°8'57"N, 13°55'37"E | 425 | puddle | *C. hamulata* | 16 Aug. 2007 JP |
| C027-07 | 1 | 2.95 | Czech Republic, distr. Kladno, Běleč, forest terrain depression near solitude Fialka | 50°3'47.5"N, 13°57'55.9"E | 395 | puddle |  | 16 Aug. 2007 JP |
| C030-07 | 1 | 3.03 | Czech Republic, distr. Mladá Boleslav, Jabkenice, puddle on forest path near Erichův fishpond | 50°18'58"N, 15°2'46"E | 250 | puddle |  | 22 Aug. 2007 JP |
| C005-08 | 1 | **3.02±0.01** | Czech Republic, Praha, city distr. Zbraslav, locality Krňák, Lipanský stream | 49°58'23"N, 14°22'29"E | 195 | stream (mud) | *C. cophocarpa* | 15 May 2008 JP |
| C007-08 | 1 | **3.02±0.02** | Czech Republic, distr. Chrudim, Kochánovice, puddle on forest path near Hluboký fishpond | 49°54'2"N, 15°47'17.8"E | 330 | puddle |  | 23 May 2008 JP |
| C026-08 | 1 | 3.01 | Czech Republic, distr. Děčín, Mezná, on forest path E of village | 50°52'0"N, 14°19'17"E | 265 | puddle |  | 3 July 2008 JP |
| C033-08 | 1 | 3.04 | Czech Republic, distr. Jindřichův Hradec, Lutová, ruts on path near Safl fishpond | 49°0'11"N, 14°53'9"E | 440 | puddle |  | 11 July 2008 JP |
| C034-08 | 1 | 3.00 | Czech Republic, distr. Jindřichův Hradec, Lutová, puddle on forest path near Humlenský fishpond | 49°0'2"N, 14°52'29"E | 440 | puddle |  | 11 July 2008 JP |
| C039-08 | 1 | 3.02 | Czech Republic, distr. Praha-východ, Krupá, ruts on forest path WSW of village | 50°0'56"N, 14°51'46"E | 275 | puddle |  | 19 July 2008 JP |
| C040-08 | 1 | 3.02 | Czech Republic, distr. Praha-východ, Svatbín, puddle on forest path in forest Brník | 49°59'27"N, 14°53'35"E | 370 | puddle |  | 19 July 2008 JP |
| C041-08 | 2 | **2.98±0.03** | Czech Republic, distr. Praha-východ, Svatbín, ditch along forest path in forest Brník | 49°59'19"N, 14°53'35"E | 370 | puddle |  | 19 July 2008 JP |
| C043-08 | 1 | 3.03 | Czech Republic, distr. Praha-východ, Jevany, ruts on side of path E of village | 49°58'19"N, 14°51'5"E | 370 | puddle | *C. palustris* | 19 July 2008 JP |
| C046-08 | 1 | **3.03±0.00** | Czech Republic, distr. Praha-východ, Jevany, middle forest fishpond in Aldašín deer-park | 49°58'6.6"N, 14°50'21.2"E | 385 | exposed bottom | *C. hamulata, C. palustris* | 19 July 2008 JP |
| C050-08 | 1 | 2.95 | Czech Republic, distr. Cheb, Františkovy Lázně, settlement Lužná, puddle on forest path near Vražedný fishpond | 50°6'19"N, 12°17'16"E | 450 | puddle | *C. hamulata* | 1 Aug. 2008 JP |
| C053-08 | 2 | **3.00±0.01** | Czech Republic, distr. Cheb, Libá, puddle on forest path NW of Kamenný fishpond | 50°8'13"N, 12°13'7"E | 540 | puddle |  | 2 Aug. 2008 JP |
| C057-08 | 1 | 2.99 | Czech Republic, distr. Cheb, Mýtinka, puddle on forest path E of village | 50°8'47"N, 12°19'4"E | 505 | puddle |  | 2 Aug. 2008 JP |
| C066-08 | 1 | 3.00 | Czech Republic, distr. Liberec, Víska, ditch along road in village | 50°56'51"N, 15°1'45.0"E | 240 | ditch |  | 19 Aug. 2008 JP |
| C074-08 | 1 | 2.93 | Czech Republic, distr. Prachatice, Volary, ruts on forest path in railway triangle SE of town | 48°53'27"N, 13°52'11"E | 750 | puddle |  | 6 Sept. 2008 JP |
| C087-08 | 1 | 2.99 | Czech Republic, distr. Žďár nad Sázavou, Karlov, puddle on forest path near Velké Dářko fishpond | 49°38'47.9"N, 15°53'45.4"E | 615 | puddle |  | 22 Sept. 2008 JP |
| C095-08 | 1 | 3.03 | Czech Republic, distr. Příbram, Voznice, puddle on path close to N edge of Charvátův fishpond | 49°48'58"N, 14°11'37"E | 415 | puddle |  | 6 Nov. 2008 JP |
| C097-08 | 1 | 3.01 | Czech Republic, distr. Příbram, Chouzavá, ruts on forest path W of hill Točná | 49°50'12"N, 14°11'42"E | 420 | puddle | *C. cophocarpa* | 6 Nov. 2008 JP |
| C099-08 | 1 | 2.99 | Czech Republic, distr. Praha-východ, Kytín, pudle on crossroads of forest paths WSW of village | 49°50'48.0"N, 14°11'51.0"E | 470 | puddle | *C. palustris* | 6 Nov. 2008 JP |
| C104-08 | 1 | 2.99 | Czech Republic, distr. Mladá Boleslav, Bělá pod Bezdězem, village distr. Vrchbělá, puddle on forest path | 50°31'17"N, 14°46'38"E | 315 | puddle | *C. hamulata* | 28 Sept. 2008 J. Suda |
| C106-08 | 1 | 3.01 | Czech Republic, distr. Louny, Domoušice, on path NNE of railway station Mutějovice | 50°13'20.1"N, 13°42'23.9"E | 520 | puddle |  | 29 Sept. 2008 H. Chudáčková |
| C107-08 | 1 | 3.01 | Czech Republic, distr. Brno-venkov, Moravské Knínice, puddle on forest path SW of village | 49°16'43.1"N, 16°29'19.7"E | 327 | puddle |  | 2 June 2008 H. Chudáčková |
| C024-09 | 1 | **3.02±0.03** | Czech Republic, distr. Česká Lípa, Doksy, Okenský stream in town | 50°34'1"N, 14°39'20"E | 270 | stream (mud) |  | 9 June 2009 JP |
| C034-09 | 1 | 2.99 | Czech Republic, distr. Karlovy Vary, Údrč, on forest path near Malý Pásmovský fishpond | 50°7'37.9"N, 13°6'34.7"E | 625 | puddle |  | 5 Aug. 2009 JP |
| C035-09 | 1 | 2.97 | Czech Republic, distr. Karlovy Vary, Veselov, puddle on side of path NE of hill Havraní vrch | 50°7'23.5"N, 13°6'49.4"E | 645 | puddle |  | 5 Aug. 2009 JP |
| C036-09 | 1 | 2.98 | Czech Republic, distr. Karlovy Vary, Přestání, on intersection of forest paths W of village | 50°4'25.1"N, 13°4'18.7"E | 610 | puddle | *C. palustris* | 6 Aug. 2009 JP |
| C038-09 | 1 | **2.98±0.01** | Czech Republic, distr. Karlovy Vary, Přestání, puddle on forest path W of village | 50°4'21.8"N, 13°4'22.7"E | 600 | puddle |  | 6 Aug. 2009 JP |
| C039-09 | 1 | 2.99 | Czech Republic, distr. Karlovy Vary, Radyně, puddle on forest path NNE of village | 50°4'22.7"N, 13°3'20.3"E | 610 | puddle |  | 6 Aug. 2009 JP |
| C041-09 | 1 | 2.95 | Czech Republic, distr. Karlovy Vary, Radyně, puddle on path NNW of village | 50°4'17.2"N, 13°1'57.5"E | 615 | puddle |  | 6 Aug. 2009 JP |
| C049-09 | 1 | 2.98 | Czech Republic, distr. Karviná, Prstná, ruts on forest path SE of village | 49°54'41.1"N, 18°34'4.1"E | 270 | puddle |  | 9 Aug. 2009 JP |
| C053-09 | 1 | **2.99±0.03** | Czech Republic, distr. Nový Jičín, Hukovice, ruts on intersection of forest paths in forest Roveň | 49°37'35.7"N, 18°2'39.0"E | 275 | puddle |  | 11 Aug. 2009 JP |
| C055-09 | 1 | 2.97 | Czech Republic, distr. Nový Jičín, Libhošť, puddle on intersection of forest path in forest Roveň | 49°36'59.0"N, 18°2'43.0"E | 285 | puddle |  | 11 Aug. 2009 JP |
| C056-09 | 1 | 2.97 | Czech Republic, distr. Nový Jičín, Libhošť, puddle beside main forest road in forest Roveň | 49°36'41.5"N, 18°2'49.9"E | 295 | puddle |  | 11 Aug. 2009 JP |
| C067-09 | 1 | **2.99±0.01** | Czech Republic, distr. Děčín, Velký Šenov, ruts on forest path SSE of Špičák hill | 51°0'39.8"N, 14°24'35.6"E | 385 | puddle |  | 19 Aug. 2009 JP |
| C068-09 | 1 | 3.01 | Czech Republic, distr. Děčín, Šluknov, puddle beside forest path WSW of settlement Nové Hraběcí | 51°0'51.1"N, 14°25'0.0"E | 410 | puddle |  | 19 Aug. 2009 JP |
| C077-09 | 1 | 2.96 | Czech Republic, distr. Blansko, Benešov, puddle on forest path SE of village | 49°30'15.4"N, 16°46'50.5"E | 700 | puddle |  | 25 Aug. 2009 JP |
| C081-09 | 1 | 3.00 | Czech Republic, distr. Blansko, Žďárná, ruts on forest path SE of village | 49°26'58.3"N, 16°47'10.2"E | 575 | puddle | *C. cophocarpa, C. palustris* | 26 Aug. 2009 JP |
| C098-09 | 2 | 2.99 | Czech Republic, distr. Pardubice, Horní Jelení, ditch of Čermná stream on E edge of town | 50°2'55.6"N, 16°5'41.5"E | 285 | ditch |  | 8 Oct. 2009 JP |
| C103-09 | 2 | **3.01±0.01** | Czech Republic, distr. Pardubice, Rousínov, side arm of Čermná stream close to Horní Pecák fishpond | 50°2'57.1"N, 16°7'54.4"E | 275 | ditch | *C. cophocarpa* | 8 Oct. 2009 JP |
| C107-09 | 1 | 3.03 | Czech Republic, distr. Svitavy, Opatov, puddle on side of forest path S of railway station Semanín | 49°50'17.2"N, 16°28'17.2"E | 430 | puddle | *C. cophocarpa, C. hamulata, C. palustris* | 19 Oct. 2009 JP |
| C110-09 | 1 | 2.99 | Czech Republic, distr. Svitavy, Opatov, puddle on forest path NNW of pond Rebelant | 49°50'2.7"N, 16°28'19.4"E | 437 | puddle | *C. hamulata* | 19 Oct. 2009 JP |
| C113-09 | 1 | 3.04 | Czech Republic, distr. Svitavy, Opatov, ruts on forest path NW of pond Rebelant | 49°49'53.5"N, 16°28'20.0"E | 440 | puddle | *C. hamulata* | 19 Oct. 2009 JP |
| C017-10 | 2 | **3.00±0.01** | Czech Republic, distr. Semily, Kacanovy, puddle on forest path SW of Valdštejn castle | 50°33'31.1"N, 15°9'46.5"E | 345 | puddle |  | 12 June 2010 JP |
| C020-10 | 1 | 2.99 | Czech Republic, distr. Děčín, Mikulášovice, meadow spring SE of town | 50°57'1"N, 14°22'41"E | 450 | puddle |  | 8 June 2010 J. Hadinec & H. Härtel |
| C024-10 | 1 | 3.02 | Czech Republic, distr. Jičín, Lukavec u Hořic, puddle on forest path E of Zákopský fishpond | 50°24'40.6"N, 15°37'8.8"E | 336 | puddle | *C. palustris* | 26 June 2010 JP |
| C035-10 | 1 | **3.02±0.02** | Czech Republic, distr. Zlín, Lukov, ditch along forest path N of Bezedník fishpond | 49°18'23.2"N, 17°43'32.9"E | 360 | puddle |  | 1 July 2010 JP |
| C037-10 | 1 | **2.95±0.01** | Czech Republic, distr. Zlín, Lukov, puddle on forest path NNE of hill Velá | 49°18'57.8"N, 17°43'32.0"E | 455 | puddle |  | 1 July 2010 JP |
| C039-10 | 1 | 2.97 | Czech Republic, distr. Zlín, Vlčková, puddle on forest path ENE of hill Bzová | 49°19'16.5"N, 17°44'27.1"E | 563 | puddle | *C. palustris* | 1 July 2010 JP |
| C040-10 | 1 | 2.99 | Czech Republic, distr. Zlín, Vlčková, puddle on intersection of forest paths NNE of hill Ondřejovsko | 49°19'39.3"N, 17°43'43.7"E | 560 | puddle | *C. palustris* | 1 July 2010 JP |
| C043-10 | 1 | 2.96 | Czech Republic, distr. Zlín, Vlčková, puddle on forest path NE of hill Ondřejovsko | 49°19'52.6"N, 17°44'6.2"E | 570 | puddle | *C. cophocarpa* | 1 July 2010 JP |
| C049-10 | 1 | 2.96 | Czech Republic, distr. Tachov, Racov, locality Racovské rybníčky, place digged up by forest animals | 49°37'37.1"N, 12°51'9.7"E | 495 | puddle |  | 5 July 2010 JP & ZK |
| C059-10 | 1 | 3.03 | Czech Republic, distr. Domažlice, Novosedly, ruts on forest path SSW of castle ruins Starý Herštejn | 49°27'29.5"N, 12°42'28.7"E | 560 | puddle | *C. palustris* | 6 July 2010 JP & P. Koutecký |
| C061-10 | 1 | 3.00 | Czech Republic, distr. Plzeň-jih, Zemětice, artificial pool E of Dražský fishpond | 49°34'54.0"N, 13°12'8.5"E | 385 | pool |  | 8 July 2010 JP & J. Chrtek |
| C088-10 | 1 | 3.01 | Czech Republic, distr. Plzeň-sever, Hůrky, on forest path N of village | 49°53'37.7"N, 13°11'4.6"E | 542 | puddle |  | 19 July 2010 JP & ZK |
| C096-10 | 1 | 2.97 | Czech Republic, distr. Česká Lípa, Velký Valtinov, puddle on path ESE of Zaječí fishpond | 50°45'7.6"N, 14°43'30.6"E | 325 | puddle | *C. palustris*, *C. platycarpa* | 28 July 2010 JP |
| C099-10 | 1 | 2.96 | Czech Republic, distr. Česká Lípa, Kunratice u Cvikova, ruts on forest path SW of Kamenáč hill | 50°45'27.0"N, 14°42'27.3"E | 333 | puddle | *C. palustris*, *C. platycarpa* | 28 July 2010 JP |
| C107-10 | 1 | **2.99±0.03** | Czech Republic, distr. Česká Lípa, Horní Kamenice, puddle on forest path N of village | 50°48'24.0"N, 14°26'47.5"E | 355 | puddle |  | 29 July 2010 JP |
| C124-10 | 1 | 2.96 | Czech Republic, distr. Znojmo, Lesná, puddle on forest path SSW of villa Lusthaus | 48°53'37.6"N, 15°51'13.5"E | 475 | puddle |  | 15 Aug. 2010 JP |
| C125-10 | 3 | 2.98 | Czech Republic, distr. Znojmo, Čížov, right bank of Dyje River S of village | 48°51'38.0"N, 15°52'27.8"E | 280 | river (mud) |  | 16 Aug. 2010 JP |
| C126-10 | 1 | 2.96 | Czech Republic, distr. Znojmo, Čížov, puddle on forest path E of village | 48°52'46.0"N, 15°52'55.1"E | 425 | puddle |  | 16 Aug. 2010 JP |
| C128-10 | 1 | **2.99±0.02** | Czech Republic, distr. Znojmo, Šumná, puddle on forest path SSW of castle ruins Šimperk | 48°55'29.0"N, 15°54'28.1"E | 395 | puddle |  | 16 Aug. 2010 JP |
| C132-10 | 1 | 2.99 | Czech Republic, distr. Znojmo, Olbramkostel, puddles along forest path SE of castle ruins Šimperk | 48°55'33.2"N, 15°55'17.2"E | 387 | puddle | *C. palustris* | 16 Aug. 2010 JP |
| C141-10 | 1 | 2.97 | Czech Republic, distr. Havlíčkův Brod, Kocourov, puddles on dam of Návesní fishpond | 49°45'13.7"N, 15°48'1.4"E | 553 | puddle | *C. palustris* | 28 Aug. 2010 JP & ZK |
| C152-10 | 3 | **2.97±0.03** | Czech Republic, distr. Prachatice, Lipka, small fishpond in settlement Stará Lipka | 49°1'29.1"N, 13°43'57.1"E | 875 | fishpond |  | 25 Sept. 2010 JP |
| C005-11 | 2 | 2.99 | Czech Republic, distr. Nymburk, Loučeň, puddle on forest path E of spring Dobrá Voda | 50°18'02.7"N, 15°02'07.3"E | 264 | puddle | *C. palustris* | 30 May 2011 JP |
| C007-11 | 1 | **3.00±0.03** | Czech Republic, distr. Nymburk, Chudíř, on forest path near SW bank of Nový fishpond | 50°17'58.0"N, 15°00'45.6"E | 230 | puddle |  | 30 May 2011 JP |
| C061-11 | 1 | 2.99 | Czech Republic, distr. Třebíč, Jackov, puddle on forest path NW of Hrachovec fishpond | 49°2'56.4"N, 15°44'5.7"E | 470 | puddle |  | 8 July 2011 JP |
| C013-12 | 1 | 2.97 | Czech Republic, distr. Rakovník, Řevničov, puddle on forest path N of Horní Kracle fishpond | 50°8'35.7"N, 13°50'30.4"E | 410 | puddle | *C. hamulata* | 12 June 2012 JP & A. Potůčková |
| C030-12 | 1 | 2.97 | Czech Republic, distr. Sokolov, Rotava, puddle on forest path near rock formation Rotavské varhany | 50°18'12"N, 12°34'30"E | 600 | puddle |  | 29 July 2012 J. Hadinec |
| C038-12 | 1 | 3.05 | Denmark, Jutland, reg. Midtjylland, Nees, pasture near between Søndersund lake and Ulfsundvej road | 56°23'57.2"N, 08°13'56.8"E | 0 | puddle |  | 1 Aug. 2012 JP & ZK |
| C075-12 | 1 | **2.95±0.03** | Czech Republic, distr. Ústí nad Labem, Petrovice, puddle on path near former settlement Hladov | 50°48'45"N, 13°59'45"E | 480 | puddle |  | 13 Sept. 2012 J. Hadinec & P. Bauer |
| C076-12 | 1 | **2.97±0.02** | Czech Republic, distr. Děčín, Brtníky, puddle on forest path S of village | 50°56'32"N, 14°26'14.9"E | 455 | puddle |  | 13 Sept. 2012 J. Hadinec & P. Bauer |
| C092-12 | 1 | 3.01 | Czech Republic, distr. Domažlice, Babylon, ruts on crossroads of forest paths near settlement Na Pohodnici | 49°24'18.4"N, 12°52'38.4"E | 520 | puddle |  | 29 Oct. 2013 JP |
| C002-13 | 2 | 3.01 | Czech Republic, distr. Mělník, Želízy, Želízská svodnice ditch in village | 50°25'21.1"N, 14°27'51.2"E | 176 | ditch |  | 3 May 2013 JP |
| C003-13 | 3 | **3.03±0.03** | Czech Republic, distr. Mělník, Tupadly, locality Mokřady Dolní Liběchovky, pool in valley of Liběchovka stream | 50°26'12"N, 14°28'13"E | 180 | pool |  | 3 May 2013 JarR |
| C012-13 | 1 | 3.01 | Czech Republic, distr. Nymburk, Kněžičky, puddle on forest path in deer-park Kněžičky S of village | 50°09'35.6"N, 15°20'39.3"E | 245 | puddle |  | 29 May 2013 JP |
| C018-13 | 1 | 3.03 | Czech Republic, distr. Nymburk, Loučeň, pool on bottom of long-term drained fishpond Lutovník | 50°17'34.6"N, 15°0'9.0"E | 234 | pool |  | 7 June 2013 JP |
| C020-13 | 1 | 3.01 | Czech Republic, distr. Příbram, Mokrovraty, puddle on meadow path NE of hill Dolní Jezberná | 49°48'43.8"N, 14°14'44.7"E | 335 | puddle | *C. palustris* | 11 June 2013 JP & KK |
| C024-13 | 1 | 3.07 | Czech Republic, distr. Tábor, Hrnčíře, puddle on forest path NW of village | 49°35'04.8"N, 14°51'11.2"E | 453 | puddle |  | 18 June 2013 KK |
| C035-13 | 1 | 2.97 | Czech Republic, distr. Jindřichův Hradec, Nová Olešná, puddles on forest path below dam of Vydloubal fishpond | 49°09'50.0"N, 15°09'41.0"E | 554 | puddle |  | 27 June 2013 ZK |
| C040-13 | 1 | 2.97 | Czech Republic, distr. Jičín, Mlázovice, puddle on forest path in Kamenický forest WNW of village | 50°25'11.7"N, 15°30'9.0"E | 315 | puddle |  | 4 July 2013 M. Štefánek |
| C041-13 | 1 | 2.97 | Czech Republic, distr. Trutnov, Kocbeře, puddle on path on forest glade E of village | 50°27'05.7"N, 15°52'13.4"E | 448 | puddle |  | 4 July 2013 JP |
| C042-13 | 1 | 2.95 | Czech Republic, distr. Trutnov, Kocbeře, puddle on forest path between village and Rabiš fishpond | 50°27'09.2"N, 15°52'42.4"E | 470 | puddle | *C. palustris* | 4 July 2013 JP |
| C053-13 | 1 | **2.97±0.01** | USA, Oregon, Coos County, Coos Bay, Mingus Park, garden pond in park | 43°22'18.5"N, 124°13'26.4"W | 3 | reservoir |  | 29 July 2013 JP & T. Kávová |
| C054-13 | 3 | **2.99±0.03** | USA, Oregon, Coos County, Bandon, Lost Lake | 43°1'23.9"N, 124°25'51.8"W | 9 | lake |  | 30 July 2013 JP & T. Kávová |
| C062-13 | 1 | **2.94±0.05** | Czech Republic, distr. Cheb, Povodí, Lužní stream on confluence with Sázek stream | 50°8'57.4"N, 12°25'27.2"E | 436 | stream (sand) | *C. hamulata* | 19 July 2013 KK & JarR |
| C067-13 | 2 | **2.98±0.03** | Czech Republic, distr. Cheb, Dvorek, Stodolský stream by road bridge NE of village | 50°8'19.5"N, 12°24'42.5"E | 431 | stream (sand) | *C. hamulata, C. cophocarpa* | 19 July 2013 KK & JarR |
| C072-13 | 1 | **2.99±0.04** | Norway, prov. Møre og Romsdal, Runde Island, wet places on path on NW part of island | 62°24'29.0"N, 5°35'47.4"E | 246 | puddle |  | 7 Aug. 2013 KK |
| C078-13 | 2 | 2.98 | Czech Republic, distr. Liberec, Černousy, stream along second uppermost fishpond in pond cascade E of settlement V Poli | 50°59'35.3"N, 15°03'26.4"E | 242 | stream (mud) | *C. platycarpa* | 22 Aug. 2013 JP & KK |
| C087-13 | 1 | 3.05 | Czech Republic, distr. Cheb, Prameny, puddle on forest path NE–ENE of village | 50°03'41.3"N, 12°45'36.3"E | 760 | puddle |  | 21 Aug. 2013 J. Chrtek jr. |
| C089-13 | 1 | **3.04±0.03** | Czech Republic, distr. Rychnov nad Kněžnou, Petrovice, puddle on forest path SE of railway station | 50°10'17.4"N, 16°3'4.8"E | 250 | puddle |  | 1 Sept. 2013 JP & KK |
| C110-13 | 1 | 2.99 | Czech Republic, distr. Ústí nad Labem, Ostrov, small fishpond below dam of Ostrovský fishpond | 50°48'24"N, 14°2'47"E | 445 | fishpond |  | 28 Aug. 2013 JanR & JarR |
| C114-13 | 1 | 3.01 | Czech Republic, distr. Děčín, Maxičky, Vlčí jezero fishpond | 50°48'35"N, 14°8'0"E | 480 | fishpond |  | 28 Aug. 2013 JanR & JarR |
| C121-13 | 1 | 2.97 | Czech Republic, distr. Ústí nad Labem, Libouchec, artificial pool in locality Libouchecké rybníčky | 50°46'1"N, 14°3'32"E | 330 | pool |  | 27 Aug. 2013 JanR & JarR |
| C123-13 | 1 | 2.99 | Czech Republic, distr. Děčín, Zadní Ves, puddle on forest path near state border N of village | 50°48'35"N, 14°4'53"E | 507 | puddle |  | 28 Aug. 2013 JanR & JarR |
| C130-13 | 2 | 2.99 | Czech Republic, distr. Jihlava, Řídelov, ditch along forest path near SW bank of Pilný fishpond | 49°14'08.7"N, 15°23'52.6"E | 620 | ditch | *C. cophocarpa* | 8 Sept. 2013 JP |
| C134-13 | 1 | 3.04 | Czech Republic, distr. Praha-východ, Kostelec nad Černými Lesy, puddle on forest path SSW of town | 49°58'13.6"N, 14°50'47.7"E | 390 | puddle | *C. hamulata* | 9 Sept. 2013 JP |
| ***C. stagnalis* autotriploid** | | | | | | | | |
|  |  |  |  |  |  |  |  |  |
| C125-13 | 1 | **4.55±0.03** | Czech Republic, distr. Děčín, Zadní Ves, on forest path N of village | 50°48'26"N, 14°4'45"E | 525 | puddle |  | 28 Aug. 2013 JanR & JarR |
| ***C. ×vigens* K. Martinsson [*C. cophocarpa* × *C. platycarpa*]** | | | | | | | | |
|  |  |  |  |  |  |  |  |  |
| C058-08 | 3 | **4.69±0.01** | Czech Republic, distr. Cheb, Nový Drahov, settlement Hájek, Soos area, pool near spring Věra | 50°8'59.7"N, 12°23'59.3"E | 435 | pool |  | 3 Aug. 2008 JP |
| C017-09 | 3 | **4.72±0.04** | Czech Republic, distr. Česká Lípa, Mimoň, ditch in park | 50°39'26"N, 14°43'44"E | 280 | ditch |  | 8 June 2009 JP |
| C026-09 | 2 | **4.72±0.02** | Czech Republic, distr. Česká Lípa, Zahrádky, Peklo valley, side arm of Robečský stream | 50°38'37"N, 14°30'35"E | 250 | pool |  | 9 June 2009 JP |
| C030-09 | 3 | **4.72±0.05** | Czech Republic, distr. Litoměřice, Kostelec nad Ohří, locality Myslivna, ditch in floodplain forest | 50°23'42.6"N 14°05'0.6"E | 165 | ditch |  | 18 June 2009 JP |
| C011-10 | 3 | **4.65±0.04** | Czech Republic, Most, city distr. Kopisty, Bílina River | 50°32'31.3"N, 13°37'2.8"E | 227 | river (mud) |  | 2 June 2010 JP |
| C157-10 | 2 | **4.64±0.05** | Czech Republic, distr. Písek, Zátaví, right bank of Otava River near settlement Zátavský Mlýn | 49°16'20.9"N, 14°5'50.6"E | 365 | river (mud) |  | 29 Sept. 2010 JP |
| C016-11 | 5 | **4.62±0.03** | Czech Republic, distr. Klatovy, Čepice, blind arm on right bank of Otava River ESE of village | 49°15'57.5"N, 13°36'3.7"E | 445 | pool | *C. hamulata* | 17 June 2011 JP & P. Koutecký |
| C018-11 | 2 | 4.60 | Czech Republic, distr. Klatovy, Čepice, blind arm on leftbank of Otava River ESE of village | 49°15'58.2"N, 13°36'13.0"E | 445 | pool | *C. hamulata* | 17 June 2011 JP & P. Koutecký |
| C020-11 | 4 | 4.63 | Czech Republic, distr. Klatovy, Velké Hydčice, blind arm of Otava River N of village | 49°18'19.5"N, 13°40'9.8"E | 426 | pool | *C. hamulata* | 17 June 2011 JP & P. Koutecký |
| C022-11 | 1 | 4.55 | Czech Republic, distr. Klatovy, Týnec, on alluvium of Otava River below weir | 49°18'48.3"N, 13°40'34.6"E | 425 | exposed bottom |  | 17 June 2011 JP & P. Koutecký |
| C023-11 | 1 | 4.65 | Czech Republic, distr. Klatovy, Týnec, blind arm of Otava River E of village | 49°18'53.0"N 13°40'44.0"E | 425 | pool |  | 17 June 2011 JP & P. Koutecký |
| C035-11 | 1 | 4.66 | Czech Republic, distr. Strakonice, Pracejovice, blind arm of Otava River NE of village | 49°15'38.2"N, 13°51'34.4"E | 395 | pool |  | 18 June 2011 JP & P. Koutecký |
| C036-11 | 1 | 4.61 | Czech Republic, distr. Strakonice, Virt, locality Bažantnice u Pracejovic, blind arm of Otava River S of village | 49°15'41.8"N, 13°52'0.1"E | 390 | pool |  | 18 June 2011 JP & P. Koutecký |
| C037-11 | 3 | 4.69 | Czech Republic, Strakonice, town distr. Podskalí, blind arm on right bank of Otava River in camp | 49°15'26.2"N, 13°53'19.8"E | 390 | pool | *C. hamulata* | 19 June 2011 JP & P. Koutecký |
| C039-11 | 2 | **4.63±0.04** | Czech Republic, Strakonice, separated arm of Otava River on E edge of town | 49°15'30.0"N, 13°55'48.7"E | 385 | pool |  | 19 June 2011 JP & P. Koutecký |
| C021-12 | 5 | **4.64±0.02** | Czech Republic, distr. Mladá Boleslav, Dolení Kruhy, Jizera River NNW of village | 50°33'45.6"N, 14°59'41.3"E | 230 | river (sand) | *C. hamulata* | 14 June 2012 JP & PT |
| C041-12 | 2 | **4.62±0.02** | Denmark, Jutland, reg. Midtjylland, Nees, Søndersund lake | 56°23'57.0"N, 08°13'51.3"E | 0 | lake | *C. hamulata, C. platycarpa* | 1 Aug. 2012 JP & ZK |
| C055-13 | 1 | **4.62±0.07** | Czech Republic, distr. Cheb, Skalná, Soos area, lake E of settlement Kateřina | 50°9'21.2"N, 12°24'37.4"E | 447 | lake |  | 18 July 2013 KK & JarR |
| C056-13 | 1 | **4.64±0.06** | Czech Republic, distr. Cheb, Nový Drahov, settlement Hájek, Soos area, pool NE of railway station | 50°8'53.5"N, 12°24'0.5"E | 439 | pool |  | 18 July 2013 KK & JarR |
| C068-13 | 3 | **4.64±0.04** | Czech Republic, distr. Domažlice, Nemanice, blind arm of Nemanický stream SW of settlement Nemaničky | 49°25'48.6"N, 12°42'40.6"E | 504 | pool | *C. hamulata, C. platycarpa* | 10 Aug. 2013 M. Hrdinová |
| C082-13 | 7 | **4.63±0.03** | Czech Republic, distr. Jablonec nad Nisou, Bedřichov, water reservoir on N edge of village | 50°47'43.1"N, 15°8'34.8"E | 733 | reservoir |  | 22 Aug. 2013 JP & KK |
| C108-13 | 2 | **4.72±0.02** | Czech Republic, distr. Karlovy Vary, Sedlečko, blind arm of Ohře River NNE of settlement Hubertus | 50°14'27"N, 12°55'49"E | 365 | pool | *C. hamulata* | 29 Aug. 2013 JanR & JarR |
| C115-13 | 1 | **4.65±0.06** | Czech Republic, distr. Děčín, Maxičky, lowermost fishpond S of village | 50°48'19"N, 14°10'58"E | 405 | fishpond |  | 28 Aug. 2013 JanR & JarR |
| C116-13 | 1 | **4.67±0.05** | Czech Republic, distr. Děčín, city distr. Přípeř, fishpond close to Drážďanská street | 50°47'13"N, 14°12'10"E | 180 | fishpond |  | 26 Aug. 2013 JanR & JarR |
| C132-13a | 1 | 4.74 | Germany, Bayern, Hohenfels, Follerenbach stream between Hohenfels and Kalmünz villages | 49°11'7.2"N, 11°54'45.7"E | 364 | stream (sand) | *C. hamulata* | 7 Sept. KK |
| **hybrid from Tichá Orlice river [probably *C. hamulata* × *C. cophocarpa*]** | | | | | | | | |
|  |  |  |  |  |  |  |  |  |
| C061-12 | 5 | **7.60±0.02** | Czech Republic, distr. Ústí nad Orlicí, Brandýs nad Orlicí, Tichá Orlice River below bridge of Žerotínova street | 50°0'4.9"N, 16°16'45.1"E | 295 | river (sand) | *C. cophocarpa, C. hamulata* | 15 Aug. 2012 JP |
| C065-12 | 1 | **7.61±0.08** | Czech Republic, distr. Ústí nad Orlicí, Choceň, Tichá Orlice River on SW edge of town | 49°59'43.2"N, 16°12'36.1"E | 270 | river (sand) | *C. hamulata* | 15 Aug. 2012 JP |
| C066-12 | 6 | **7.64±0.08** | Czech Republic, distr. Ústí nad Orlicí, Choceň, millrace of Tichá Orlice River on NW edge of town | 50°0'4.5"N, 16°12'13.2"E | 270 | stream (sand) | *C. hamulata* | 15 Aug. 2012 JP |
